# Zebrafish are resilient to the loss of major diacylglycerol acyltransferase enzymes

**DOI:** 10.1016/j.jbc.2024.107973

**Published:** 2024-11-06

**Authors:** Meredith H. Wilson, Monica R. Hensley, Meng-Chieh Shen, Hsiu-Yi Lu, Vanessa H. Quinlivan, Elisabeth M. Busch-Nentwich, John F. Rawls, Steven A. Farber

**Affiliations:** 1 Department of Biology, Johns Hopkins University, Baltimore, Maryland, USA; 2 Department of Embryology, Carnegie Institution for Science, Baltimore, Maryland, USA; 3 Department of Molecular Genetics and Microbiology, Duke Microbiome Center, Duke University, Durham, North Carolina, USA; 4 School of Biological and Behavioural Sciences, Queen Mary University of London, London, UK

**Keywords:** acyltransferase, dgat, lipid droplet, lipoprotein secretion, membrane enzyme, mogat3, mutant, yolk syncytial layer, zebrafish

## Abstract

In zebrafish, maternally deposited yolk is the source of nutrients for embryogenesis prior to digestive system maturation. Yolk nutrients are processed and secreted to the growing organism by an extra-embryonic tissue, the yolk syncytial layer (YSL). The export of lipids from the YSL occurs through the production of triacylglycerol-rich lipoproteins. Here we report that mutations in the triacylglycerol synthesis enzyme, diacylglycerol acyltransferase-2 (Dgat2), cause yolk sac opacity due to aberrant accumulation of cytoplasmic lipid droplets in the YSL. Although triacylglycerol synthesis continues, it is not properly coupled to lipoprotein production as *dgat2* mutants produce fewer, smaller, ApoB-containing lipoproteins. Unlike DGAT2-null mice, which are lipopenic and die soon after birth, zebrafish *dgat2* mutants are viable, fertile, and exhibit normal mass and adiposity. Residual Dgat activity cannot be explained by the activity of other known Dgat isoenzymes, as *dgat1a;dgat1b;dgat2* triple mutants continue to produce YSL lipid droplets and remain viable as adults. Further, the newly identified diacylglycerol acyltransferase, Tmem68, is also not responsible for the residual triacylglycerol synthesis activity. Unlike overexpression of Dgat1a and Dgat1b, monoacylglycerol acyltransferase-3 (Mogat3b) overexpression does not rescue yolk opacity, suggesting it does not possess Dgat activity in the YSL. However, *mogat3b;dgat2* double mutants exhibit increased yolk opacity and often have structural alterations of the yolk extension. Quadruple *mogat3b;dgat1a;dgat1b;dgat2* mutants either have severely reduced viability and stunted growth or do not survive past 3 days post fertilization, depending on the *dgat2* mutant allele present. Our study highlights the remarkable ability of vertebrates to synthesize triacylglycerol through multiple biosynthetic pathways.

In zebrafish, maternally deposited yolk is the source of nutrients for development prior to digestive system maturation. Liver-derived yolk proteins, oligosaccharides, and lipids are deposited into oocytes and stored in yolk platelets/granules (1, 2, 3). During embryogenesis, these stored nutrients are released into the yolk syncytial layer (YSL), an extra-embryonic cytoplasmic compartment surrounding the yolk mass that metabolizes, re-packages, and exports the nutrients to the developing embryo (4, 5, 6, 7, 8). Similar to the intestine and liver, the export of lipids from the YSL occurs through the production and secretion of triacylglycerol-rich lipoproteins (7, 8, 9, 10, 11, 12).

Phospholipids and neutral lipids, such as triacylglycerols (TAG) and cholesterol esters, are synthesized in the membrane of the endoplasmic reticulum (ER) (13, 14, 15). In the digestive tissues, the lipids are transferred by microsomal triglyceride transport protein (MTP) to Apolipoprotein B (ApoB) in the ER lumen to form lipoproteins (16, 17). Following additional processing/maturation in the Golgi apparatus, the TAG-rich ApoB-containing lipoproteins (B-lp) are secreted into the circulation for delivery of lipids to the body (18). Alternatively, synthesized lipids can be stored in cytoplasmic lipid droplets (LDs) (19). These organelles are generated when neutral lipids accumulate within the ER bilayer and then bud outward into the cytoplasm, creating an organelle with a core of hydrophobic neutral lipids coated by a monolayer of phospholipids (20, 21). Although substantial progress has been made in the last few decades toward understanding the proteins and mechanisms involved in the production of lipoproteins and lipid droplets (22, 23, 24, 25, 26, 27, 28), we still lack sufficient clarity on how ER triacylglycerol is routed to lipoproteins or cytoplasmic lipid droplets in digestive organs.

While both secretion and storage of lipids occurs regularly in the intestine and liver, the neutral lipids synthesized in the ER of the zebrafish YSL are almost exclusively packaged into lipoproteins for secretion, as cytoplasmic lipid droplets are very rare in wild-type animals (29, 30). However, we have previously shown that reducing lipoprotein production, by inhibiting the activity of MTP, results in the aberrant accumulation of lipid droplets in the YSL (30). When present in abundance, these lipid droplets refract light, causing the yolk sac to become opaque (30). Since this initial discovery, we have sought out additional zebrafish mutants exhibiting yolk opacity, with the hypothesis that we will discover additional proteins involved in the mechanisms influencing lipid secretion *versus* storage. Through this approach, we identified an opaque yolk mutant from the Zebrafish Mutation Project collection (31) that contains a premature termination codon in the second exon of the gene encoding the triacylglycerol synthesis enzyme, diacylglycerol acyltransferase 2 (*dgat2*).

Animals have two major pathways for TAG biosynthesis, the glycerol phosphate (Kennedy) pathway, present in all cells, and the monoacylglycerol pathway, which is predominantly active in the small intestine and liver (32, 33, 34, 35, 36, 37). In the glycerol phosphate pathway, a cascade of glycerol-3 phosphate acyltransferase, acylglycerol-3-phosphate acyltransferase, and phosphatidate phosphatase enzymes generate diacylglycerol, whereas in the monoacylglycerol pathway, diacylglycerol is generated through the action of monoacylglycerol acyltransferase (MGAT/MOGAT) enzymes. In the final reaction of both pathways, acyl-CoA:diacylglycerol acyltransferase (DGAT) enzymes catalyze the acylation of diacylglycerol with a fatty acyl-CoA, to form triacylglycerol (34). There are two major DGAT enzymes in animals, DGAT1 (38) and DGAT2 (39, 40). They are members of two distinct gene families and do not share structural or sequence homology (41). Though they are often present in the same cell types and catalyze the same reaction, they exhibit distinct biochemical and cellular properties and the loss of each protein leads to different physiological consequences (38, 39, 42, 43, 44, 45, 46, 47, 48). The zebrafish and other teleost genomes contain two ohnologs of *dgat1* (annotated as *dgat1a* and *dgat1b)*, and a single *dgat2* gene (41); however, the localization and function of these proteins in zebrafish have not been described.

While mice deficient in DGAT1 are viable and fertile, exhibiting only moderate reductions in tissue TAG (49), mice lacking DGAT2 have severe reductions in tissue TAG and die 2 to 24 h after birth due in part to transepidermal water loss resulting from dysfunction of the skin permeability barrier (42, 50, 51). Both DGAT1 and DGAT2 are typically found in lipoprotein-producing tissues. Based on protein topology (52, 53) and localization of DGAT enzymatic activity on both the cytoplasmic and lumenal sides of the ER membrane (54), it was suggested that DGAT1 produces TAG for secretion and DGAT2 channels TAG to storage (55). However, numerous *in vitro* and *in vivo* studies in intestinal enterocytes and liver hepatocytes indicate that DGAT activity and TAG partitioning in these tissues are far more complex and that both DGAT enzymes contribute to secretion and storage (44, 56, 57, 58, 59, 60, 61, 62, 63).

Typically, reducing DGAT protein levels or chemically inhibiting DGAT activity leads to reduced TAG storage, fewer lipid droplets, or altered sizes of lipid droplets (44, 58, 63, 64, 65, 66). Thus, it was unexpected that the yolk opacity in the zebrafish *dgat2* mutants results from the accumulation of lipid droplets in the YSL. Though triacylglycerol is still produced in these mutants, it is channeled improperly into storage, resulting in reduced quantity and size of ApoB-containing lipoproteins. Unlike *Dgat2*-null mice, zebrafish *dgat2* mutants are viable, healthy, and fertile as adults. These data suggest that another enzyme in the YSL synthesizes TAG, but is unable to efficiently couple synthesis to secretion. In an approach to identify this enzyme, we generated mutant fish lacking *mogat3b*, *dgat1a*, and *dgat1b* in the background of the *dgat2* mutants. Quadruple homozygous mutant embryos continue to exhibit aberrant YSL lipid droplets, suggesting the existence of an additional yet-to-be-revealed TAG synthesis enzyme. However, this unidentified TAG synthesis enzyme is not sufficient to sustain life in the combined absence of Mogat3b, Dgat1a, Dgat1b & Dgat2, as the quadruple mutant fish are not viable past embryonic stages. Our data indicate that although there is substantial redundancy between these four enzymes, at least one must be present for survival. In sum, we provide evidence for the existence of an ER triacylglycerol channeling system supporting ApoB-containing lipoprotein synthesis in the zebrafish yolk syncytial layer, which is heavily dependent on Dgat2.

## Results

### Zebrafish *dgat2* mutant embryos exhibit yolk opacity due to accumulation of aberrant lipid droplets

**Figure 1:**
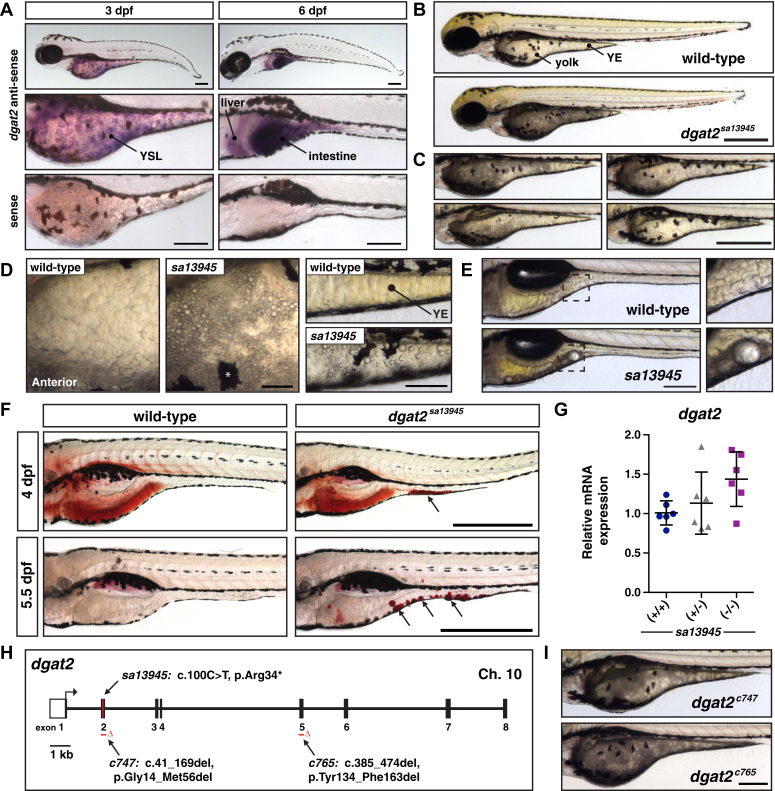
**Mutations in *dgat2* cause yolk opacity due to aberrant lipid droplet accumulation.***A*, *in situ* hybridization for *dgat2* expression at 3 and 6 days post fertilization (dpf) in wild-type AB embryos; *dgat2* is expressed in the yolk syncytial layer (YSL), liver and intestine; images are representative of all embryos from three experiments at each stage with n = 10 embryos per probe per experiment; Scale bars = 200 μm. *B*, representative images of a wild-type embryo and a homozygous mutant *dgat2*^*sa13945*^ embryo with a fully opaque yolk at 3 dpf; Scale = 500 μm. *C*, Examples of the heterogeneity in yolk opacity in *dgat2*^*sa13945*^ mutant embryos at 3 dpf; Scale = 500 μm. *D*, higher magnification images of the yolk in wild-type and *dgat2*^*sa13945*^ mutant embryos at 3 dpf highlighting visible accumulation of droplets; *left*: anterior yolk, *right*: yolk extension (YE), ∗ melanocyte; Scale = 50 μm. *E*, *dgat2*^*sa13945*^ larvae at 6 dpf sometimes retain yolk and/or large droplets; *right* shows magnification of noted regions; Scale = 200 μm. *F*, images of wild-type and *dgat2*^*sa13945*^ mutant embryos stained with Oil Red O to visualize neutral lipid. Arrows point to lipid droplets; images are representative of embryos from three experiments at each stage with n = 8 to 10 embryos per experiment; Scale = 500 μm. *G*, quantitative RT-PCR for *dgat2* expression in wild-type, *dgat2*^*sa13945/+*^, and *dgat2*^*sa13945*^ embryos at 3 dpf (N = 6; 10 pooled fish per sample/genotype, one-way ANOVA, *p* = 0.0844). *H*, depiction of the *dgat2* gene structure highlighting the nature and locations of the *c747* and *c765* CRISPR/Cas9 mutations in addition to the *sa13945* mutation in *dgat2* [GRCz11; ENSDARG00000018846, transcript 201 (ENSDART00000066793.7)], for more detail, see Fig. S2. *I*, representative images of yolk opacity in homozygous mutant *dgat2*^*c747*^ and *dgat2*^*c765*^ embryos at 3 dpf; scale = 200 μm.

DGAT2 is a member of the gene family that also contains acyl-CoA:monoacylglycerol acyltransferases (MGATs) and wax synthases. It is predicted to be an integral membrane protein that associates with the ER *via* a hydrophobic domain and contains a highly conserved HPHG sequence motif, the putative active site, which localizes to the cytosolic leaflet of the ER (53). *DGAT2* genes are nearly ubiquitous in eukaryotes and previous comparative genomic analyses (41, 67) indicate that the zebrafish genome contains a single *dgat2* gene (GRCz11; ENSDARG00000018846; 64.4% identity with human DGAT2). *In situ* hybridization analysis reveals that *dgat2* mRNA is expressed in the embryonic yolk syncytial layer, as well as the liver and intestine in zebrafish larvae (Fig. 1, *A*, Fig. S1).

The *dgat2*^*sa13945*^ mutation was generated in an ENU-based genetic screen (31) and is a nonsense mutation in exon 2 of 8 (c.100 C > T, p.Arg34∗) (Fig. S2, *A–C*). The mutant allele is present in Mendelian ratios (24.31 ± 3.60% homozygous mutants, mean ± SD, N = 3 clutches), and homozygous *dgat2*^*sa13945*^ fish are viable and fertile. The *dgat2*^*sa13945*^ mutant embryos present with yolk opacity similar to *MTP* mutants (30), with the yolk sac appearing dark with transmitted light microscopy. However, the phenotype is not fully penetrant (21.9% ± 6.9% opaque, mean ± SD, N = 34 heterozygous in-crosses,) and there is a variable degree of yolk opacity. Some fish have completely opaque yolks, some have patches of opacity either in the anterior yolk sac or in the yolk extension (YE), or both (Fig. 1, *B* and *C*) and some homozygous mutants have no visible phenotype at all. Maternal-zygotic mutant embryos resulting from homozygous *dgat2*^*sa13945*^ mutant in-crosses also display incomplete penetrance (85.7% ± 18.9% opaque, mean ± SD, N = 13 homozygous in-crosses) and range in phenotypic severity, indicating that the variance is not due to differences in maternal *dgat2* mRNA levels between embryos.

At higher magnification (Fig. 1, *D*), it is possible to see accumulations of spherical objects in the areas that are opaque at low magnification. These are reminiscent of the lipid droplets that accumulate in the yolk syncytial layer of *MTP* mutant fish (30). As the *dgat2*^*sa13945*^ fish continue to develop and consume their yolk, some larvae retain these droplets, which may become many microns in size (Fig. 1, *E*). To confirm whether these spherical objects are lipid droplets, we performed Oil Red O staining. At 3 & 4 days post fertilization (dpf), the yolk is still present and lipid-rich, so it stains red in both WT and mutants (Fig. 1*F*). However, some areas in the yolk extension of *dgat2*^*sa13945*^ mutants stain a deeper red, suggestive of accumulated lipid droplets. At 5.5 dpf, whereas wild-type fish have consumed their yolk and have no neutral lipid staining, some of the *dgat2*^*sa13945*^ fish still exhibit large red lipid droplets, often in the area of the yolk extension (Fig. 1, *F*).

In mammalian cells and tissues, the loss of DGAT2 activity results in fewer or smaller lipid droplets in cells (44, 58, 63, 65, 68), so it was surprising that *dgat2*^*sa13945*^ zebrafish mutants appear to accumulate ectopic lipid droplets in the YSL. Though we expected that the *sa13945* mutant allele would result in nonsense-mediated decay of the mRNA transcript, both qPCR analysis (Fig. 1, *G*) and *in situ* hybridization (Fig. S2*D*) reveal no loss of the mutant transcript. Because the *sa13945* mutation was generated in a chemical-based genetic screen, each mutant family will have a variety of missense, nonsense, and essential splice site mutations due to the nature of ENU mutagenesis. The mutant families in the Zebrafish Mutation Project screen were shown on average to contain seven nonsense, three essential splice site mutations, and 90 nonsynonymous mutations (31). Thus, to confirm that the nonsense mutation in *dgat2* causes the yolk opacity phenotype, we used CRISPR/Cas9 to generate two additional mutant alleles of *dgat2*.

Both an in-frame deletion of exon 2 that eliminates part of the putative neutral lipid-binding domain (*dgat2*^*c747*^), and an in-frame deletion within exon 5 that eliminates the enzymatic HPHG motif (*dgat2*^*c765*^), result in yolk opacity (*c747/+* in-crosses 23.8 ± 5.4% opaque, N = 16; *c765/+* in-crosses 23.4 ± 4.9% opaque, N = 22) (Fig. 1, *H* and *I*; Fig. S3). Furthermore, these CRISPR/Cas9 mutants fail to complement the *dgat2*^*sa13945*^ mutation (Fig. S3; *c747/+* x *sa13945/+*: 25.9 ± 4.6% opaque, N = 12 crosses; *c765/+* x *sa13945/+*: 26.5 ± 5.9% opaque, N = 3 crosses, see Supporting Information File 4 for source data). Together, these data strongly argue that the yolk opacity phenotype results from mutations in the *dgat2* gene. Additionally, expressing a FLAG-tagged *dgat2* construct in embryos can partially rescue the opacity phenotype (Fig. S4).

### Cytoplasmic lipid droplets and swollen, electron-dense ER are present in the yolk syncytial layer of *dgat2* mutants

**Figure 2:**
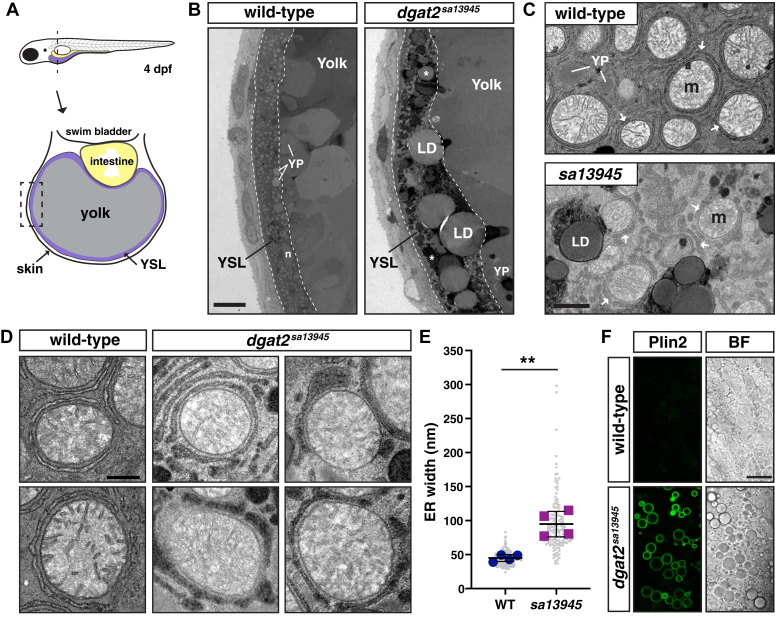
**Cytoplasmic lipid droplets and swollen, electron-dense ER are present in the yolk syncytial layer of *dgat2* mutants.***A*, depiction of the cross-sectional view of a 4 dpf zebrafish illustrating the yolk syncytial layer (YSL) surrounding the yolk mass. The dashed box indicates the location of the images in panel B. *B*, representative transmission electron micrographs of the yolk and YSL from wild-type and *dgat2*^*sa13945*^ mutants; dashed lines delineate the YSL region, n = nucleus, YP = yolk platelet, LD and ∗ = lipid droplet, scale = 10 μm. *C*, higher magnification images of the YSL region. Arrows indicate the endoplasmic reticulum which often encircles the mitochondria (m) in both wild-type and *dgat2*^*sa13945*^ mutants; scale = 1 μm. *D*, examples of ER morphology in wild-type and *dgat2*^*sa13945*^ embryos; scale = 0.5 μm. *E*, quantification of ER width, N = 4 fish per genotype (30–40 measurements per fish from 3 to 4 images, mean of each fish is shown by the blue and magenta points); bars indicate overall mean ± SD, unpaired *t* test, ∗∗*p* = 0.0022. *F*, representative confocal z-sections of the YSL in a wild-type fish and *dgat2*^*sa13945*^ mutant carrying the *Fus(EGFP-plin2)/+* reporter; scale = 20 μm, 3 dpf, n = 18 *dgat2*^*sa13945*^ & n = 15 WT or *dgat2*^*sa13945/+*^ fish from two independent experiments.

To confirm that the lipid droplets accumulating in the yolk sac of the *dgat2*^*sa13945*^ mutants are located within the cytoplasm of the YSL, we did transmission electron microscopy on embryos at 4 dpf, taking cross-sections through the yolk sac (Fig 2, *A*). Wild-type fish rarely have lipid droplets in the YSL, however, *dgat2*^*sa13945*^ mutants accumulate large numbers of lipid droplets (Fig 2, *B* and *C*).

We also noted that the ER, which often wraps around mitochondria, is wider and more electron dense in the *dgat2*^*sa13945*^ mutants than in wild-type fish (Fig. 2, *C*–*E*), perhaps suggesting accumulation of lipids in the ER. The wrapping of the ER around the mitochondria has been previously noted in the zebrafish liver and YSL (69) and more recently this organization of ER was shown to be important for regulating systemic lipid homeostasis and biogenesis of VLDL (70). The swollen ER phenotype in the mutants also suggested possible ER stress. However, qPCR of whole embryos did not reveal increases in the expression of typical ER stress marker genes, *ddit3/chop* & *bip* (Fig. S5*A*). To further confirm whether there is elevated ER stress in the YSL, we crossed the *dgat2*^*sa13945*^ mutants to an ER stress reporter line, *Tg(5xATF6RE:d2GFP)*, which drives expression of a destabilized GFP under the control of ATF6 response elements (71). While tunicamycin treatment elicits GFP fluorescence in the YSL, *dgat2*^*sa13945*^ mutants show no significant increase in GFP fluorescence when compared to wild-type siblings, again suggesting that ER stress is not present in the mutants (Fig. S5, Band C).

To verify that the lipid droplets are cytoplasmic and not enclosed within the lumen of the ER, we crossed the *dgat2*^*sa13945*^ mutant fish to our Perilipin 2 (Plin2) reporter line, *Fus(EGFP-plin2)* (72). Plin2 is a lipid droplet-associated protein that binds to the phospholipid monolayer of lipid droplets in the cytoplasm (73). Confocal imaging of the *dgat2*^*sa13945*^*;Fus(EGFP-plin2)* mutant YSL at 3 dpf shows that many of the lipid droplets are decorated with Plin2, indicating that the mutants indeed accumulate cytoplasmic lipid droplets (Fig. 2, *F*). Only rare Plin2-positive lipid droplets are present in the YSL of heterozygous and wild-type embryos.

### *dgat2* mutant embryos are still capable of synthesizing triacylglycerol in the YSL

The unexpected appearance of cytoplasmic lipid droplets in the YSL of *dgat2*^*sa13945*^ mutants suggests that neutral lipids (triacylglycerols, cholesterol esters, or both) are being abnormally retained in the yolk sac instead of being secreted to the developing embryo. We wondered whether the *dgat2*^*sa13945*^ mutants can still synthesize triacylglycerol in the YSL and whether TAG is found within the ectopic YSL lipid droplets in the mutants.

**Figure 3:**
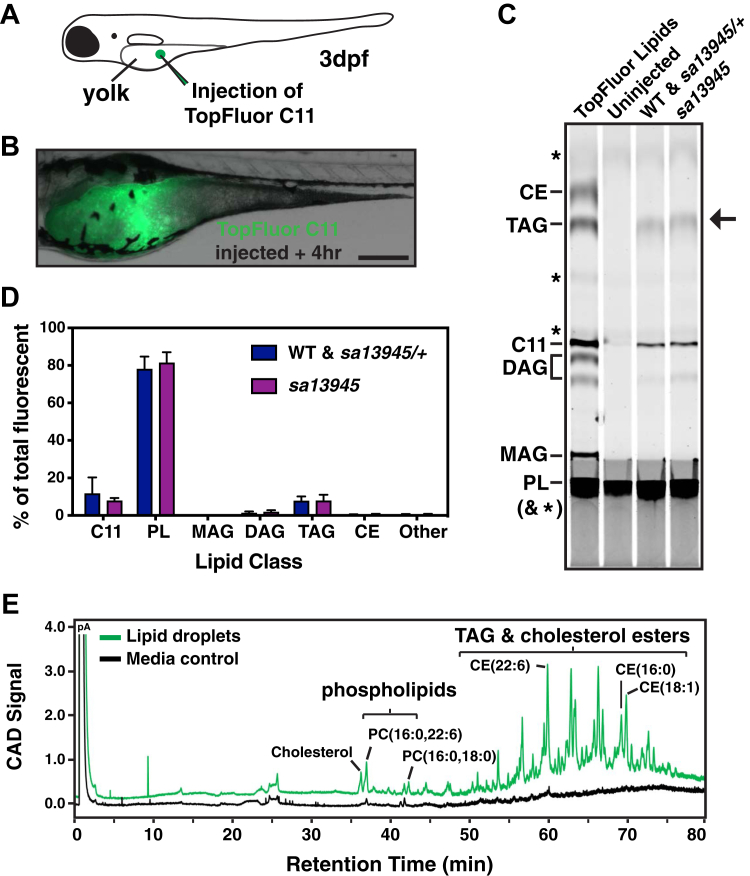
***dgat2* mutant embryos are still capable of triacylglycerol synthesis in the YSL.***A*, TopFluor C11 fatty acid was mixed with canola oil and microinjected into the yolk mass of *dgat2*^*sa13945*^ mutants and siblings at 3 dpf. *B*, representative image of the TopFluor C11 signal in a *dgat2*^*sa13945*^ zebrafish yolk sac 4 h after injection; scale = 200 μm. *C and D*, lipids were extracted from TopFluor C11 injected embryos and separated by thin layer chromatography (20 pooled embryos per sample, wild-type and *dgat2*^*sa13945/+*^ embryos were combined). *C*, lipid classes were identified using TopFluor-labeled lipid standards. Arrow indicates TAG synthesized from TopFlour C11 *in vivo*. (CE: cholesterol ester, TAG: triacylglycerol, DAG: diacylglycerol, MAG: monoacylglycerol; PL: phospholipid; ∗ denote autofluorescent lipids in the fish lysate). *D*, the quantity of lipids in each class was quantified and expressed as a percent of total fluorescent lipids; N = 6 experiments, mean ± SD, Two-way ANOVA, not significantly different, *p* = 0.9929. Note, it appears that the TopFluor C11 is unable to be incorporated into cholesterol esters. *E*, large lipid droplets were manually dissected out of *dgat2*^*sa13945*^ larvae at 6 dpf (40–50 LDs from 30 – 40 larvae). Lipids in LDs or an equivalent volume of media surrounding dissected larvae were separated by multistep gradient HPLC and detected with a charged aerosol detector (CAD) in picoamperes (pA) as described in (75). The lipid droplet samples contain large quantities of triacylglycerols and cholesterol esters. Traces are representative of three independent experiments.

We have shown previously that exogenously supplied fluorescent fatty acids can be utilized by the biosynthetic machinery in the YSL to synthesize phospholipid, cholesterol esters, and triacylglycerols (74). Therefore, to determine whether the *dgat2*^*sa13945*^ mutants are still able to synthesize triacylglycerol, we injected a fluorescently labeled TopFluor C11 fatty acid in canola oil into the yolks of *dgat2*^*sa13945*^ mutants and their wild-type and *dgat2*^*sa13945/+*^ siblings at 3 dpf (Fig 3, *A*). Immediately after injection, the TopFluor C11 fatty acid + canola oil appears in a bright sphere in the center of the yolk mass, but after 4 h much of the C11 fatty acid has moved outward into the YSL (Fig. 3, *B*). Lipids were extracted from groups of either mutant or normal-yolk sibling embryos, separated by thin-layer chromatography, and fluorescent lipids were detected with a biomolecular imager. Notably, the *dgat2*^*sa13945*^ mutants synthesize TopFluor-labeled triacylglycerols in similar quantities as their siblings (Fig 3, *C* and *D*).

To more directly assess whether the ectopic lipid droplets accumulating in the YSL of *dgat2*^*sa13945*^ mutants contain triacylglycerol, we manually dissected the large lipid droplets retained in the yolk sac remnants from mutant larvae at 6 dpf (Fig. 1, *E*). Lipids were extracted and analyzed using high-performance liquid chromatography (HPLC) using a previously described protocol (75). In comparison to the media control collected from around the dissected larvae, the lipid droplet samples were found to contain abundant phospholipids, sterol esters, and triacylglycerols (Fig. 3, *E*). Together this data argues that the *dgat2*^*sa13945*^ mutants can still synthesize triacylglycerol in the YSL.

### *dgat2* mutant embryos produce fewer, abnormally small ApoB-containing lipoproteins

**Figure 4:**
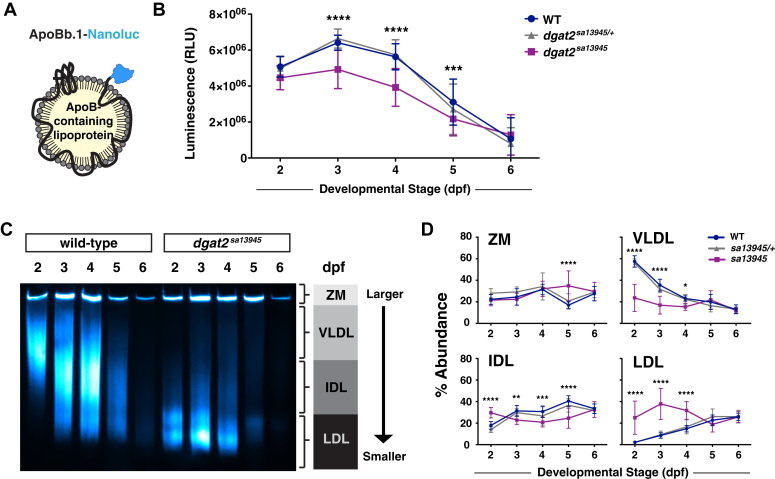
**Dgat2 mutants produce fewer, abnormally smallApoB-containing lipoproteins.***A*, LipoGlo fish express ApoBb.1 with a C-terminal Nanoluciferase enzyme fusion (11)[Lipoprotein image re-used from (30) under the CC BY 4.0 license]. *B*, LipoGlo luminescence (RLU = relative luminescence units) in WT, *dgat2*^*sa13945/+*^ and *dgat2*^*sa13945*^ fish throughout embryonic development (2–6 dpf). Results represent pooled data from three independent experiments, n = 21 to 38 (WT), n = 65 to 75 (*dgat2*^*sa13945/+*^), n = 32 to 43 (*dgat2*^*sa13945*^) fish/genotype/time-point; mean ± SD. Significance was determined with a two-way ANOVA (overall *p* < 0.0001 for genotype) and Bonferroni’s multiple comparison test was performed to compare genotypes at each day of development (∗∗∗∗*p* < 0.0001 *sa13945 versus* WT and *sa13945/+*, ∗∗∗*p* < 0.001 *sa13945 versus* WT). *C*, representative LipoGlo PAGE gel of wild-type and *dgat2*^*sa13945*^ mutants shows ApoB-containing lipoprotein (B-lp) size distribution from whole embryo lysates during development. B-lps are divided into four classes based on mobility, including zero mobility (ZM), very low density lipoproteins (VLDL), intermediate density lipoproteins (IDL) and low density lipoprotein (LDL). For heterozygote data, see original gels in Fig. S6. *D*, graphs show B-lp subclass abundance for WT, *dgat2*^*sa13945/+*^ and *dgat2*^*sa13945*^ fish at each day of embryonic development, analyzed from the gels in Fig. S6, as described in (11). Results represent pooled data from n = 9 fish/genotype/time-point; mean ± SD. For each particle class, significance was determined with a two-way ANOVA (overall *p* < 0.0280 ZM, *p* < 0.0001 VLDL, *p* < 0.0004 IDL, *p* < 0.0001 LDL for genotype) and Tukey’s multiple comparison test was performed to compare genotypes at each day of development (∗*p* < 0.05, ∗∗*p* < 0.01, ∗∗∗*p* < 0.001, ∗∗∗∗*p* < 0.0001 *sa13945 versus* WT are shown).

The absence of cytoplasmic lipid droplets in the YSL of wild-type zebrafish embryos suggests that the rates of yolk lipolysis, neutral lipid synthesis, and ApoB-containing lipoprotein production are tightly coordinated, such that newly synthesized triacylglycerols are packaged immediately for secretion. Zebrafish *MTP* mutants, which can’t efficiently transfer lipids from the ER membrane to nascent ApoB, exhibit reductions in both the number and the size of ApoB-containing lipoproteins, coupled with the accumulation of cytoplasmic lipid droplets in the YSL (30). While *dgat2*^*sa13945*^ mutants continue to synthesize triacylglycerol, the appearance of YSL lipid droplets suggests that the newly synthesized lipids may not be properly partitioned in the ER for lipoprotein production. To determine whether *dgat2*^*sa13945*^ mutants exhibit changes in B-lp production, we crossed the *dgat2*^*sa13945*^ mutants to our LipoGlo reporter line (11), which has an in-frame fusion of the nanoluciferase enzyme coding sequence at the C-terminus of the *apoBb.1* gene (Fig 4, *A*).

During embryonic and early larval development, B-lps are produced in large numbers by the YSL. Total B-lp numbers in whole-fish lysate increase from 2 to 3 dpf in wild-type fish, and then decline as the yolk lipid stores are depleted and the circulating lipoprotein lipids are utilized by the growing tissues of the body (Fig. 4, *B*). By 5 to 6 dpf, the intestine and liver have developed and can begin producing B-lps from these digestive tissues. However, over this developmental time-course, which is performed in the absence of exogenous food, we presume that the vast majority of B-lps originate in the YSL. Though *dgat2*^*sa13945*^ mutants exhibit a similar temporal pattern of lipoprotein abundance to their wild-type siblings, the total number of lipoproteins is blunted, which is especially notable at 3 and 4 dpf (Fig. 4, *B*). More strikingly, the size of the lipoproteins produced by the YSL is substantially reduced in *dgat2*^*sa13945*^ mutants when compared to heterozygous and wild-type siblings (Fig. 4, *C* and *D*, Fig. S6). This data suggests that the triacylglycerol synthesized in the *dgat2* mutants is abnormally shunted to storage in cytoplasmic lipid droplets rather than being packaged into ApoB-containing lipoproteins for secretion.

### *dgat2* mutants do not suffer lasting effects on size and adiposity

The reduction in B-lp number and size led us to question whether the *dgat2*^*sa13945*^ mutant fish would exhibit any adverse effects on growth and development. While we found that *dgat2*^*sa13945*^ mutants had initial reductions in musculature and concomitant increases in the yolk sac area at 3 dpf relative to their siblings, these differences were no longer apparent at 6 dpf (Fig. S7*A*). Furthermore, the mutation in *dgat2* does not result in any differences in standard length, mass, or body mass index (BMI) at 6 months of age (Fig. S7*B*), and we noted no differences in the development of adipose tissue (Fig. S7, *C–E*).

### Dgat1 is not responsible for the residual triacylglycerol synthesis in the *dgat2* mutants

The lack of a persisting effect on fish size, adiposity, and mass, together with the finding that *dgat2*^*sa13945*^ mutants can still synthesize triacylglycerol, suggests that another enzyme(s) compensates for the loss of Dgat2 activity. The reduced number and size of lipoproteins and the abnormal accumulation of cytoplasmic lipid droplets in the YSL imply that this alternate diacylglycerol acyltransferase is not able to properly direct the synthesized triacylglycerol toward lipoprotein production in the ER.

The most obvious candidate for this alternate enzyme is the other major DGAT enzyme, DGAT1, which belongs to the membrane-bound *O*-acyltransferase (MBOAT) family of proteins. DGAT1 is localized to the ER and contains multiple transmembrane domains (38). Zebrafish retained two *dgat1* ohnologs following the teleost-specific whole genome duplication, *dgat1a* (GRCz11; *dgat1a* ENSDARG00000103503) and *dgat1b* (GRCz11; ENSDARG00000054914) (41). The amino acid sequences have 63% identity with each other and 59 to 61% identity with human DGAT1; both share conserved binding motifs for fatty acids and diacylglycerol, as well as conserved catalytic residues and differ mostly in their N-terminal region (41, 67, 76, 77). Recent cryo-electron microscopy structures of human DGAT1 indicate that it functions as a homodimer (76, 77), and that the N-terminal region is required for oligomerization and enzymatic function (52, 77, 78). It is currently unknown whether zebrafish Dgat1a and Dgat1b can heterodimerize, given the differences in their N-terminal regions. No prior studies have described the tissue expression patterns of these two *dgat1* genes in zebrafish, so it is unclear whether the genes have differential tissue-specific or developmental expression patterns. Murine DGAT1 also catalyzes the synthesis of diacylglycerols, waxes, and retinyl esters (48), but it is unknown whether zebrafish Dgat1a and Dgat1b also harbor these activities, whether they exhibit differential substrate preferences, or whether they have differential localization in the cell.

**Figure 5:**
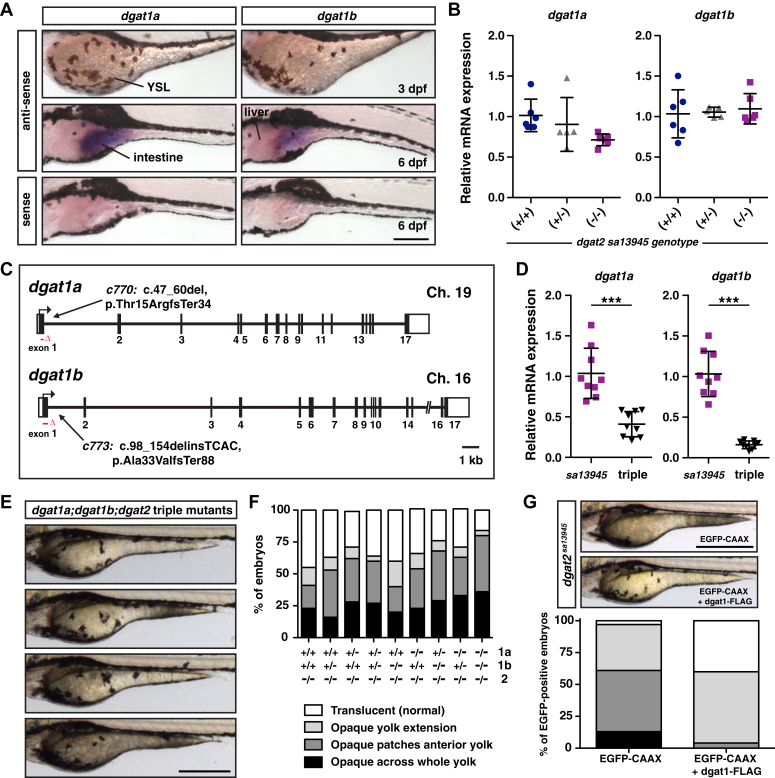
**Dgat1 is not responsible for the residual triacylglycerol synthesis in the *dgat2* mutants.***A*, *in situ* hybridization for *dgat1a* and *dgat1b* expression at 3 and 6 dpf in wild-type AB embryos. *dgat1a* and *dgat1b* are not detected in the YSL, but are expressed in the intestine; images are representative of all embryos from three experiments at each stage (n = 4–six embryos per probe per experiment); Scale bars = 200 μm. *B*, quantitative RT-PCR for *dgat1a* and *dgat1b* expression in wild-type, *dgat2*^*sa13945/+*^, and *dgat2*^*sa13945*^ embryos at three dpf (N = 5–6 clutches; 10 pooled fish per sample/genotype, mean ± SD, One-way ANOVA, *p* = 0.0887 *dgat1a*, *p* = 0.8773 *dgat1b*). *C*, depiction of the *dgat1a* and *dgat1b* gene structure highlighting the nature and locations of the *c770* and *c773* CRISPR/Cas9 mutations (GRCz11; *dgat1a* ENSDARG00000103503, transcript 201 (ENSDART00000158946.2), *dgat1b* ENSDARG00000054914, transcript 201 (ENSDART00000077185.5)). *D*, quantitative RT-PCR for *dgat1a* and *dgat1b* expression in *dgat2*^*sa13945*^*versus dgat1a*^*c770*^*;dgat1b*^*c773*^*;dgat2*^*sa13945*^ triple mutant embryos at 3 dpf (N = 3 independent experiments; n = 9 fish per genotype; mean ± SD, unpaired *t* test, ∗∗∗*p* < 0.001). *E*, examples of yolk opacity phenotypes in *dgat1a*^*c770*^*;dgat1b*^*c773*^*;dgat2*^*sa13945*^ triple mutant embryos at 3 dpf; Scale = 500 μm. *F*, embryos from in-crosses of *dgat1a*^*c770/+*^*;dgat1b*^*c773/+*^*;dgat2*^*sa13945*^ parents were imaged and scored at three dpf for the degree of yolk opacity, binned into the four noted categories prior to genotyping and expressed as a percent of total embryos per genotype (N = 4 independent experiments, n = 22–89 fish per genotype). *G*, *dgat2*^*sa13945*^ embryos were co-injected at the 1-cell stage with CMV:dgat1a-FLAG*,* CMV:dgat1b-FLAG and CMV:EGFP-CAAX plasmids, or CMV:EGFP-CAAX alone as a control. Bright-field images were obtained of all the embryos that expressed EGFP-CAAX in the YSL at 3 dpf; representative images of embryos from the two treatment groups (top, Scale = 500 μm). Images were assessed for the degree of yolk opacity, binned into the four noted categories of yolk opacity as noted in *F* and expressed as a percent of total EGFP-positive embryos/treatment group (n = 77 EGFP-CAAX and n = 77 dgat1-FLAG embryos pooled from three independent experiments, Chi-square test *p* < 0.0001).

Our *in situ* hybridization analysis suggests that neither *dgat1a* nor *dgat1b* mRNA are expressed in the zebrafish YSL, however, both genes are expressed in the intestine by 6 dpf (Fig. 5, *A*). Further, qRT-PCR for *dgat1a* and *dgat1b* indicates that their mRNA expression is not upregulated in the *dgat2*^*sa13945*^ mutants (Fig. 5, B, Fig. S8). While these data suggest that Dgat1 is not responsible for the residual triacylglycerol synthesis in the *dgat2*^*sa13945*^ mutants, we did not want to rule out the possibility that we could be missing very low levels of expression in the YSL. Consequently, to confirm that Dgat1a and/or Dgat1b were not active in the YSL, we generated CRISPR/Cas9 mutant alleles of both *dgat1* genes in the *dgat2*^*sa13945*^ mutant background. Both genes were targeted in exon 1, and the resulting *dgat1a*^*c770*^ mutant allele has a 14 bp deletion and the *dgat1b*^*c773*^ mutant allele has a 58 bp deletion + 4 bp insertion. Mutations were confirmed at the cDNA level and both alleles are predicted to result in frame shifts and premature termination in exon 1 and exon 4, respectively (Fig. 5, *C*, see Fig. S9 for more detail).

*dgat1a*^*c770*^*;dgat1b*^*c773*^*;dgat2*^*sa13945*^ triple mutants have significant reductions in mRNA expression of both *dgat1a* and *dgat1b*, when compared with *dgat2*^*sa13945*^ mutant siblings (Fig. 5, *D*), likely due to nonsense-mediated decay of the mutated mRNA transcripts. Neither single mutants of *dgat1a*^*c770*^ or *dgat1b*^*c773*^, nor *dgat1a*^*c770*^*;dgat1b*^*c773*^ double mutants exhibit yolk opacity (Fig. S10*A*).

We hypothesized that if Dgat1 was responsible for the residual triacylglycerol synthesis in the YSL of *dgat2*^*sa13945*^ mutants, then loss of Dgat1 activity in *dgat1a*^*c770*^*;dgat1b*^*c773*^*;dgat2*^*sa13945*^ triple mutants would prevent triacylglycerol synthesis, accumulation of cytoplasmic lipid droplets and yolk opacity, and might prevent or delay yolk utilization, possibly resulting in death of the embryos. However, when the *dgat1* mutations are present with *dgat2*^*sa13945*^, neither the mutation in *dgat1a*, *dgat1b* nor both together rescue the opacity phenotype, and instead, the triple mutants have a tendency toward a stronger yolk opacity phenotype (Fig. 5, *E* and *F*). Electron micrographs of triple mutants confirm the accumulation of YSL cytoplasmic lipid droplets (Fig. S10*B*).

These findings imply that Dgat1 is likely present in the YSL (albeit at levels undetectable by *in situ* hybridization) and may be aiding Dgat2 to synthesize triacylglycerol for export in lipoproteins, such that when Dgat1a/b are absent, even more triacylglycerol is deposited in lipid droplets, making the opacity phenotype worse. Supporting this hypothesis, injection of CMV:dgat1a-FLAG and CMV:dgat1b-FLAG plasmids into *dgat2*^*sa13945*^ mutants partially rescues yolk opacity (Fig. 5, *G*).

Despite the enhanced yolk opacity, ApoBb.1-nluc lipoprotein numbers are not different between *dgat2*^*sa13945*^ mutants and triple mutants at 3 dpf (Fig. S10*C*) and the larvae are no different in length (Fig. S10 *C and D*). Amazingly, the triple mutants survive to adulthood, are fertile, and no change in the standard length of the fish was noted at 6 months of age (Fig. S10*E*). Adult female triple mutants at 6 months were slightly larger in mass, resulting in a significant increase in BMI, but no changes were noted between the *dgat2*^*sa13945*^ and *dgat* triple mutant males (Fig. S10 *F and G*).

### Additional approaches to reveal the enzyme responsible for TAG synthesis in *dgat2* mutants did not yield a viable candidate

It was quite unexpected that zebrafish can survive in the absence of Dgat1 and Dgat2 enzymes. Further, lipid droplets persist in the YSL of *dgat* triple mutants, suggesting that triacylglycerol synthesis is still occurring. Thus, we assumed that there must be another enzyme responsible for this synthesis in the zebrafish. To elucidate the identity of this enzyme, we tried a number of different unbiased and targeted approaches.

First, we hypothesized that the enzyme of interest might be upregulated at the transcript level in *dgat2* mutants, so we performed RNA sequencing of wild-type siblings *versus* opaque *dgat2*^*sa13945*^ mutants. Although it was not possible to mechanically isolate the YSL, we enriched for YSL transcripts by trimming off heads and tails, thus focusing our analysis on the mid-bodies of the embryos at 3 dpf. In this analysis, 33 genes were upregulated and 21 genes were downregulated in the mutants compared to wild-type siblings (log2fold change >+/− 1.5, padj <0.05; Fig. S11). *dgat2* expression was not different between groups, confirming our earlier qPCR expression analysis (Fig. 1, *G*). Many of the differentially expressed genes were located on Chromosome 10, near the *dgat2*^*sa13945*^ mutation, suggesting that the expression of these genes may be altered in the mutants simply as a result of linkage to the *sa13945* locus (79). We also noted some changes in gene expression in the *dgat2* mutants that might have been anticipated, such as a small increase in lipoprotein lipase expression (*lpl*, log2fold change = 0.651) and decrease in *apoBb.2* expression (log2fold change = −1.20). However, none of the upregulated genes were compelling candidates for possessing acyltransferase activity.

Adipose triglyceride lipase (ATGL/PNPLA2) is a lipolytic enzyme that associates with lipid droplets and catalyzes the removal of a fatty acyl chain from triacylglycerol (80, 81). Notably, this enzyme has also been shown to act as a diacylglycerol transacylase, moving a fatty acyl chain between two molecules of diacylglycerol, yielding one triacylglycerol and one monoacylglycerol molecule (82, 83, 84, 85). To test whether the transacylase activity of Atgl is responsible for generating the triacylglycerol that is accumulating aberrantly in YSL lipid droplets, we treated the *dgat2*^*sa13945*^ mutant embryos with an inhibitor of ATGL, Atglistatin (86). While this inhibitor does not exhibit activity against ATGL in all species (87, 88), it has previously been shown to effectively inhibit zebrafish Atgl (89, 90). Imaging at 3 dpf after 48 h drug treatment did not reveal any differences in yolk opacity between the vehicle and Atglistatin-treated embryos, suggesting that Atgl is not responsible for generating the stored YSL triacylglycerol (Fig. S12).

### Mogat3b is not responsible for the residual triacylglycerol synthesis in the *dgat2* mutants

**Figure 6:**
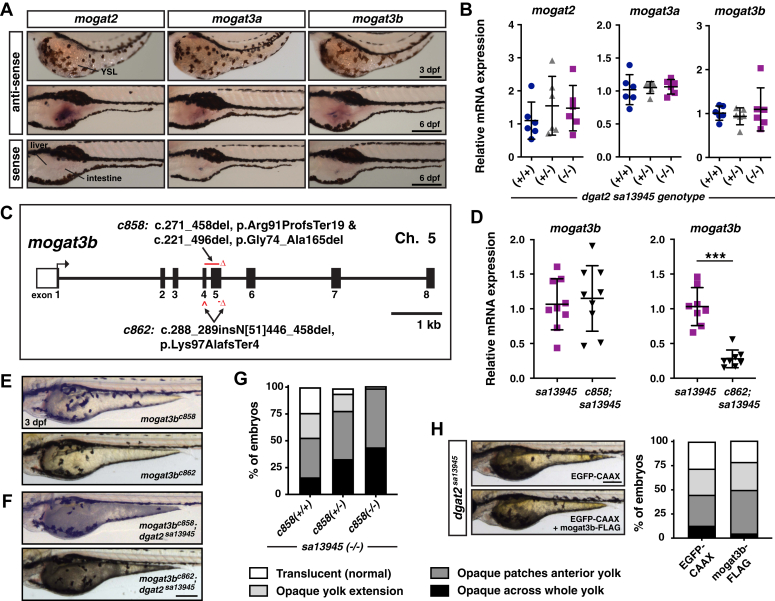
**Mogat3b is not responsible for the residual triacylglycerol synthesis in the *dgat2* mutants.***A*, *in situ* hybridization for *mogat2, mogat3a and mogat3b* expression at 3 and 6 dpf in wild-type AB embryos. *mogat* mRNA is not detected in the YSL, but is expressed in the intestine; images are representative of all embryos from three independent experiments at each stage (n = 4–6 embryos per probe per experiment); Scale bars = 200 μm. *B*, quantitative RT-PCR for *mogat2, mogat3a* and *mogat3b* expression in wild-type, *dgat2*^*sa13945/+*^, and *dgat2*^*sa13945*^ embryos at three dpf (N = 6 clutches; 10 pooled fish per sample/genotype, One-way ANOVA, *p* = 0.8820 *mogat1*, *p* = 0.5285 *mogat2, p* = 0.6988 *mogat3b*). *C*, depiction of the *mogat3b* gene structure highlighting the nature and locations of the *c858* and *c862* CRISPR/Cas9 mutations (GRCz11; *mogat3b* ENSDARG00000003635, transcript 201 (ENSDART00000015136.10)). *D*, quantitative RT-PCR for *mogat3b* expression in *dgat2*^*sa13945*^*versus mogat3b*^*c858*^*;dgat2*^*sa13945*^ or *mogat3b*^*c862*^*;dgat2*^*sa13945*^ double mutant embryos at 3 dpf (N = 3 independent experiments; 8–9 fish per genotype; unpaired *t* test, *p* = 0.6778 *c858*, ∗∗∗*p* < 0.001 *c862*). *E*, representative images of *mogat3b*^*c858*^ and *mogat3b*^*c862*^ homozygous mutant embryos at 3dpf; Scale = 200 μm. *F*, representative images of *mogat3b*^*c858*^*;dgat2*^*sa13945*^ and *mogat3b*^*c862*^*;dgat3*^*sa13945*^ double mutant embryos at 3 dpf; Scale = 200 μm. *G*, embryos from in-crosses of *mogat3b*^*c858/+;*^*dgat2*^*sa13945*^ parents were imaged and scored at 3 dpf for the degree of yolk opacity, binned into the four noted categories prior to genotyping and expressed as a percent of total embryos per genotype (N = 3 independent experiments, n = 62–130 fish per genotype, Chi-square test *p* < 0.0001). *H*, *dgat2*^*sa13945*^ embryos were co-injected at the 1-cell stage with CMV:*mogat3b-FLAG* and CMV:*EGFP-CAAX* plasmids, or CMV:*EGFP-CAAX* alone as a control. Bright-field images were obtained of all the embryos that expressed EGFP-CAAX in the YSL at 3 dpf; representative images of embryos from the two treatment groups (*left*, Scale = 200 μm). Images were assessed for the degree of yolk opacity, binned into the four noted categories of yolk opacity as noted in Fig. 6*G* and expressed as a percent of total EGFP-positive embryos/treatment group (n = 75 EGFP-CAAX and n = 119 *mogat3b*-FLAG embryos pooled from three independent experiments, Chi-square test *p* = 0.0923).

While Dgat1 and Dgat2 are the predominant enzymes that perform the final step of TAG synthesis, there is evidence in cultured cells and in human hepatic lysates that the monoacylglycerol acyltransferase (MGAT/MOGAT) enzymes can synthesize triacylglycerol in addition to diacylglycerol (32, 57, 91, 92, 93, 94, 95). The zebrafish genome encodes 3 *mogat* genes: *mogat2*, *mogat3a*, and *mogat3b* (GRCz11; *mogat3a* ENSDARG00000086481, *mogat2* ENSDARG00000019228, and *mogat3b* ENSDARG00000003635*).* However, it is unclear whether any of these zebrafish proteins exhibit DGAT activity. Zebrafish *mogat2* is orthologous to human *MOGAT2*, and zebrafish *mogat3a* and *mogat3b* are orthologs of human *MOGAT3* based on synteny (Fig. S13). However, based on phylogeny, Mogat3a is more similar to mammalian MOGAT1 and MOGAT2*,* whereas Mogat3b is more similar to human MOGAT3 (41) (Fig. S13). *In situ* hybridization analysis suggests that none of these genes are expressed in the YSL, but are expressed in the intestine later in development (Fig. 6*A*). qRT-PCR analysis indicates that *mogat2, mogat3a*, and *mogat3b* mRNA expression is not upregulated in the *dgat2*^*sa13945*^ mutants (Fig. 6*B*).

Given our findings that *dgat* triple mutants had a stronger yolk opacity phenotype despite no obvious gene expression of *dgat1a* and *dgat1b* in the YSL, we considered the possibility that there may still be low YSL expression of *mogat* genes. Therefore, we decided to generate an additional CRISPR/Cas9 mutant allele; we chose to target *mogat3b* because Mogat3b has the most protein sequence similarity to Dgat2 (Mogat3b *versus* Dgat2: 48.8% identity, 66.5% similarity; Mogat2 *versus* Dgat2: 45.7% identity, 60.7% similarity; Mogat3a *versus* Dgat2: 42.1% identity, 59.6% similarity) (Fig. S13*F*). This is similar to the relationship between Human MOGAT3 and DGAT2 (41, 93). Human MOGAT3 also exhibits the most Dgat activity *in vitro* (92) and it has not been as well studied in mammalian systems because it is a pseudogene in the mouse (95). We generated the *mogat3b* mutations in the *dgat2*^*sa13945*^ mutant background and aimed to eliminate the HPHG enzymatic motif in exon 5 by injecting a pair of guides targeting exons 4 and 5. We recovered two mutants, *c858* and *c862*. The *mogat3b*^*c858*^ mutant allele has a 276 bp deletion spanning the two exons, which results in two major cDNA products: one in which 188 bp is deleted across exons 4 to 5, causing a frame shift and premature termination codon, and a second product in which exons 4 & 5 are spliced out, causing an in-frame deletion of 92 amino acids including the HPHG motif. The *c862* allele has a 51 bp insertion in exon 4 (which is derived from the injected guide RNA scaffold), as well as a 13 bp deletion in exon 5. The insertion causes a frameshift, encountering a premature termination codon after three differential amino acids in exon 4 (Fig. 6*C*, see Fig. S14 for more detail). While the *mogat3b*^*c858*^*;dgat2*^*sa13945*^ double mutants have similar mRNA expression as *dgat2*^*sa13945*^ siblings likely due to the in-frame splicing of exon 4 & 5, the *mogat3b*^*c862*^*;dgat2*^*sa13945*^ double mutants exhibit reduced expression levels, probably due to nonsense-mediated decay (Fig. 6, *D*).

We found that *mogat3b*^*c858*^ and *mogat3b*^*c862*^ mutants do not exhibit yolk opacity (Fig. 6, *E*), and the addition of the *mogat3b* mutations to the *dgat2*^*sa13945*^ mutant background does not prevent yolk opacity (Fig. 6, *F*). Instead, double *mogat3b;dgat2* mutants have a stronger yolk opacity phenotype (Fig. 6, *G*). To determine if Mogat3b over-expression in the yolk could rescue triacylglycerol synthesis and yolk opacity in the *dgat2*^*sa13945*^ mutants, we injected *dgat2*^*sa13945*^ mutant embryos with a *CMV: mogat3b-FLAG* construct at the 1-cell stage. Unlike Dgat1a/b-FLAG (Fig. 5*G*), the expression of Mogat3b-FLAG does not rescue yolk opacity (Fig. 6, *H*), which suggests that Mogat3b does not act as a diacylglycerol acyltransferase in the zebrafish YSL. Together, this data argues that Mogat3b is not the enzyme responsible for making the aberrantly-stored triacylglycerol in the *dgat2*^*sa13945*^ mutants. However, the increased yolk opacity phenotype in the double mutants suggests that Mogat3b does play a role in YSL lipid biosynthesis.

### Loss of triacylglycerol biosynthetic enzymes sometimes alters yolk sac morphology

**Figure 7:**
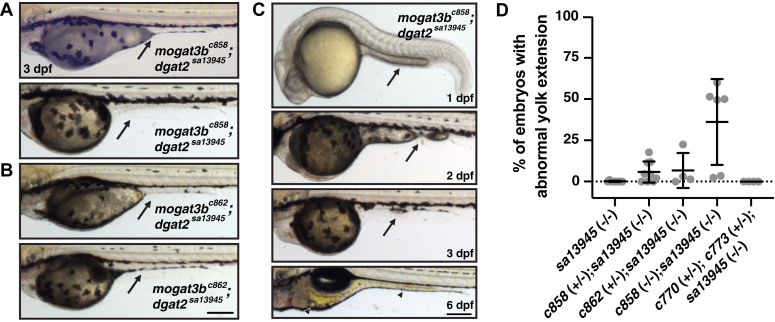
**Loss of triacylglycerol synthesis enzymes sometimes alters yolk sac morphology.***A,B*, representative images of *mogat3b*^*c858*^*;dgat2*^*sa13945*^ mutants (A) and *mogat3b*^*c862*^*;dgat2*^*sa13945*^ mutants (B) with short (*top*) or no (*bottom*) yolk extensions (YE) at 3 dpf. Arrows indicate where the yolk extension should be located. Scale = 200 μm. *C*, all *mogat3b*^*c858*^*;dgat2*^*sa13945*^ mutants initially have a YE, but the YE can be lost as the embryo develops (arrows). However, the gut develops normally (6 dpf), and sometimes large residual lipid droplets are noted (arrowheads). Scale = 200 μm. *D*, The percentage of embryos per clutch exhibiting abnormal yolk extension morphology (lack of YE, short YE, broken YE) on 3 dpf from in-crosses of the noted parental genotypes (N = 4–9 clutches per genotype (clutch size ranged from 20 to 300 embryos)).

In addition to the increase in yolk opacity in *mogat3b;dgat2*^*sa13945*^ double mutants, we find that double mutants sometimes present with abnormal yolk extension morphology (Fig. 7, *A* and *B*). At 3 dpf, some embryos have short or truncated yolk extensions, whereas others completely lack yolk extensions. To determine whether the yolk extension in these mutants is never formed or whether it is being abnormally lost during development, we imaged fish starting at earlier stages. We found that yolk extensions are present in all embryos at 1 dpf (Fig 7*C*, 1 dpf), even in clutches that later go on to contain fish lacking yolk extensions, indicating that the YE is forming properly (N = 4–9 clutches per genotype (clutch size ranged from 24–300 embryos)). However, by 2 dpf, we note that in some embryos, the yolk extension is already absent (Fig. S15*A*), while in others it appears to be constricted (Fig. 7*C*, 2 dpf). Further examination of where the yolk extension should be in embryos at 3 dpf, occasionally reveals what looks like remnants of yolk extension tissue (Fig. 7*C*, 3 dpf). Together, this suggests that the yolk in the extension may be utilized preferentially, or perhaps there are alterations in the structure of the yolk or YSL that cause the yolk to move from the YE forward into the anterior yolk ball. As the yolk mass behaves as a cohesive, viscoelastic foam, and yolk platelets rearrange in response to mechanical stress (96), we suspect that changes in mechanical tension either inside the YSL or outside the yolk could result in squeezing of the yolk extension contents forward. The yolk sac also tends to be more fragile in double mutants and is more likely to burst as a result of manipulation and/or temperature changes (*i.e.* cold methylcellulose mounting medium for live imaging). Regardless of the mechanism, embryos lacking yolk extensions exhibit normal morphology of their liver, pancreas and intestine at later larval stages (Fig. S7*C*, 6 dpf). As adults, the double *mogat3b;dgat2* mutants are similar in length and mass as their siblings (Fig. S15*B and C*).

The absent/short yolk extension phenotype is never present in *dgat2*^*sa13945*^ mutants and is also not found in the fish arising from in-crosses of *dgat1a*^*c770/+;*^*dgat1b*^*c773/+;*^*dgat2*^*sa13945*^ parents. Additionally, the abnormal yolk extension morphology is not fully penetrant in clutches from in-crosses of *mogat3b*^*c858*^*;dgat2*^*sa13945*^ double mutants, with some clutches exhibiting abnormal YE in over 50% of embryos, whereas other clutches have very few embryos with the phenotype (Fig. 7*D*). Further, we also noted this YE morphology defect in a small number of fish that were heterozygous for *mogat3b* (*mogat3b*^*c858/+;*^*dgat2*^*sa13945*^ embryos) (Fig. S15*D*).

### Quadruple *mogat3b*^*c858*^*;dgat1a*^*c770*^*;dgat1b*^*c773*^*;dgat2*^*sa13945*^ mutants have severely reduced viability

**Figure 8:**
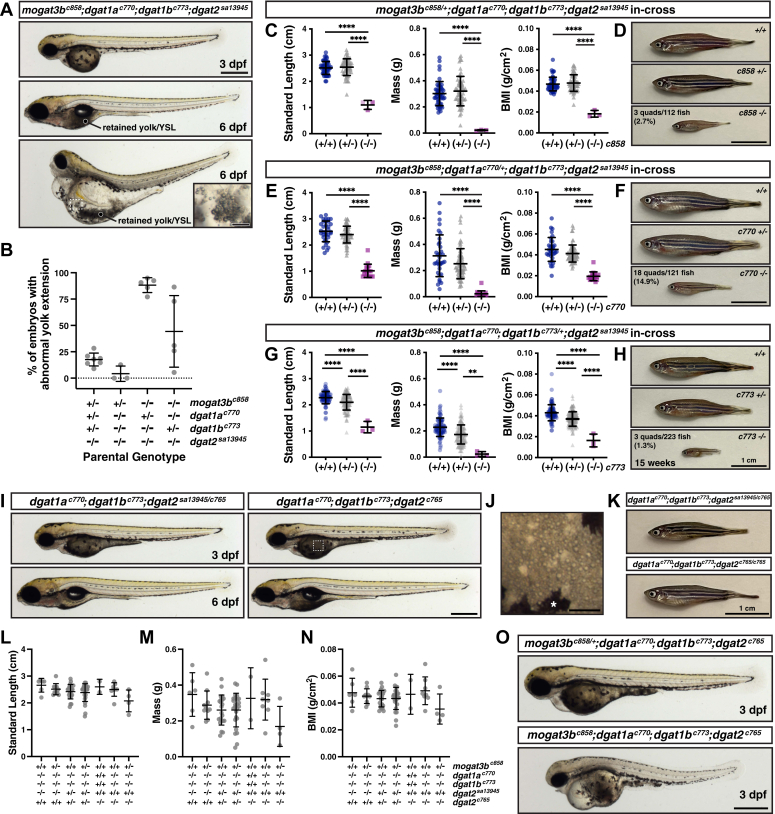
**Embryo health and survival is significantly affected by concurrent loss of four enzymes in the triacylglycerol synthesis pathway.***A*, representative images of embryos (3 dpf) and larvae (6 dpf) mutant for *mogat3b*^*c858*^*;dgat1b*^*c770*^*;dgat1b*^*c773*^*;dgat2*^*sa13945*^; Scale = 500 μm. The dashed *white* box in the bottom panel indicates the location of the inset; Scale = 100 μm. *B*, the percentage of embryos per clutch exhibiting abnormal yolk extension morphology (lack of YE, short YE, broken YE) on 3 dpf from in-crosses of the noted parental genotypes (N = 3–7 clutches per genotype (clutch size ranged from 24 to 600 embryos). *C–H*, standard length, mass and body mass index (BMI) data (*C, E, G*) for progeny from in-crosses of the noted parental genotypes at 14 weeks of age followed by genotyping. Graphs include data from both sexes; N = 2 to 3 clutches per genotype (n = 112 fish (*C*), n = 120 fish (*E*), n = 223 fish (*G*)); mean ± SD, One-way ANOVA, *p* < 0.0001 for all sets, Tukey’s multiple comparisons test ∗∗*p* < 0.01, ∗∗∗∗*p* < 0.0001. *D, F, H*, representative images of fish at 15 weeks of age; Scale = 1 cm. *I*, representative images of *dgat1b*^*c770*^*;dgat1b*^*c773*^*;dgat2*^*sa13945/c765*^ and *dgat1b*^*c770*^*;dgat1b*^*c773*^*;dgat2*^*c765*^ triple mutant fish at 3 and 6 dpf; Scale = 500 μm. The dashed white box indicates the location of the image in panel J. *J*, lipid droplets are still visible in the YSL of *dgat1b*^*c770*^*;dgat1b*^*c773*^*;dgat2*^*c765*^ mutants, ∗ melanocyte; Scale = 100 μm. *K*, representative images of *dgat1b*^*c770*^*;dgat1b*^*c773*^*;dgat2*^*sa13945/c765*^ and *dgat1b*^*c770*^*;dgat1b*^*c773*^*;dgat2*^*c765*^ triple mutant fish at 15 weeks of age; Scale = 1 cm. *L–N,* standard length, mass and body mass index (BMI) data at 14 weeks of age of progeny from in-crosses of *mogat3b*^*c858/+*^*;dgat1a*^*c770/+*^*;dgat1b*^*c773/+*^*;dgat2*^*sa13945/c765*^ and *mogat3b*^*c858/+*^*;dgat1a*^*c770/+*^*;dgat1b*^*c773/+*^*;dgat2*^*c765*^ or *mogat3b*^*c858/+*^*;dgat1a*^*c770*^*;dgat1b*^*c773*^*;dgat2*^*sa13945/*c765^ x *mogat3b*^*c858/+*^*;dgat1a*^*c770/+*^*;dgat1b*^*c773/+*^*;dgat2*^*c765*^ parents. Graphs include data from both sexes, for clarity only selected genotypes are shown; N = 16 clutches (n = 77 of the shown genotypes, 431 total fish); mean ± SD, One-way ANOVA for shown genotypes, *p* = 0.0722 (*L*), *p* = 0.0843 (*M*), *p* = 0.1665 (*N*). *O*, representative images of *mogat3b*^*c858/+*^*;dgat1a*^*c770*^*;dgat1b*^*c773*^*;dgat2*^*c765*^ and *mogat3b*^*c858*^*;dgat1a*^*c770*^*;dgat1b*^*c773*^*;dgat2*^*c765*^ quadruple mutants at 3 dpf; Scale = 500 μm.

Given the darker yolk opacity and abnormal yolk sac morphology in the *mogat3b;dgat2*^*sa13945*^ double mutants, we were curious whether zebrafish could survive in the absence of Mogat3b, Dgat1a, Dgat1b and Dgat2 enzymes. We initially crossed *mogat3b*^*c858*^*;dgat2*^*sa13945*^ fish to *dgat1a*^*c770*^*;dgat1b*^*c773*^*;dgat2*^*sa13945*^ fish to produce *mogat3b*^*c858/+*^*;dgat1a*^*c770/+;*^*dgat1b*^*c773/+*^*;dgat2*^*sa13945*^ fish and then generated quadruple mutants by in-crossing this stock. The quadruple mutant embryos are very similar to the *mogat3b*^*c858*^*;dgat2*^*sa13945*^ mutants in that they all lack yolk extensions and exhibit extensive yolk opacity (Fig. 8*A*, 3 dpf). By 6 dpf, some quadruple mutants can develop normally but exhibit obvious yolk/YSL retention (Fig. 8*A*, 6 dpf top panel, dark region over the intestine, compare to the wild-type fish in Fig. 1*E*). However, most quadruple mutants develop severe edema by 6 dpf (Fig. 8*A*, 6 dpf lower panel). In these fish, it is still possible to find remnants of YSL lipid droplets (Fig. 8*A*, 6 dpf lower panel, inset). After raising and genotyping 304 fish from four clutches, we have yet to identify an adult quadruple mutant from this crossing strategy, which is not surprising given that only one out of every 64 fish is expected to be a quadruple mutant.

However, from these crosses, we did find that *mogat3b*^*c858/+*^*;dgat1a*^*c770*^*;dgat1b*^*c773*^*;dgat2*^*sa13945*^, *mogat3b*^*c858*^*;dgat1a*^*c770/+*^*;dgat1b*^*c773*^*;dgat2*^*sa13945*^ and *mogat3b*^*c858*^*;dgat1a*^*c770*^*;dgat1b*^*c773/+*^*;dgat2*^*sa13945*^ fish do survive as adults and are fertile, which allowed us to perform in-crosses and raise clutches in which 25% of the fish are expected to be quadruple mutants. In these crosses, we noted differences in the proportion of progeny at 3 dpf exhibiting abnormal yolk extension morphology from parents with different genotypes. Specifically, those with crosses in which the parents were homozygous mutant for *mogat3*^*c858*^ were more likely to have large percentages of progeny with short or no yolk extension, with the *mogat3b*^*c858*^*;dgat1a*^*c770/+*^*;dgat1b*^*c773*^*;dgat2*^*sa13945*^ in-crosses especially affected (Fig. 8*B*). Further, we found that many of the quadruple mutant progeny from these crosses did not survive past 6 dpf, although the numbers varied widely between clutches (ranging from 16–84% of expected quadruple mutants). Despite this early mortality, we have now recovered small numbers of adult quadruple mutants from each of these genetic crosses. Notably, these fish are substantially smaller in length and mass than their siblings at 3 months of age (Fig. 8, *C*–*H*). The quadruple mutants remain small throughout their life (Fig. 8, *D, F and* H), and many exhibit early mortality by ∼1 year. All of the surviving fish appear to be male and at least one of these fish was fertile, although the clutch size recovered was very small.

Given that *dgat1a*^*c770*^*;dgat1b*^*c773*^*;dgat2*^*sa13945*^ triple mutants do not exhibit remarkable changes in body size (Fig. S10), this data argues that Mogat3b is important for zebrafish survival and growth, perhaps by supplying diacylglycerol to the remaining, still unidentified, triacylglycerol synthesis enzyme. This data also indicates that in the absence of *mogat3b*, even a single wild-type copy of either *dgat1a* or *dgat1b* is sufficient to promote normal growth, perhaps by also functioning as a monoacylglycerol acyltransferase (48). Notably, as embryos, the quadruple mutants still present with yolk opacity due to the accumulation of cytoplasmic lipid droplets, indicating that there must still be an enzyme producing neutral lipids and directing them toward aberrant storage in the YSL.

### Quadruple *mogat3b*^*c858*^*;dgat1a*^*c770*^*;dgat1b*^*c773*^*;dgat2*^*c765*^ mutants do not survive

As we were propagating our *dgat2* CRISPR/Cas9 mutants, we happened to notice that occasionally we would find homozygous *dgat2*^*c747*^ and *dgat2*^*c765*^ mutants that had short or absent yolk extensions, similar to what we had identified in the *mogat3b;dgat2*^*sa13945*^ double mutants (Fig. S16, *A and B*). This prompted further quantification of the phenotype of the *dgat2*^*c747*^ and *dgat2*^*c765*^ mutants in comparison to the *dgat2*^*sa13945*^ allele. When in-crossing homozygous mutants, we found that both of the CRISPR/Cas9 mutant alleles exhibit more yolk opacity than the *dgat2*^*sa13945*^ mutants, with the *dgat2*^*c765*^ allele exhibiting the strongest, most consistent phenotype (Fig. S16*C*). Additionally, the *dgat2*^*c765*^ mutants have significantly lower levels of ApoB-containing lipoproteins at 3 dpf when compared to *dgat2*^*sa13945*^ mutants, although the size of these lipoproteins is similar between mutant alleles (Fig. S16, *D and E*). These data suggest that the *dgat2*^*sa13945*^ allele may not be a true null allele. If there is some functional protein produced, perhaps due to read-through of the premature termination codon or use of an alternative start site, this would explain the lack of nonsense-mediated decay of the transcript, the variable penetrance and phenotype, and the milder phenotype compared to the *dgat2*^*c765*^ allele.

With this realization, we felt it was necessary to confirm some of the triple and quadruple mutant findings in fish expressing the stronger, *dgat2*^*c765*^ mutant allele. Thus, we crossed *dgat1a*^*c770/+*^*;dgat1b*^*c773/+*^*;dgat2*^*sa13945/c765*^ to *dgat1a*^*c770/+*^*;dgat1b*^*c773/+*^*;dgat2*^*c765*^ and generated *dgat1a*^*c770*^*;dgat1b*^*c773*^*;dgat2*^*sa13945/c765*^ and *dgat1a*^*c770*^*;dgat1b*^*c773*^*;dgat2*^*c765*^ triple mutants. While the triple mutants of both genotypes always exhibited the strongest yolk opacity phenotype at 3dpf (Fig 8*I*, 3 dpf; Fig. S17*A*), siblings with wild-type or heterozygous *dgat1* alleles were consistently more opaque if they carried two copies of the *c765* allele *versus sa13945/c765* compound heterozygotes (Fig. S17*A*). The yolk opacity in *dgat1a*^*c770*^*;dgat1b*^*c773*^*;dgat2*^*c765*^ triple mutants still appears to be due to aberrant accumulation of lipid droplets in the YSL (Fig. 8*J*). The percentage of embryos with abnormal yolk extension morphology at 3 dpf from these crosses was 5.9 ± 9.3% (N = 4 clutches). At 6 dpf, triple mutants with either the *dgat2*^*sa13945/c765*^ or *dgat2*^*c765*^ alleles were generally healthy although they tended to exhibit yolk retention (Fig. 8*I*, 6 dpf). Further, the triple mutants survive to adulthood and do not exhibit defects in growth compared to their siblings (Fig. 8*K*; Fig. S17, *B, C and D*).

To confirm whether any quadruple *mogat3b*^*c858*^*;dgat1a*^*c770*^*;dgat1b*^*c773*^*;dgat2*^*c765*^ mutants survive, we initially in-crossed *mogat3b*^*c858/+*^*;dgat1a*^*c770/+*^*;dgat1b*^*c773/+*^*;dgat2*^*sa13945/c765*^ or *mogat3b*^*c858/+*^*;dgat1a*^*c770/+*^*;dgat1b*^*c773/+*^*;dgat2*^*c765*^ parents. After raising and genotyping 431 fish, we have yet to identify an adult quadruple mutant containing the *dgat2*^*c765*^ allele, either as a compound *sa13945/c765* heterozygote or *c765/c765* homozygote. However, we did find healthy adult *mogat3b*^*c858/+*^*;dgat1a*^*c770*^*;dgat1b*^*c773*^*;dgat2*^*c765*^ mutants. These fish tend to be a bit smaller in size than their siblings (Fig. 8, *L*–N), but we have recovered both males and females and we have been able to successfully produce offspring from in-crosses of these fish. Notably, the health of these embryos is severely affected, with only 10 to 33% of the fish in each clutch surviving past 6 dpf (N = 4 clutches). At one dpf, the quadruple *mogat3b*^*c858*^*;dgat1a*^*c770*^*;dgat1b*^*c773*^*;dgat2*^*c765*^ embryos are not obviously different from their siblings (not shown), but by 2 to 3 dpf, they all exhibit pericardial edema, small eyes and imploded yolks (Fig. 8*O*). The quadruple mutants containing the *dgat2*^*c765*^ allele do not survive beyond 72 h post-fertilization. While many of the *mogat3b*^*+/+*^*;dgat1a*^*c770*^*;dgat1b*^*c773*^*;dgat2*^*c765*^ and *mogat3b*^*c858/+*^*;dgat1a*^*c770*^*;dgat1b*^*c773*^*;dgat2*^*c765*^ siblings are healthy at 6 dpf (Fig. 8*O*), by 6 dpf, these also often have extensive edema and are dying. Those that survive to 6 dpf have substantial yolk retention and by 14 weeks of age, only ∼70% of the fish raised were still alive. The majority (78%) of these fish are *mogat3b*^*+/+*^*;dgat1a*^*c770*^*;dgat1b*^*c773*^*;dgat2*^*c765*^ fish, while only 22% were *mogat3b*^*c858/+*^*;dgat1a*^*c770*^*;dgat1b*^*c773*^*;dgat2*^*c765*^ fish. Thus, while fish are able to survive as triple *dgat1a;dgat1b;dgat2* mutants, their survival is dependent on the presence of Mogat3b.

### Tmem68 is not responsible for the triacylglycerol synthesis in *dgat2* mutants

As our studies were ongoing, a novel diacylglycerol acyltransferase, TMEM68, was identified (65, 97). It is most highly expressed in the brain (98), and forms a complex with its regulatory partner, transmembrane thioredoxin 1 (TMX1) on the mitochondria-associated membrane of the ER (65). It exhibits both MGAT and DGAT activity (97), and is suggested to support mitochondrial function during periods of lipid starvation (65). In zebrafish, developmental expression atlas data indicates that *tmem68* is expressed at very low levels in larval stages (99), *in situ* hybridization data suggests low or no expression in the YSL (http://zfin.org), and we did not see an increase in *tmem68* expression in the *dgat2*^*sa13945*^ mutants in our RNA-seq data. However, to confirm that Tmem68 is not responsible for triacylglycerol synthesis in the YSL, we used a rapid CRISPR-based method to mutate the *tmem68* gene; this method has been shown to recapitulate germline-transmitted knockout phenotypes in >90% of injected embryos (100). We injected Cas9 protein + a pool of 4 CRISPR guide RNAs targeting the *tmem68* gene into the *dgat2* mutants or wild-type embryos at the 1-cell stage and assessed yolk phenotypes at 3 dpf. We hypothesized that if Tmem68 is responsible for the residual triacylglycerol synthesis, loss of gene function would prevent lipid droplet accumulation and associated yolk opacity. We chose to do these injections into the *dgat2*^*c765*^ mutants because they exhibit stronger and more consistent yolk opacity phenotypes than the *dgat2*^*sa13945*^ allele (Fig. S16), making interpretations of rescue easier. Despite strong evidence of gene disruption in the injected embryos, we did not observe any loss of yolk opacity (Figure S18, Supporting Information File 3). Thus, it is unlikely that Tmem68 is responsible for the lipid droplet-directed triacylglycerol synthesis in the YSL.

## Discussion

In this study, we have demonstrated that the loss of Dgat2 activity in the zebrafish yolk syncytial layer results in significant reductions in the size and quantity of ApoB-containing lipoproteins produced by the YSL. While these findings were expected based on data from cultured cells and rodents (44, 51, 60, 64, 101, 102, 103), the concomitant accumulation of cytoplasmic lipid droplets in the YSL was unexpected. Our data suggests that triacylglycerol synthesis still occurs in the absence of Dgat1 and Dgat2 enzymes, but that synthesis is not coupled properly to lipoprotein biogenesis in the ER. Notably, the residual TAG synthesis activity is sufficient to support the viability and health of adult *dgat2* and triple *dgat1a;dgat1b;dgat2* mutant fish.

During embryogenesis, the lipids stored in yolk platelets are lipolyzed and free fatty acids are released into the YSL cytoplasm where they can be used by the biosynthetic machinery at the ER to resynthesize phospholipids, triacylglycerol, and sterol esters. These lipids are packaged with ApoB to form lipoproteins and are secreted for circulation through the developing embryo. In wild-type fish, these processes are very well coupled, as lipid storage in cytoplasmic lipid droplets is only rarely observed (29, 30). The progressive accumulation of YSL lipid droplets in *dgat2* mutants and the concomitant increase in opacity suggests that yolk platelets continue to undergo degradation and triacylglycerol synthesis is still occurring, but the generated TAGs are not properly transferred to B-lps in the ER. How triacylglycerol synthesized within the ER bilayer is partitioned toward B-lps *versus* lipid droplets remains a mystery and our discovery of Dgat2-dependent neutral lipid channeling toward B-lp in the YSL is an important finding of this study.

The production of B-lps is thought to occur in two steps: MTP initially generates small primordial particles by transferring lipid to ApoB as it is translated/translocated into the ER lumen; subsequently, these particles are expanded, perhaps *via* fusion and/or transfer of TAG from ApoB-free lumenal lipid droplets (28, 104). In both the *dgat2*^*sa13945*^ and *dgat2*^*c765*^ mutants, total ApoB quantity is moderately reduced (∼25% and ∼40%, respectively), and the B-lps produced are markedly smaller in size and likely TAG-poor (Fig. 4, Fig. S16). This argues that Dgat2 is not essential for the initial production of nascent lipoprotein particles in the YSL, but is necessary for particle expansion to generate a mature, TAG-rich particle. These findings are consistent with data from primary mouse hepatocytes, in which DGAT2 inhibition led to secretion of higher density, TAG-poor lipoproteins, with no reduction in extracellular ApoB level (44). However, in contrast, antisense oligonucleotide-based knock-down of DGAT2 in mouse liver resulted in lower rates of both TAG and ApoB secretion, but particle size was unchanged (60). An additional study found that DGAT2 was responsible for particle number, but that DGAT1 activity determines particle expansion in the mouse liver (59). Discrepancies between studies are also noted when the activity of DGAT1 and DGAT2 are simultaneously affected. For instance, inhibition of both DGAT1 and DGAT2 in mouse or human hepatocytes, reduced ApoB secretion to a much greater extent than inhibition of either enzyme alone (44, 59), yet knockdown of DGAT2 in the liver of DGAT1 knockout mice reduced ApoB secretion to a similar extent as in wild-type mice, suggesting DGAT2 is the primary enzyme responsible for synthesizing TAG for secretion from the liver (60). In agreement with this latter study, our data indicate that zebrafish *dgat1a;dgat1b;dgat2* triple mutants have similar quantities of ApoB as *dgat2* mutant siblings, suggesting Dgat1 enzymes have minimal effect on ApoB secretion from the YSL in zebrafish (Fig. S10*C*). Thus, the specific roles of DGAT1 and DGAT2 in B-lp synthesis and expansion are still not entirely clear and may differ between tissues and between species.

While we do not know the composition of lipids in the secreted B-lp particles from *dgat2* mutants, we do provide evidence that the fish are still capable of synthesizing TAG and that the YSL cytoplasmic lipid droplets contain TAG. Since lipid droplets accumulate in *dgat1a;dgat1b;dgat2* triple mutants, we presume that these fish also continue synthesizing triacylglycerols. While this was initially surprising, a number of other studies have reported that cells and tissues have residual TAG production in the absence of both DGAT1 and DGAT2. For instance, macrophages cultured from the livers of E14 *Dgat1(−/−);Dgat2(−/−)* mice continue to synthesize TAG when incubated with acetylated LDL, albeit at lower levels than WT cells (105). More recently, data from adipose-specific double *Dgat1* and *Dgat2* knockout mice (ADGAT DKO), indicates that triacylglycerols continue to accumulate in white adipose tissue, although at levels ∼70% lower than controls (106). Further, these tissues maintained DGAT activity at ∼20%, which was not inhibited by DGAT1 and DGAT2-specific inhibitors, suggesting synthesis by an alternative enzyme(s) (106). While this alternative enzyme was not identified, it was noted that *Mgat1* and *Mgat2* mRNA were upregulated in ADGAT DKO white adipose tissue, suggesting perhaps these enzymes may be responsible. While we also hypothesized that Mogat2, Mogat3a or Mogat3b in the zebrafish might drive the residual TAG synthesis, these genes were not upregulated at the transcriptional level in *dgat2*^*sa13945*^ mutant embryos, overexpression of Mogat3b-FLAG did not rescue yolk opacity in *dgat2* mutants, and lipid droplet accumulation persists in *mogat3b;dgat1a;dgat1b;dgat2* quadruple mutants. Together, this suggests that Mogat enzymes are likely not responsible for the remaining TAG synthesis, however, until *mogat2* and *mogat3a* mutants are generated, we cannot completely rule out their involvement. We did not consider that acyl-CoA:cholesterol acyltransferases were responsible for TAG synthesis because the literature did not support such an activity (38, 107).

We made a number of additional attempts to identify the alternative pathway for triacylglycerol biosynthesis in *dgat* mutants. Using Atglistatin, we inhibited adipose triglyceride lipase, which can act as a diacylglycerol transacylase (82, 83, 84, 85), and did not find that it reduced YSL lipid droplet accumulation in *dgat2* mutants. We used a rapid CRISPR-based approach to mutate a recently reported, novel diacylglycerol acyltransferase gene, *tmem68* (65, 97), and again found no loss of YSL lipid droplets in *dgat2* mutants. RNA-seq analysis of WT *versus dgat2* mutants also did not reveal upregulation of any obvious candidate genes for TAG synthesis. Thus, despite our efforts to identify the enzyme responsible for TAG synthesis in the *dgat* mutant fish, we still do not have a candidate. However, the accumulation of YSL lipid droplets indicates that this unidentified enzyme is not properly coupling TAG production to B-lp secretion. This suggests that the enzyme is either not located in the proximity of MTP and ApoB on the ER membrane, or it does not properly interact/coordinate with the growing list of other proteins now implicated in lipoprotein lipidation (28). Although triacylglycerol molecules can diffuse in the bilayer (21), perhaps if they are too distant from the B-lp biosynthetic machinery, they may locally accumulate, phase separate prematurely, and bud outward into the cytoplasm, forming lipid droplets. In short, we cannot explain why ER-synthesized triacylglycerol is not accessible to the B-lp synthesis machinery in *dgat2* mutants.

The varied and incomplete penetrance of the mutant phenotype in the *dgat2*^*sa13945*^ allele was curious. This C > T point mutation should introduce a premature termination codon (PTC), which was expected to result in nonsense-mediated decay of the transcript. However, qPCR and mRNA *in situ* hybridization showed abundant *dgat2* mRNA in embryos. A closer look at the mutant sequence suggests that it is a good candidate for readthrough of the PTC, as the mutation introduces a UGA stop codon, which is more permissive to readthrough than UAA and UAG (108, 109). Moreover, the nucleotide immediately downstream of the stop codon (+4 position) also influences the likelihood of readthrough; this is a U in the *sa13945* allele (UAG**U**), which is the second-most readthrough-permissive base (110, 111). Evidence also suggests that there is a lot of cell-to-cell variability in levels of readthrough (112); thus, it is possible that these mutants may continue to produce full-length protein products and the severity of the phenotype could be inversely correlated to the amount of readthrough in an individual fish. Additionally, there is an in-frame AUG start codon six bases downstream of the PTC, which might allow for ribosome reinitiation, producing an N-terminally truncated protein product (113). This truncation would be expected to remove the N-terminal cytoplasmic domain of Dgat2 just prior to the putative transmembrane domain, but leave the presumed neutral lipid-binding domain and enzymatic motif intact (53). Unfortunately, we were not able to confirm the presence or absence of Dgat2 protein products in the mutants due to the lack of suitable antibodies, so it is unclear whether *dgat2*^*sa13945*^ mutants continue to produce a functional enzyme product.

The most remarkable finding of this study was the survival, health, and fertility of the adult *dgat2* mutants, and especially the *dgat1a;dgat1b;dgat2* triple mutant zebrafish. Unlike the fish, *Dgat2* and *Dgat1;Dgat2* double knockout mice die shortly after birth due to defective skin-barrier function (42, 51, 105). While this stark difference in survival could be due simply to differences in the lipid-requirements of the skin permeability barrier for a terrestrial *versus* aquatic species (50, 114, 115), we suspect that the differential survival is more than skin deep. For instance, the loss of either Dgat2 or both Dgat1 and Dgat2 enzymes in the mouse also severely depletes triacylglycerols from the whole body and liver (51, 105). In contrast, although we have not analyzed adult tissues for TAG content, we have shown that *dgat2* mutant zebrafish larvae continue to synthesize and store TAG in lipid droplets, and they do not show alterations in adipose tissue development as juveniles or body mass as adults. Further, we did not find significant changes in the body mass of *dgat1a;dgat1b;dgat2* triple mutants *versus* wild-type adult fish. This suggests that rodents and fish rely on distinct strategies for triacylglycerol synthesis. In support of this hypothesis, there are differences in the number of *DGAT1*/*MBOAT* and *DGAT2* gene family members present in different vertebrate species, as well as differences in tissue expression patterns of the various genes (41, 116).

In rodents, *Mogat1* encodes a protein expressed in the kidney, stomach, adipose tissue, and liver (117), but in humans, despite expression in many tissues, there is evidence of alternative splicing and transcripts are predicted to be non-coding and/or enzymatically inactive (32, 118, 119). *Mogat3* is a pseudogene in the mouse but is expressed in the intestine and pancreas in rats (95) and in the small intestine and liver in humans (32). However, human *MOGAT3* and murine *Mogat3* genes are not orthologs and may have evolved separately from duplication of the ancestral genes encoding *DGAT2* and *Mogat2*, respectively, in post-speciation duplication events (95). Following the teleost-specific whole genome duplication event, zebrafish retained two ohnologs of *dgat1*, and 4 *DGAT2* family members (*mogat2*, *mogat3a*, *mogat3b*, and *dgat2*). Similar to human MGAT3, the amino acid sequence of zebrafish Mogat3b is more similar to Dgat2 than to Mogat2 or Mogat3a, however, unlike human MGAT3 (32), our experimental data suggest that zebrafish Mogat3b does not possess meaningful diacylglycerol acyltransferase activity (Fig. 6).

Further, while *Dgat2* is expressed in the small intestine of the mouse, in humans its expression in intestinal epithelial stem cells is lost upon differentiation, such that it is only expressed at very low levels in the intestine (120, 121). Thus, patients with mutations in *DGAT1* experience congenital diarrhea and nutrient malabsorption (68, 120, 122, 123, 124), whereas Dgat2 can compensate for TAG synthesis in the intestine when Dgat1 is absent in the mouse (49, 58, 125). Species differences are also noted when using inhibitors; while inhibition of DGAT2 in rodents yields submaximal suppression of VLDL-TAG secretion from the liver, similar inhibition in rhesus monkeys did not affect plasma TAG or VLDL (103). These studies, together with the studies of TMEM68 (65, 97) and the transacylase activity of ATGL (82, 83, 84, 85), highlight the complexity of triacylglycerol synthesis pathways in and between vertebrates.

The differences in penetrance of yolk extension abnormalities and survival of quadruple mutant progeny from the various in-crosses of *mogat3b*^*c858/+*^*;dgat1a*^*c770*^*;dgat1b*^*c773*^*;dgat2*^*sa13945*^, *mogat3b*^*c858*^*;dgat1a*^*c770/+*^*;dgat1b*^*c773*^*;dgat2*^*sa13945*^ and *mogat3b*^*c858*^*;dgat1a*^*c770*^*;dgat1b*^*c773/+*^*;dgat2*^*sa13945*^ parents suggest having one wild-type parental copy of either *mogat3b*, *dgat1a* or *dgat1b* has differential effects on the progeny. For example, quadruple mutant progeny from parental fish that were heterozygous only for *dgat1a*^*c770*^ were more likely to survive to adulthood than those from fish that were only heterozygous at the *mogat3b*^*c858*^ or *dgat1b*^*c773*^ loci. The progeny from the *mogat3b*^*c858*^*;dgat1a*^*c770/+*^*;dgat1b*^*c773*^*;dgat2*^*sa13945*^ parents were also most likely to have abnormal yolk extension morphology. It is unclear whether these differences are due to differential maternal transcripts generated during oogenesis and deposited in the egg (126, 127), and/or changes in the composition of the maternally-deposited yolk lipids and associated lipid soluble vitamins (128) between the different maternal genotypes. Additionally, recent data suggests that metabolic changes in male fish can elicit paternal effects on the extra-embryonic tissues of progeny (129), so the differences we noted may result from both the male and female parents. Exploring yolk composition and the maternal transcriptome in embryos from these different genetic crosses may provide insights into the specific mechanisms driving the differences in yolk extension morphology and survival to adulthood.

A number of questions remain from our current study. For instance, once the yolk lipid is aberrantly packaged into cytoplasmic lipid droplets in the *dgat2* mutants, how is it metabolized and transported out of the yolk sac? While some fish retain giant lipid droplets (Fig. 1, *E* and *F*), most fish eventually clear these lipid droplets, suggesting that they are still able to export lipid from the YSL. This could be through lipase activity and resynthesis of the fatty acids as phospholipids or cholesterol esters for B-lp export. Or perhaps following lipolysis, the fish have found ways to export the free fatty acids directly into the circulation. Genetically targeting or chemically inhibiting candidate lipases, enzymes, and fatty acid transport proteins may address these possibilities. Following yolk platelet depletion and nutrient export, the YSL is degraded; the nuclei become pyknotic, the mitochondria and other organelles undergo autophagy and the YSL remnant may be cleared by phagocytes (69, 130). It is possible that the lipid droplets are eliminated during these processes, and a more detailed assessment of lipophagy and phagocytosis in larvae at 5 or 6 dpf could address this hypothesis.

In conclusion, loss of Dgat2 activity in the zebrafish leads to yolk sac opacity due to the accumulation of aberrant lipid droplets in the YSL. While this is associated with reduced production of triacylglycerol-rich ApoB-containing lipoproteins in embryos, this does not inhibit the development or the health of adult fish. Triple *dgat1a;dgat1b;dgat2* mutants exhibit similar phenotypes as *dgat2* mutants and it is only when *mogat3b*, *dgat1a*, *dgat1b* and *dgat2* are mutated that the fish are no longer viable (although as embryos they still exhibit YSL lipid droplets). These findings indicate that there is substantial redundancy between the genes required for viability and that there is an additional enzyme required for triacylglycerol synthesis. Given the importance of triacylglycerol as a fuel source and precursor for lipid membranes and signaling, perhaps the evolution and maintenance of multiple isoforms of mogats and dgats is an effective strategy to ensure continued triacylglycerol synthesis in different genetic and environmental contexts. Finally, these data also illustrate the power of the underappreciated zebrafish YSL for *in vivo* studies of both cytoplasmic lipid droplets and B-lp synthesis and metabolism.

## Experimental procedures

### Zebrafish husbandry and strains

All Zebrafish (*Danio rerio*) protocols were approved by the Carnegie Institution Department of Embryology Animal Care and Use Committee (Protocol #139) or Duke University Medical Center Institutional Animal Care and Use Committee (Protocol registry A115–16–05). Zebrafish stocks were maintained at 27°C in a circulating aquarium facility with a 14:10 h light:dark cycle. Fish were housed in tanks with a maximum density of 10 fish/L. Embryos were obtained by natural spawning and raised in embryo medium at 28.5°C in culture dishes in an incubator with a 14:10 h light:dark cycle. All embryos used for experiments were obtained from pair-wise crosses and the developmental stage was assessed according to (131). Exogenous food was provided starting at 5.5 days post fertilization (dpf) unless otherwise noted. Larvae were fed with GEMMA Micro 75 3x daily until 14 dpf, GEMMA Micro 150 3x daily + Artemia 1x daily from 15 to 42 dpf and then ∼3.5% body weight GEMMA Micro 500 1x daily supplemented once a week with Artemia. The nutritional content of GEMMA Micro (Skretting) is: Protein 59%; Lipids 14%; Fiber 0.2%; Ash 14%; Phosphorus 1.3%; Calcium 1.5%; Sodium 0.7%; Vitamin A 23,000 IU/kg; Vitamin D3 2800 IU/kg; Vitamin C 1000 mg/kg; Vitamin E 400 mg/kg. Sex of adult fish included in analyses is noted in figure legends; however, zebrafish sex is not determined until juvenile stages so is not available for experiments with embryos and larvae.

*Dgat2*^*sa13945*^ mutant zebrafish were produced by ENU-based mutagenesis as part of the Wellcome Sanger Institute Zebrafish Mutation Project (31). Embryos were received at Carnegie on the Hubrecht long-fin background and were subsequently crossed into the wild-type AB strain. The *dgat2*^*sa13945*^ allele was crossed into the *Fus(ApoBb.1-Nanoluc)* LipoGlo reporter *c832 Tg* line (11), the *Fus(EGFP-plin2) c874 Tg* line (72), or ER stress 5xATF6RE:d2GFP *mw85 Tg* reporter line (71). All additional mutants were generated in the AB or *dgat2*^*sa13945*^ strain as described below.

### Sequence, syntenic, and phylogenetic analyses

Syntenic relationships between zebrafish and human *mogat* genes were explored using the Ensembl Genome Browser (Ensembl.org, (EMBL-EBI)) (132) and the Genomicus online syntenic analysis tool (133). Amino acid sequences of human (*Homo sapien*), mouse (*Mus musculus*), rat (*Rattus norvegicus*), opossum (*Mondelphis domestica*), tropical clawed frog (*Xenopus tropicalis*), spotted gar (*Lepisosteus oculatus*), tetraodon (*Tetraodon nigroviridis*), and zebrafish (*D. rerio*) DGAT2 and MOGAT family members were obtained from Ensembl.org. Optimal pair-wise alignments between human or zebrafish ortholog protein sequences were performed with MacVector V15.5 (MacVector, Inc.), using the T-coffee progressive alignment algorithm. To examine the phylogenetic relationship between zebrafish paralogs and their corresponding vertebrate orthologs, the amino acid sequences of human, mouse, rat, opossum, *xenopus*, spotted gar, tetraodon, and zebrafish Dgat2 and Mogat proteins were aligned using the MAFFT multiple sequence alignment tool (134). The G-INS-I strategy was selected with standard settings; following alignment, the MAFFT tree server was used to build a neighbor-joining (NJ) phylogenetic tree based on all gap-free sites, using the JTT substitution model and allowing estimation of site heterogeneity (alpha). Bootstrap resampling was set to 100, and bootstrap support >70 was interpreted as significant. Accession numbers of the zebrafish and other species genes and transcripts used in this analysis are listed in Supporting Information File 1.

### CRISPR/Cas9 mutant generation

Mutant lines for *dgat2* (GRCz11: ENSDARG00000018846), *dgat1a* (GRCz11: ENSDARG00000103503) and *dgat1b* (GRCz11: ENSDARG00000054914), and *mogat3b* (GRCz11: ENSDARG00000003635) were generated using CRISPR/Cas9. sgRNAs were designed according to (135) and synthesized as described in (136). *Dgat2*^*c747*^ was generated with CRISPRs targeting exon 2 (dgat2-1 5′- GGATCGGGTCAGCCACGGGA-3′ and dgat2-2 5′- GGTGAGTGGACGAGTGACGG-3′) in AB fish. *Dgat2*^*c765*^ was generated with CRISPRs targeting exon 5 (dgat2-3 5′- GGTAGCCAAAAATGTAGTTT-3′ and dgat2-4 5′- GGATTCACCAAGGTGTTCCC-3′) in AB fish. *Dgat1a*^*c770*^ was generated with CRISPRs targeting exon 1 (dgat1a-1 5′-GGGCGGAGGACTACGATCTC-3′ and dgat1a-2 5′-GGGACTCAAGCCAAACGCGG-3′) in *dgat2*^*sa13945*^ fish. *Dgat1b*^*c773*^ was generated with CRISPRs targeting exon 1 (dgat1b-1 5′-GGGCGACCATCACGAGTACC-3′ and dgat1b-2 5′-GGTGCGAAACGGCGCGAGCG-3′) in *dgat2*^*sa13945*^ fish. *Mogat3b*^*c858*^ and *mogat3b*^*c862*^ were generated with CRISPRs targeting exon 4 & 5 (mogat3b-1 5′-GGGAGTCTTGTTACCTTCAC-3′ and mogat3b-2 5′-GGGAGGAAGGCGGAAAAGT-3′) in *dgat2*^*sa13945*^ fish. For guide sequences, see Supporting Information File 1.

sgRNA primer oligonucleotides 5′-TAATACGACTCACTATA-GGN(_18-20_)-GTTTTAGAGCTAGAAATAGC-3′ and universal bottom strand ultramer oligonucleotide 5′- AAAAGCACCGACTCGGTGCCACTTTTTCAAGTTGATAACGGACTAGCCTTATTTTAACTTGCTATTTCTAGCTCTAAAAC-3′ were synthesized by Eurofins Genomics. Oligonucleotides were annealed and filled in using Phusion polymerase (M0530S, New England BioLabs) with the following protocol: 98°C 30 s, (98°C 10 s, 55°C 30 s, 72°C 30 s) x 15, hold at 15°C. Following purification with the QIAQuick PCR purification kit (28,104, Qiagen), the MegaShort script T7 kit (AM1354, ThermoFisher Scientific) was used to make sgRNA. All sgRNAs were DNase treated, precipitated with ammonium acetate/ethanol, and were suspended in nuclease-free H2O. Zebrafish embryos were injected at the one-cell stage with 300 pg total guide RNA (150 pg each of two guides co-injected) + 500 pg Cas9 protein (EnGen Spy Cas9 NLS, M0646 T, 20 μM, New England BioLabs) in an injection mix containing 1 U/μl SUPERase In RNase inhibitor (AM2694, Invitrogen) and 0.05% phenol red.

F0 injected adults were out-crossed to wild-type AB fish and progeny were screened for deletions by PCR using the primers sets described below. Clutches carrying promising candidates were raised and the F1 generation was genotyped as adults by fin clip. Deletions and/or insertions in the gDNA were verified by Sanger sequencing (Genewiz). Expected changes based on the gDNA were confirmed by Sanger sequencing cDNA made from homozygous mutant embryos in the F2 generation (details below).

To generate *tmem68* F0 CRISPANTs, the *tmem68* gene locus in AB and *dgat2*^*c765*^ mutants were targeted using the rapid CRISPR-based method described in (100). Zebrafish embryos were injected at the one-cell stage with 800 pg Cas9 protein + 1000 pg of a pool of either 4 CRISPR guide RNAs targeting the *tmem68* gene (5′-GGCCACACATGGACCAGGCA-3′, 5′-GGTTTTCACACCGCTAGTCG-3′, 5′-GGATGGAGCAAGGAAAACTC-3′, 5′-GGTCATAGTCTATTATCACG-3′) or with a control pool of four random sequence scrambled guides (5′-GGCAGGCAAAGAATCCCTGCC-3′, 5′-GGTACAGTGGACCTCGGTGTC-3′, 5′-GGCTTCATACAATAGACGATG-3′, 5′-GGTCGTTTTGCAGTAGGATCG-3′). The sequences for the four-guide sgRNA sets were obtained from the lookup table provided by (100) and synthesized as described above, except that the fill-in PCR reactions for all four guides were combined prior to purification and sgRNA synthesis. Embryos were imaged and scored at 3 dpf for phenotype, followed by extraction of gDNA and PCR-based confirmation of indels in exon two of the *tmem68* locus as described below.

### DNA extraction and genotyping

Genomic DNA was extracted from embryos, larvae, or adult fin clips using a modified version of the HotSHOT DNA extraction protocol (137). Embryos or tissue was heated to 95°C for 18 min in 100 μl of 50 mM NaOH, cooled to 25°C and neutralized with 10 μl of 1 M Tris-HCl pH 8.0. Genotyping primers for the *dgat2*^*sa13945*^ allele was designed using the dCAPS Finder 2.0 program (138) and synthesized by Eurofins Genomics. The *dgat2*^*sa13945*^ locus was amplified using the forward primer 5′-TAC ACG ACC TGC CCA CCG TCC CGT GGC GGA TC-3′ and reverse primer 5′-ACC GCT GAA ATC AAG TGG AG-3’ (191 bp; 0.5 μM primer, T_a_ = 55°C, extension time 60 s). The forward primer introduces a BamHI restriction site only into the wild-type amplicon and digestion with BamHI-HF (0.3 μl (6 units) per 20 μl reaction (R3136 L, New England BioLabs) at 37°C for 75 to 90 min yields wild-type bands at 28 bp +163 bp *versus* the mutant band at 191 bp. The *dgat2*^*c747*^ locus was amplified using the forward primer 5′- ACG TGG TTC CTG ATT AAG CCT-3′ and the reverse primer 5′-ACC GCT GAA ATC AAG TGG AG-3’ (wild-type allele: 465 bp, *c747* allele: 352 bp; 0.5 μM primer, T_a_ = 55°C, extension time 60 s). The *dgat2*^*c765*^ locus was amplified using the forward primer 5′-GTG AAG AGT GAA TAA ACG AGG-3′ and reverse primer 5′-GCA ACT GCA GGC AAT GAA TAC-3’ (wild-type allele: 455 bp, *c765* allele: 365 bp; 0.5 μM primer, T_a_ = 61°C, extension time 60 s). The *dgat1a*^*c770*^ locus was amplified using the forward primer 5′-ATA TGG AAA CAG CAT GAG AG-3′ and reverse primer 5′-AAT GTG TCC TCT CCA GCA TC-3’ (wild-type allele: 240 bp, *c770:* 226 bp; 0.5 μM primer, T_a_ = 59°C, extension time 30 s; 2% gel; the *c770* mutant and wild-type alleles form a heteroduplex resulting in three bands in the heterozygous fish, see Fig. S9*B*). The *dgat1b*^*c773*^ locus was amplified using the forward primer 5′-CCA AAT GAG ACT CCT GCC TG-3′ and reverse primer 5′-TCA TTC TGT CGT AGC GGT CG-3’ (wild-type allele: 297 bp, *c773:* 244 bp; 0.25 μM primer, T_a_ = 59°C, extension time 30 s). The *mogat3b*^*c858*^ and *mogat3b*^*c862*^ locus was amplified using the forward primer 5′- TGA GAG GCT GGG AAG TAT GGA-3′ and the reverse primer 5′- TGA GTC TCA CTG CAG CTA ATT TC-3’ (wild-type allele: 374 bp, *c858:* 98 bp, *c862:* 412 bp; 0.5 μM primer, T_a_ = 59°C, extension time 40 s). For the Fus(*apoBb.1-NanoLuc) c832 Tg* genotyping protocol, see (11). To confirm the presence of the *Fus(EGFP-plin2) c874 Tg* or 5xATF6RE:d2GFP *mw85 Tg* alleles, a region of the GFP coding sequence was amplified using the forward primer 5′- GGT GAA CTT CAA GAT CCG CCA-3′ and the reverse primer 5′-GAA CTC CAG CAG GAC CAT GT-3’ (184 bp; 0.5 μM primer, T_a_ = 59°C, extension time 30 s). For *tmem68* F0 CRISPANTs, the region across exon 2 targeted by 3 of the 4 CRISPR sgRNAs was amplified using the forward primer 5′-TTG GCA GAT TGT TGC CTT GC-3′ and reverse primer 5′-TGC TTT GGT CAT TCC TGT TCA T-3’ (wild-type allele: 507 bp; 0.5 μM primer, T_a_ = 65°C, extension time 1 min). PCR amplicons were run on one or 2% agarose gels in TBE and gels were imaged with a Bio-Rad Gel ChemiDoc XRS system and Quantity One software. For primer information, see Supporting Information File 1.

Additional confirmation of deleterious indels in the *tmem68* F0 CRISPANTs was performed *via* amplicon sequencing. A smaller region across exon two was amplified using the forward primer 5′-TTG GCA GAT TGT TGC CTT GC-3′ and the reverse primer 5′-CCA TTA CAT ACC ATG CCA AAT AGC-3’ (wild type allele: 400 bp; 0.5 μM primer, T_a_ = 65°C, extension time 1 min). PCR products from embryos injected with the *tmem68* guide RNA (n = 8) or scramble guide RNA (n = 8) shown in Fig. S18*B* were mixed and purified using the QIAQuick PCR purification kit (28,104, Qiagen). Amplicons were sequenced and analyzed with the Genewiz Amplicon-EZ next-generation sequencing and analysis pipeline (Azenta Life Sciences). Reads were analyzed against a target region beginning at the start codon located in exon 2 through to the end of the PCR amplicon (315 bp).

### Brightfield microscopy and morphometrics

Embryos and larvae were mounted in 3% methylcellulose (Sigma, M0387) in embryo media on glass slides for whole-mount imaging. Bright-field images were obtained using a Nikon SMZ1500 microscope with HR Plan Apo 1x WD 54 objective, Infinity 3 Lumenera camera, and Infinity Analyze 6.5 software. If needed, image contrast was uniformly adjusted in Fiji (ImageJ V2.1.0, National Institutes of Health, Bethesda, MD; (139)). Standard length (140), muscle area and yolk sac area were measured using Fiji. Yolk opacity at 3 dpf was scored in a blinded manner, binning fish into the following four categories: translucent (normal), opaque yolk extension, opaque patches in the anterior yolk, and opaque across the whole yolk. Yolk extension morphology was assessed at 3 dpf, either from bright-field images of embryos, or while viewing live under a dissecting microscope; the yolk extension was considered to be abnormal if absent, shortened, or exhibiting constrictions. For adult morphometrics, fish were anesthetized with tricaine (Sigma-Aldrich A5040), the mass of fish at 3 months (14 weeks) or 6 months was obtained with a Sartorius CP423 S balance, and standard length was measured with a ruler; body mass index was subsequently calculated (g/cm^2^) for each fish. Fin clips were obtained at the time of measurement for genotyping. Images of adult zebrafish were obtained at 15 weeks with an iPhone 13 mini mounted at a fixed distance from the imaging surface with a ruler in the field of view, 3x zoom.

### Whole-mount *in situ* hybridization

Zebrafish embryos and larvae were staged according to Kimmel *et al.* (131) and fixed with 4% paraformaldehyde in phosphate-buffered saline solution overnight at 4°C, washed twice with MeOH and stored in MeOH at −20°C. To generate riboprobes, ∼450 to 850 bp of unique coding sequence was amplified from cDNA of wild-type fish for *dgat2*, *dgat1a*, *dgat1b*, *mogat3a*, *mogat2*, and *mogat3b* using the primers noted in Supporting Information File 1 and cloned into the pCRII-TOPO vector (Invitrogen, K461020). Sense and anti-sense probes were synthesized with the DIG RNA labeling kit (Roche, 11,277,073,910) using T7 and SP6 polymerases (Roche, 10,881,767,001 and 10,810,274,001). *In situ* hybridization at three and 6 dpf was performed as previously described (141). Experiments were performed 3 times on ≥ 5 embryos from independent clutches for each sense and anti-sense probe at each stage. For imaging, embryos/larvae were mounted in glycerol and imaged using the Nikon SMZ1500 microscope described above with incident light. For cross-section images, embryos were embedded in O.C.T compound (Sakura Finetek, 4583) in Tissue-Tek Cryomolds (4565, Sakura Finetek USA Inc), frozen on dry ice, and 10 μm thick sections were cut on a Leica CM3050 S cryostat with a microtome blade (VWR 95057–834) and mounted on Superfrost Plus microscope slides (12–550–15, Thermo Fisher Scientific). Slides were washed with 1x phosphate-buffered saline and sections were mounted with coverslips in 100% glycerol. Images were obtained with a Nikon E800 microscope with a Plan Apo Nikon 20x/0.75 DIC M objective and Canon EOS T3 camera using EOS Utility image acquisition software. If needed, image contrast was uniformly adjusted in Fiji (ImageJ V2.1.0, National Institutes of Health [NIH]; (139)).

### Oil Red O staining

Zebrafish embryos and larvae at 3 to 6 dpf were fixed with 4% paraformaldehyde in PBS for 3 h at room temperature and then overnight at 4°C. Fish were rinsed in 60% 2-propanol for 10 min rocking, and then put into 0.3% Oil Red O (Sigma-Aldrich, #O0625) in 60% 2-propanol to rock overnight at room temperature. Fish were rinsed 3 × 15 min with 60% 2-propanol and equilibrated step-wise into glycerol. Fish were imaged in glycerol with incident light using a Nikon SMZ1500 microscope with HR Plan Apo 1x WD 54 objective, Infinity 3 Lumenera camera, and Infinity Analyze 6.5 software. If needed, image contrast was uniformly adjusted in Fiji (ImageJ V2.1.0, National Institutes of Health [NIH; (139)).

### RNA isolation, cDNA synthesis, and quantitative RT-PCR

RNA was extracted from whole embryos individually using the Machery-Nagel NucleoSpin RNA XS with no carrier RNA (740,902.50, Takara Bio USA Inc) or in groups using the Zymo Direct-zol RNA MicroPrep R2060 kit, Zymo. cDNA was synthesized using the iScript cDNA Synthesis Kit (1,708,891, Bio-Rad Laboratories, Inc.). cDNA was used for PCR amplification of specific loci, qRT-PCR, or both. For adult expression analyses, age-matched 2-year old male *dgat2*^*sa13945/+*^ and *dgat2*^*sa13945*^, or *dgat2*^*c765/+*^ and *dgat2*^*c765*^ fish were fasted for 24 h, anesthetized, and then euthanized by submersion in ice-cold water. Liver and anterior intestine tissue was dissected and stored in NucleoProtect RNA solution (Machery-Nagel, 740,400) at 4°C for one or 2 weeks prior to RNA extraction. Tissues were moved into buffer RA1 + TCEP from the Machery-Nagel NucleoSpin RNA XS kit (740,902.50, Takara Bio USA Inc) and homogenized in a BulletBlender (Next Advance, 5E; 0.5 mm zirconium oxide beads Zr0B05, setting six for 1 min). RNA was extracted and cDNA was synthesized as described above.

See Supporting Information File 1 for primers used to amplify and sequence specific mutant loci in the cDNA. The *dgat2*^*sa13945*^ and *dgat2*^*c747*^ loci were amplified using the forward primer 5′-GGA ACC TTA GAT CTG CAA ACA TGA AGA CCA TAC TTG CTG C-3′ and reverse primer 5′-AGT CTC TCA CCC AGG TCG AT-3’ (WT & *sa13945* 322 bp, *c747* 191 bp; 0.5 μM primer, T_a_ = 59°C, extension time 60 s). The *dgat2*^*c765*^ locus was amplified using the forward primer 5′-TTA CCA TGG GCA TTG CTT GCT-3′ and reverse primer 5′-CAG ATT CCT CCG CAC ATC AGG-3’ (WT 400 bp, *c765* 310 bp; 0.5 μM primer, T_a_ = 54°C, extension time 60 s). The *dgat1a*^*c770*^ locus was amplified using the forward primer 5′-CGG CTA TAT GGA AAC AGC ATG AG-3′ and reverse primer 5′-CTC AGA GAT GGT GCC CAC AG-3’ (WT 545 bp, *c770* 531 bp; 0.5 μM primer, Touchdown PCR starting at T_a_ = 66°C with a decrease of 0.5°C/cycle for 29 cycles + 15 cycles with T_a_ = 51°C, extension time 60 s). The *dgat1b*^*c773*^ locus was amplified using the forward primer 5′-AAG CTT GTT TTT CCG GTC GC-3′ and reverse primer 5′-CTG CAA CTT GTG GCA ACT CA-3’ (WT 338 bp, *c773* 285 bp; 0.5 μM primer, T_a_ = 53°C, extension time 60 s). The *mogat3b*^*c858*^ and *mogat3b*^*c862*^ loci were amplified using the forward primer 5′-GAT TGG CAC ACA CCT GAG AGA-3′ and reverse primer 5′-AGC GTT ACC CAC ACC AGT TT-3’ (WT 363 bp, *c858* 87 & 175 bp, c862 401 bp; 0.5 μM primer, T_a_ = 64°C, extension time 60 s). Due to the alternative splicing of the *mogat3b*^*c858*^ mRNA and the production of two transcripts, it was necessary to clone the PCR products or perform Band-stab-PCR (142) in order to obtain sequences for each transcript. Following TOPO TA cloning (Invitrogen, K4560), DH5⍺ competent cells (Invitrogen, 18,265,017) were transformed and spread on LB-agar plates with 100 μg/ml carbenicillin along with 40 mg/ml X-gal and incubated at 37°C overnight. Colony PCR was performed using the forward primer 5′-GAT TGG CAC ACA CCT GAG AGA-3′ and reverse primer 5′-AGC GTT ACC CAC ACC AGT TT-3’ (0.25 μM primer, T_a_ = 64°C, extension time 60 s); only the larger 175 bp product was recovered and confirmed with Sanger sequencing using both the forward and reverse primers. Due to the small size of the 87 bp band, we added partial Illumina adaptor sequences to the original primers (forward: 5′-ACA CTC TTT CCC TAC ACG ACG CTC TTC CGA TCT GAT TGG CAC ACA CCT GAG AGA-3’ & reverse: 5′-GAC TGG AGT TCA GAC GTG TGC TCT TCC GAT CTA GCG TTA CCC ACA CCA GTT T-3′) and performed stab-PCR on the initial 87 bp product, increasing the amplicon to 152 bp (0.5 μM primer, T_a_ = 72°C, annealing + extension time 60 s). The resulting amplicon was then sequenced using the following adaptor sequence primers (forward: 5′- ACA CTC TTT CCC TAC ACG ACG CTC TTC CGA TCT-3’ & reverse: 5′-GAC TGG AGT TCA GAC GTG TGC TCT TCC GAT CT-3′).

qRT-PCR samples were prepared using SsoAdvanced Universal SYBR Green Supermix (1,725,271, Bio-Rad Laboratories, Inc.), and primers designed against gene-specific sequences (See Supporting Information File 1); zebrafish 18S (*rps18*) was used as the reference gene (9). Primers were validated for a single amplicon at the expected size on a 1% agarose gel stained with ethidium bromide. qRT-PCR was performed in triplicate for each sample with the Bio-Rad CFX96 or Bio-Rad CFX Opus 384 Real-Time System: 95°C for 30 s, followed by 40 cycles of 95°C for 10 s 60°C for 30 s, with the read at 60°C. Results were analyzed with the Bio-Rad CFX Manager 3.0 software and relative gene expression was calculated using the ΔΔCT method (143).

### Rescue of *dgat2*^*sa13945*^ opaque yolk phenotype

CMV:*dgat2*-FLAG was synthesized using the Tol2kit Gateway cloning system in the pDEST Tol2-pA2 backbone using the p5E-CMV/SP6 and p3E-polyA entry clones (144), along with a pME-zdgat2-CDS-FLAG middle entry vector containing the zebrafish *dgat2* coding sequence with a C-terminal FLAG tag (generated in pDONR-221 backbone). CMV:EGFP-CAAX was synthesized using the Tol2kit Gateway cloning system using the p5E-CMV/SP6, pME-EGFP-CAAX, and p3E-polyA entry clones (144). The wild-type zebrafish *dgat1a*, *dgat1b* and *mogat3b* coding sequences with FLAG-tags prior to the termination codon at the C-termini were generated by custom gene synthesis and cloned into the pcDNA3.1+ vector to generate CMV:dgat1a-FLAG, CMV:dgat1b-FLAG and CMV:mogat3b-FLAG rescue plasmids (Gene Universal Inc., Newark, DE).

*Dgat2*^*sa13945*^ embryos were injected at the 1-cell stage with 20 pg of rescue plasmids and 20 pg of CMV:EGFP-CAAX as a marker of successful injections. Embryos were raised to 3 dpf and screened for EGFP expression in the yolk sac. Bright-field images of the EGFP+ control and EGFP+ CMV:*dgat2*-FLAG, CMV:dgat1a-FLAG+ CMV:dgat1b-FLAG, or CMV:mogat3b-FLAG injected embryos were obtained, blinded and scored for yolk opacity as described above.

### Transmission electron microscopy

Wild-type, *dgat2*^*sa13945*^ and *dgat1a*^*c770*^*;dgat1b*^*c773*^*;dgat2*^*sa13945*^ triple mutant zebrafish embryos were fixed at 4 dpf in 3% glutaraldehyde, 1% formaldehyde, 0.1 M cacodylate solution for 1 to 3 h. Embryos were trimmed and swim bladders were deflated. Embryos were embedded in 2% low melt agarose and processed as described in (145). Post-fixation was performed for 1 h with 1% osmium tetroxide and 1.25% potassium ferricyanide in cacodylate solution. After 2 × 10 min washes with H_2_O, samples were incubated with 0.05 M maleate pH 6.5 for 10 min. Samples were stained *en bloc* with 0.5% uranyl acetate in maleate at 4°C overnight. After 2 × 15 min washes with H_2_O, samples were dehydrated through graded EtOH solutions (35%, 2 × 15 min; 50%, 15 min; 75%, 15 min, 95%, 15 min; 100% 4 × 15 min). Samples were washed with propylene oxide 4 × 15 min before incubation with 1:1 propylene oxide/resin (Epon 812 epoxy, Ladd Research Industries, Williston, VT) for 1 h and evaporated overnight. Samples were washed 2 × 1 h with 100% resin and embedded finally in 100% resin at 55°C overnight followed by 70°C for 3 days. Sections were cut on a Reichert Ultracut-S (Leica Microsystems), mounted on naked 200 thin mesh grids, and stained with lead citrate. Images were obtained with a Phillips Technai-12 electron microscope (FEI, Hillsboro, OR) and 794 Gatan multiscan CCD camera (Gatan, Pleasanton, CA) using Digital Micrograph software or with a Hitachi HT7800 electron microscope and AMT Nanosprint 12 camera. ER width was quantified with Fiji (ImageJ V2.1.0, National Institutes of Health [NIH], Bethesda, MD; (139)).

### *Fus(EGFP-plin2)* and confocal imaging

*Dgat2*^*sa13945*^ mutants were crossed to the *Fus(EGFP-plin2)*^*c874*^ knock-in line (72) and then subsequently in-crossed. For confocal imaging of live larvae, *dgat2*^*sa13945*^ mutants and wild-type siblings (3 dpf) were anesthetized with tricaine and mounted in 3% methylcellulose on glass slides with bridged coverslips. Images were obtained with a Leica DMI6000 inverted microscope and Leica 63 × /1.4 HCX PL Apo oil-immersion objective with a Leica TCS-SP5 II confocal scanner with photomultiplier detectors using Leica Application Suite Advanced Fluorescence 2.7.3.9723 image acquisition software. Images were obtained using four-line averaging, bidirectional scanning, 2x zoom, and recorded with 12-bit dynamic range. EGFP was excited with an argon laser (488 nm) and had a collection window of 498 to 530 nm. Image contrast for brightfield and EGFP channels were adjusted equally for all images using Fiji (ImageJ V2.1.0, National Institutes of Health [NIH], Bethesda, MD; (139)). Following imaging, embryos were collected for genotyping of both *dgat2*^*sa13945*^ and *EGFP* as described above.

### TopFluor C11 injections and thin-layer chromatography

Embryos at 3 dpf were mounted in 1.2% low-melt agarose (Fisher Scientific, BP165) in embryo media on clear plastic plates. A working stock of 0.4 mg/ml TopFluor C11 (custom synthesis, Avanti Polar Lipids) in canola oil (Giant Food, store brand) was prepared and loaded into microinjection needles. Each embryo was injected with 2 nl of the TopFluor lipid solution directly into the yolk mass. Embryos were freed from the agarose with forceps and incubated for 4 h at 28.5°C prior to collection. Twenty embryos per sample were collected in a microcentrifuge tube, media was eliminated and samples were frozen on dry ice and stored at −80°C until analysis. Frozen embryos were sonicated in 200 μl of homogenization buffer (20 mM Tris-HCl, 1 mM EDTA). Lipids were extracted using a modified Bligh-Dyer procedure (146). Following the addition of 750 μl of 1:2 CHCl3:MeOH, samples were vortexed for 30 s, and incubated at room temperature for 10 min. Two-hundred 50 μl of chloroform was added, samples were vortexed for 30 s, 250 μl of homogenization buffer was added, and samples were vortexed for 30 s and spun at 2000 x g for 5 min. The bottom layer of chloroform was recovered (∼500 μl) and moved to a new microcentrifuge tube. Samples were dried under vacuum for 20 min, resuspended in 40 μl of 2:1 CHCl3:MeOH by vortexing and loaded onto a thin layer chromatography plate (product info 43911). The tube was rinsed with 30 μl of CHCl3 and vortexed and this volume was loaded onto the same lane. TopFluor-labeled standards for TAG, CE, PC, DG, MG and C11 (TopFluor TAG: 810298; TopFluor CE: 810290; TopFluor PC: 810,281 TopFluor DG: 810300; MG: 810296; TopFluor C11 custom synthesis, Avanti Polar Lipids) were added to lipids extracted from uninjected embryos and loaded similarly onto the TLC plate. Plates were run initially in 22:28:0.5 Hexane:diethyl ether: acetic acid to ∼75% up the plate, allowed to dry and then run in in 16.25:6.25:1 CHCl3:MeOH:H2O to ∼80% of the stacker to concentrate the phospholipids. Plates were imaged with a Typhoon 9410 variable mode imager (Cytiva) at 350PMT. Fluorescent lipids were quantified using ImageQuant software and expressed as a percent of total lipids.

### Lipid droplet dissections and HPLC

*dgat2*^*sa13945*^ larvae at 7 dpf were anesthetized and mounted individually in droplets of embryo media containing tricaine on clear plastic plates on the dissecting microscope stage. Visible large lipid droplets were manually dissected from the bodies using 28 gauge Micro-fine IV needles (BD Medical, 329461). Lipid droplets released into the media were collected with a pipette in as little media volume as possible and dispensed into 20 μl of HPLC-grade isopropanol in a microcentrifuge tube. To account for any membrane lipids released into the media as a result of the dissection, an equivalent volume of media lacking lipid droplets was collected in isopropanol in a separate microcentrifuge tube. Samples consisting of 40 to 50 individual lipid droplets from ∼30 larvae were stored at −80°C until analysis. The lipid components from the entire volume of each sample were separated and detected by an HPLC-CAD system using a LPG-3400RS quaternary pump, WPS-3000TRS autosampler (maintained at 20°C), TCC-3000RS column oven (maintained at 40°C), Accucore C18 column (150 x 3.0 mm, 2.6 μm particle size), FLD-3100 fluorescence detector (8 ml flow cell maintained at 45°C), and a Dionex Corona Veo charged aerosol detector (Thermo Fisher Scientific). Lipids were separated over an 80 min time range in a multi-step mobile phase gradient as described in (75). Using this protocol, neutral lipids exhibit retention times between 50 and 75 min.

### ER stress reporter assays

*Dgat2*^*sa13945/+*^ fish were crossed to *dgat2*^*sa13945/+*^*;Tg(5xATF6RE:d2GFP)/+* fish (71). At three dpf, embryos were mounted in 3% methylcellulose in embryo media on glass slides and imaged with a Zeiss Axio Zoom V16 microscope equipped with a Zeiss PlanNeoFluar Z one x/0.25 FWD 56 mm objective, AxioCam MRm camera, an EGFP filter, and Zen 2.5 Blue edition software. Bright-field and d2GFP fluorescence images were obtained for wild-type, *dgat2*^*sa13945/+,*^ and *dgat2*^*sa13945*^ embryos, and wild-type dechorionated embryos treated with 2 ug/ml tunicamycin (Sigma, T7765) in embryo media for 24 h. Embryos were collected for subsequent genotyping for both the *sa13945* allele and d2GFP. Mean fluorescence intensity in the yolk sac of each fish was quantitated using Fiji (ImageJ V2.1.0, National Institutes of Health [NIH]; (139)).

### LipoGlo assays

All LipoGlo assays were performed on embryos from crosses of *dgat2*^*sa13945/+*^ x *dgat2*^*sa13945/+;*^*Fus(ApoBb.1-Nluc)/+*, *dgat1a*^*c770/+*^*;dgat1b*^*c773/+*^*;dgat2*^*sa13945*^ x *dgat1a*^*c770/+*^*;dgat1b*^*c773/+*^*;dgat2*^*sa13945/+*^*;Fus(ApoBb.1-Nluc)/+* or *dgat2*^*c765/+*^ x *dgat2*^*c765/+;*^*Fus(ApoBb.1-Nluc)/+*, thus 50% of the fish had a single copy of the LipoGlo (*apoBb.1Nluc/+*) reporter. For full details of LipoGlo protocols see (11). The Nano-Glo reporter system reagents are from Promega Corp., (N1110) (147). For quantitative and size analysis of B-lps, individual embryos were sorted into 96-well plates (USAScientific, #1402–9589) in 100 μl of B-lp stabilization buffer (40 mM EGTA, pH 8.0, 20% sucrose + cOmplete mini, EDTA-free protease inhibitor (Sigma, 11,836,170,001)). Plated embryos were homogenized by sonication in a microplate-horn sonicator (Qsonica Q700 sonicator with a Misonix CL-334 microplate horn assembly). Homogenate was used immediately for quantitation of ApoB-Nanoluc levels and genotyping and subsequently stored at −20°C for later use for analysis of size distribution. ApoB-Nanoluc levels in each embryo were quantified by mixing 40 μl of homogenate with an equal volume of diluted Nanoluc buffer (1:3 NanoGlo buffer:PBS + 0.5% Nanoluc substrate (furimazine)) in a 96-well white OptiPlate (PerkinElmer, 6,005,290), and the plate was read within 2 min of buffer addition on a SpectraMax M5 plate reader (Molecular Devices, top-read chemiluminescent detection with 500 ms integration time). Only fish with quantitative readings above 100,000 RLU were considered to be ApoB-Nanoluc positive and included in the quantitative analysis and further assays. Due to further technical optimization of the quantitative assay and variation in the concentration of the furimazine in different manufacturer lots, the *dgat* triple mutant assays were performed as described above, but in black 96-well OptiPlates (PerkinElmer, 6,005,270), and the assays comparing *dgat2*^*sa13945*^ and *dgat2*^*c765*^ assays were performed in black 96-well OptiPlates but included 4 μl of homogenate with 76 μl of diluted Nanoluc buffer and the plates were read on a BioTek Synergy H1 microplate reader with Gen5 3.05 software (top read chemiluminescent detection with 20 ms integration time). To quantify the size distribution of B-lps, 12 μl of homogenate was combined with 3 μl of 5x loading dye (40% sucrose, 0.25% bromophenol blue in Tris/Borate/EDTA (TBE) buffer) and 12.5 μl of this mixture (10% of larval homogenate) was loaded per well on a 3% native polyacrylamide gel. Each gel included a migration standard of Di-I-labeled human LDL (ThermoFisher Scientific, L3482) and 3 wild-type, 3 *dgat2*^*sa13945/+,*^ and 3 *dgat*^*sa13945*^ embryos from the same clutch (Fig. 4; Fig. S6) or two wild-type siblings, two *dgat2*^*sa13945*^ mutants and two wild-type siblings and two *dgat2*^*c765*^ mutants from the same clutches (Fig. S16). Gels were run at 50 V for 30 min, followed by 125 V for 2 h. One mL of TBE supplemented with 2 μl of Nano-Glo substrate was applied to the surface of the gel, incubated for 5 min, and then imaged with an Odyssey Fc (LI-COR Biosciences) gel imaging system. Images were obtained in the chemiluminescent channel (2 min exposure) for Nanoluc detection and then the 600 nm channel (30 s) for Di-I LDL standard detection. Each lane on the gel was converted to a plot profile in Fiji (ImageJ V2.1.0, National Institutes of Health [NIH], Bethesda, MD; (139)) and divided into LDL, IDL, VLDL and Zero Mobility (ZM) bins based on migration relative to the Di-I LDL standard. Note, we have found that the migration of the Di-I LDL standard shows lot-to-lot variability, thus the bin cutoff values for each lipoprotein class must be re-calibrated for each lot. Cutoff values for the quantitation included in Fig. 4 were 0.3, 1, 1.7 & 2.4 for ZM, VLDL, IDL & LDL, respectively, whereas for the quantitation in Fig. S16, these values were 0.2, 0.98, 1.4 and 2. Pixel intensity from the plot profiles were summed within each bin for each fish to allow for comparison between genotypes. Please refer to (11), Supplemental Software file 1 for the gel analysis template.

### Adipose tissue analysis

Zebrafish heterozygous for the *dgat2*^*sa13945*^ mutation were in-crossed and the resulting progeny were reared as described above. At 33 dpf, zebrafish were treated with 10 mg/ml epinephrine for 15 min to contract melanosomes, then stained with 0.5 ug/ml Nile Red for 45 min as described (148). Zebrafish were then anesthetized with Tricaine and positioned in 3% methylcellulose for imaging under a Leica M205 FA fluorescence stereomicroscope. Fish were thus stained and imaged in groups of 10 in under 5 min per group to avoid batch effects and permit recovery from Tricaine. After acquiring images, fish were revived in fresh water and separated into individual beakers for genotyping. Measurements of body size and total adipose tissue (AT) area were performed in Fiji (ImageJ V2.1.0, National Institutes of Health [NIH], Bethesda, MD; (139)) as previously described (148).

### RNA sequencing sample preparation and analysis

Pairs of *dgat2*^*sa13945/+*^ fish were in-crossed and progeny from three clutches were sorted on 3 dpf for normal *versus* opaque yolks. Embryos were anesthetized with Tricaine and arrayed on the lid of a plastic tissue culture dish in a small volume of embryo media. A scalpel was used to cut off both the head anterior to the yolk sac and the tail and fin, distal to the posterior end of the yolk extension, leaving essentially the yolk sac and dorsal musculature intact. The mid-sections of the embryos were immediately frozen in PCR strip-tubes on dry ice and stored at −80°C until RNA extraction. The head and tail portions were collected in 30 μl of 50 mM NaOH for genotyping as described above. RNA was isolated from a pool of six WT or six *dgat2*^*sa13945*^ mutant sibling mid-bodies from each of the 3 clutches using the Machery-Nagel NucleoSpin RNA XS kit with no carrier RNA (740,902.50, Takara Bio USA Inc.), eluting in 10 μl nuclease-free H_2_O. RNA sample purity was verified with the Agilent RNA 6000 Pico kit and an Agilent 2100 Bioanalyzer (Agilent Technologies). cDNA libraries were constructed from poly(A)-selected RNA using the Illumina TruSeq RNA sample prep kit v2. Six samples were run on an Illumina NextSeq 500 system for a 75 bp indexing run, yielding 55 to 73 million reads per sample.

RNA sequencing data were processed using the nf–core/rnaseq v3.12.0 pipeline (https://doi.org/10.5281/zenodo.1400710). Briefly, adapter sequences were trimmed from the reads, and ribosomal RNA reads were filtered. The remaining reads were mapped to the GRCz11 genome with Ensembl 110 annotation using STAR 2.7.9a and then RNA abundances were quantified using Salmon 1.10.1. RNA abundance data were imported using tximport 1.30.0 and differentially expressed genes were identified using DESeq2 1.42.

## ATGlistatin treatment

*dgat2*^*sa139345*^ homozygous mutants and wild-type fish were treated with vehicle (DMSO), or ATGlistatin (25 μM or 50 μM; Sigma-Aldrich 5,301,510,001) in embryo media for 48 h from 1 to 3 dpf. Bright-field images were obtained following treatment at three dpf and scored for yolk opacity as described above.

## Statistical analyses

All statistical analyses were performed using GraphPad Prism 9 (GraphPad, San Diego, CA). Data are presented as mean ± standard deviation (SD). Statistical tests used are noted in figure legends or text. Unless noted, all experiments were performed with at two to three independent replicates (N). The sample size (number of larvae; n) for each experiment is noted in the figure legends.

## Additional software and databases

Graphing was performed with GraphPad Prism 9 (GraphPad). Sequence alignments were performed with MacVector V15.5 (MacVector, Inc) and zebrafish reference sequences were downloaded from Ensembl (EMBL-EBI) (GRCz11). Micrographs were adjusted and cropped as needed in Fiji (ImageJ V2.1.0, National Institutes of Health [NIH]; (139)) and figures were assembled in Adobe Illustrator 2024 (Adobe Systems). Microsoft Word and Excel were used for manuscript preparation and data analysis, and references were compiled with EndNote X9. The Zebrafish Information Network (ZFIN) was an important source of information for anatomical atlases, nomenclature, and general zebrafish resources (149).

## Data availability

Source data is included in Supporting Information File 4. The RNA sequencing dataset has been deposited in the NCBI Gene Expression Omnibus repository, and is available through GEO Series accession number GSE279170 (https://https://www.ncbi.nlm.nih.gov/geo/query/acc.cgi?acc=GSE279170).

## Supporting information

This article contains supporting information (1, 11, 71, 143, 150, 151, 152, 153, 154, 155, 156, 157, 158, 159, 160, 161).

## Conflict of interest

The authors declare that they have no conflicts of interest with the contents of this article.
