# Supplementary material for: Zebrafish are resilient to the loss of major diacylglycerol acyltransferase enzymes
Source: J Biol Chem. 2024 Nov 6;300(12):107973. doi: 10.1016/j.jbc.2024.107973 (PMC11663968; doi:10.1016/j.jbc.2024.107973)
Supplement: Supplemental figures [file mmc5.pdf]

Zebrafish are resilient to the loss of major diacylglycerol acyltransferase enzymes

Meredith H. Wilson[1,2], Monica R. Hensley[1,2], Meng-Chieh Shen[2], Hsiu-Yi Lu[3], Vanessa H. Quinlivan[2], Elisabeth M. Busch-Nentwich[4], John F. Rawls[3], Steven A. Farber[1,2*]

1 Johns Hopkins University, Dept. of Biology, Baltimore, MD, U.S.A.
2 Carnegie Institution for Science, Dept. of Embryology, Baltimore, MD, U.S.A.
3 Duke University, Dept. of Molecular Genetics and Microbiology, Duke Microbiome Center, Durham, NC, U.S.A.
4 Queen Mary University of London, School of Biological and Behavioural Sciences, London, U.K.

*sfarber3@jh.edu

Supporting Information Supplemental Figures

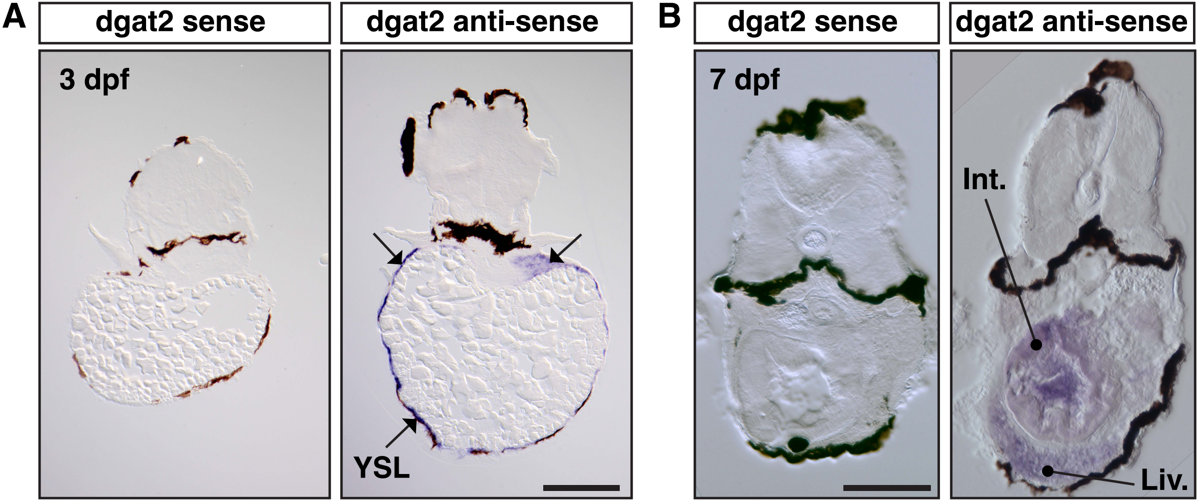
A
dgat2 sense
dgat2 anti-sense
3 dpf
YSL
B
dgat2 sense
dgat2 anti-sense
7 dpf
Int.
Liv.

Figure S1: *dgat2* is expressed in the YSL, liver and intestine of zebrafish embryos and larvae. (A, B) Cross-sections of wild-type AB embryos following *in situ* hybridization for *dgat2* mRNA expression. *Dgat2* expression is visible in the yolk syncytial layer (YSL) at 3 days post fertilization (dpf) (A) and in the liver and intestine at 7 dpf (B). *Dgat2* is expressed in the yolk syncytial layer (YSL), liver and intestine; images are representative of all embryos from 3 experiments at each stage with n = 10 embryos per probe per experiment; Scale bars = 100 μm.

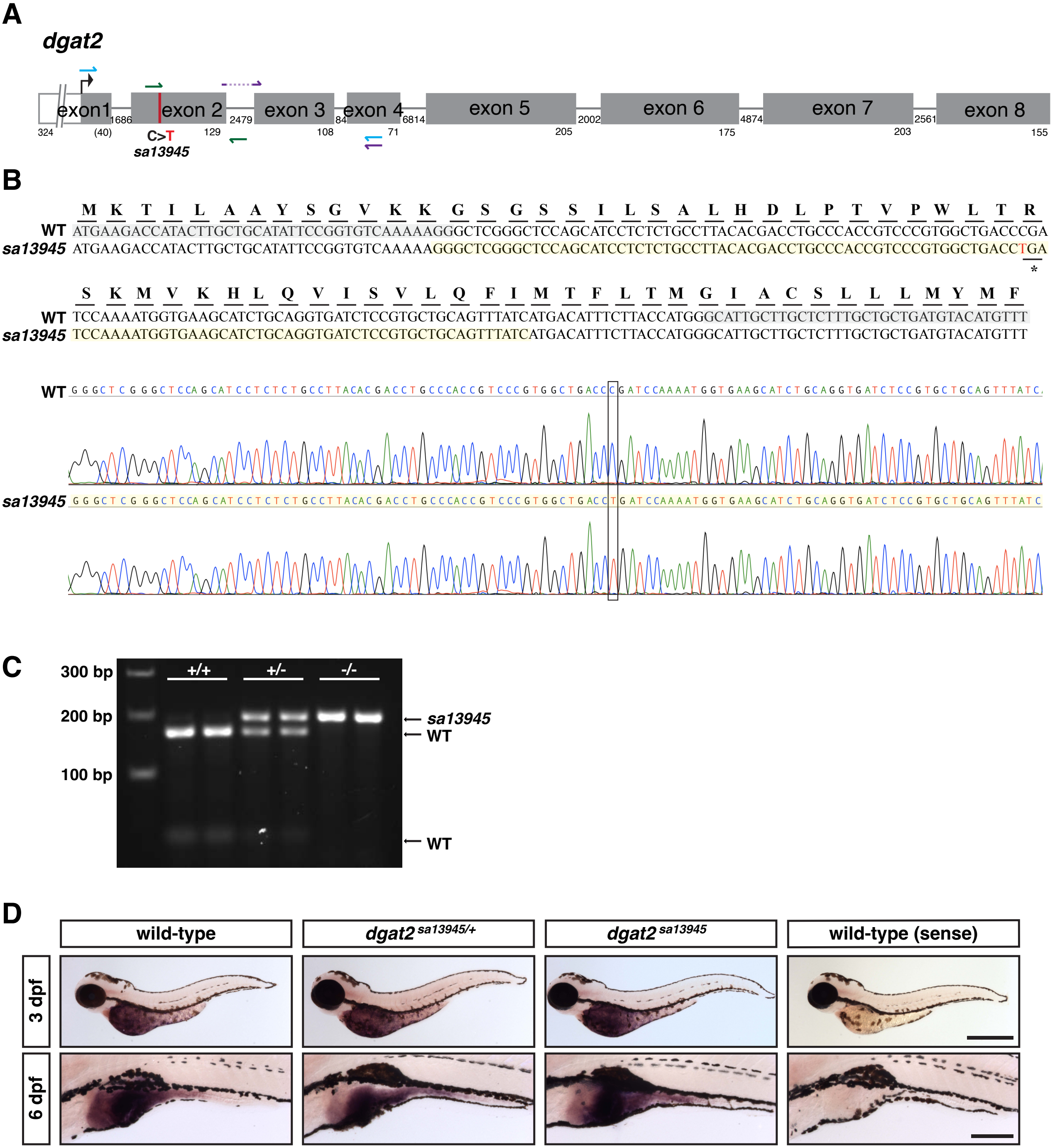
A
dgat2
exon1
exon 2
exon 3
exon 4
exon 5
exon 6
exon 7
exon 8
324 (40) 1686 129 2479 108 84 71 6814 205 2002 175 4874 203 2561 155
C>T
sa13945
B
M K T I L A A Y S G V K K G S G S S I L S A L H D L P T V P W L T R
WT ATGAAGACCATACTTGCTGCATATTCCGGTGTCAAAAAGGGCTCGGGCTCCAGCATCCTCTCTGCCTTACACGACCTGCCCACCGTCCCGTGGCTGACCCGA
sa13945 ATGAAGACCATACTTGCTGCATATTCCGGTGTCAAAAAGGGCTCGGGCTCCAGCATCCTCTCTGCCTTACACGACCTGCCCACCGTCCCGTGGCTGACCTGA
*
S K M V K H L Q V I S V L Q F I M T F L T M G I A C S L L L M Y M F
WT TCCAAAATGGTGAAGCATCTGCAGGTGATCTCCGTGCTGCAGTTTATCATGACATTTCTTACCATGGGCATTGCTTGCTCTTTGCTGCTGATGTACATGTTT
sa13945 TCCAAAATGGTGAAGCATCTGCAGGTGATCTCCGTGCTGCAGTTTATCATGACATTTCTTACCATGGGCATTGCTTGCTCTTTGCTGCTGATGTACATGTTT
WT
sa13945
C
300 bp
200 bp
100 bp
+/+
+/-
-/-
sa13945
WT
WT
D
wild-type
dgat2 sa13945/+
dgat2 sa13945
wild-type (sense)
3 dpf
6 dpf

Figure S2: *dgat2^{sa13945}* mutation details and *in situ* hybridization. (A) Depiction of the *dgat2* gene structure (GRCz11; ENSDARG00000018846, transcript 201 (ENSDART00000066793.7)) highlighting the location of the sa13945 C>T mutation in exon 2 (red line); not to scale, numbers indicate the length of introns and exons (exon 1 from ATG). Green arrows indicate gDNA genotyping primer binding, blue arrows indicate cDNA primer binding and purple arrows indicate qPCR primer binding. (B) cDNA sequence and translation of wild-type *dgat2* and the *dgat2^{sa13945}* nonsense allele from 1 bp to 204 bp, indicating the location of the C>T point mutation at position 100 in exon 2 (c.100C>T; p.Arg34*). Gray shading on wild-type sequence indicates exons 1 & 3. Yellow shading on *sa13945* mutant sequence corresponds to the region shown in the trace files from sanger sequencing of wild-type and *sa13945* mutant cDNA; box highlights the location of the mutated base. (C) Example genomic DNA genotyping gel for the *sa13945* mutation. A 191 bp amplicon is generated using primers SF-MW-122F & SF-MW-36R. The forward primer was designed to introduce a BamH1 restriction site only in the wild-type allele; digestion with BamH1 results in 163 and 28 bp fragments. (D) *In situ* hybridization for *dgat2* expression at 3 and 6 dpf in wild-type, *dgat2$^{sa13945/+}$* and *dgat2^{sa13945}* embryos. *dgat2* continues to be expressed in the yolk syncytial layer (YSL), liver and intestine in *dgat2^{sa13945}* mutants; images are representative of all embryos from 1 experiment at each stage with n = 5 –10 embryos per probe per experiment; Scale bars = 500 μm for 3 dpf, 200 μm for 6 dpf.

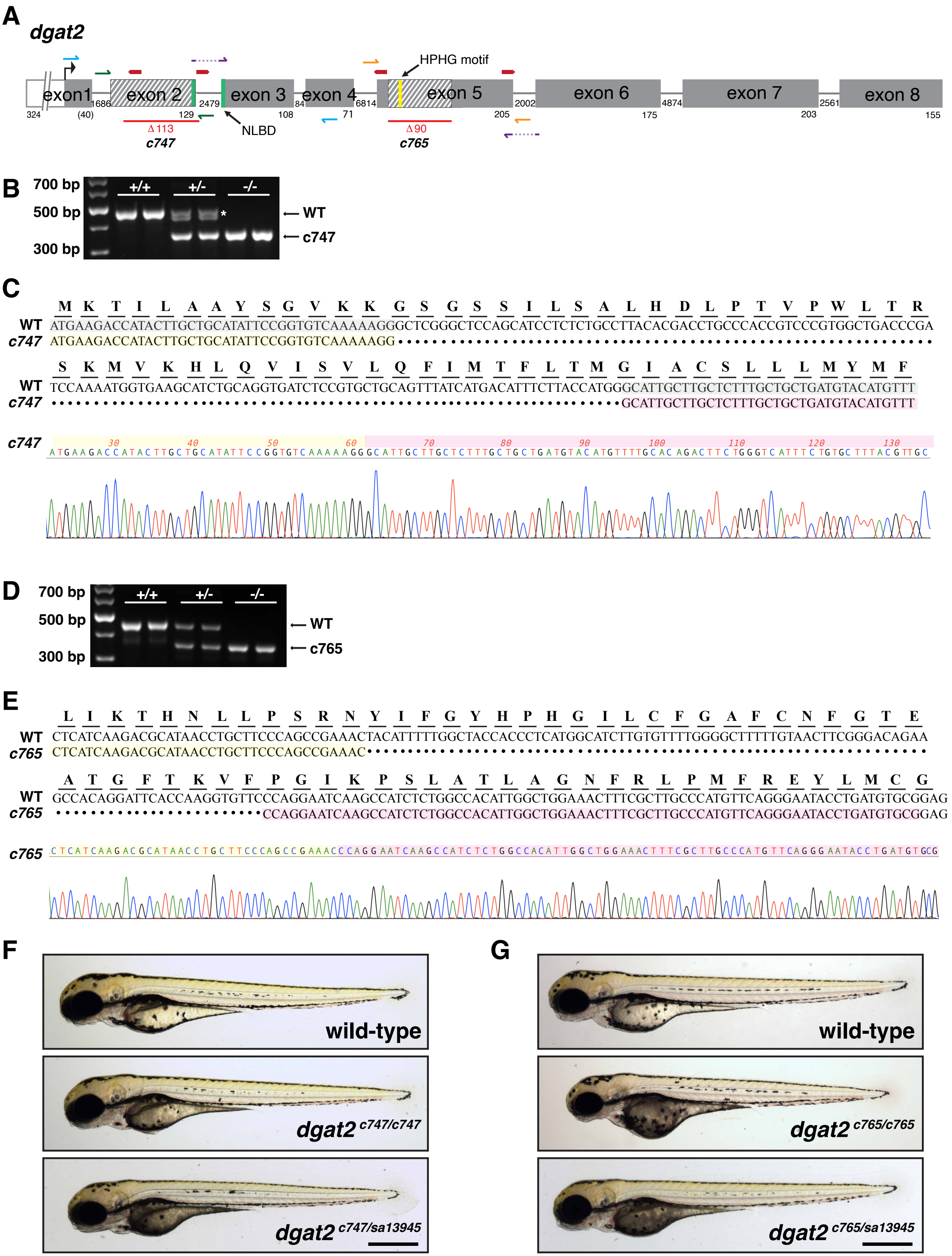
A
dgat2
HPHG motif
exon1
exon 2
exon 3
exon 4
exon 5
exon 6
exon 7
exon 8
1686
2479
84
6814
2002
4874
2561
324
(40)
129
108
71
205
175
203
155
Δ113
c747
NLBD
Δ90
c765
B
700 bp
500 bp
300 bp
+/+
+/-
-/-
WT
c747
C
WT
c747
D
700 bp
500 bp
300 bp
+/+
+/-
-/-
WT
c765
E
WT
c765
F
wild-type
dgat2 c747/c747
dgat2 c747/sa13945
G
wild-type
dgat2 c765/c765
dgat2 c765/sa13945

Figure S3: *dgat2* CRISPR/Cas9 mutant details and complementation crosses. (A) Depiction of the *dgat2* gene structure (GRCz11; ENSDARG00000018846, transcript 201 (ENSDART00000066793.7)) highlighting the location of the CRISPR guide target sequences in exon 2 and exon 5 (red arrows), the putative neutral lipid binding domain (NLBD, green) and the enzymatic HPHG motif (yellow); not to scale, numbers indicate the length of introns and exons (exon 1 from ATG). Red lines below the exons indicate the deleted genomic regions in the *c747* and *c765* alleles; the hashed regions indicate the resulting deletions in the cDNA. Green arrows indicate gDNA genotyping primer binding for the *c747* allele, blue arrows indicate cDNA primer binding for *c747*. Orange arrows indicate gDNA genotyping primers for the *c765* allele, purple arrows indicate cDNA primer binding for *c765*. (B) Example genomic DNA genotyping gel for the *c747* mutation. A 465 bp amplicon is generated for the wild-type allele using primers SF-MW-11F and SF-MW-36R, whereas the *c747* allele is 352 bp due to a deletion of 113 bp in exon 2 into intron 2-3. The doublet between 400 – 500 bp in the heterozygotes may be due to heteroduplex formation. (C) cDNA sequence and translation of wild-type *dgat2* and the *dgat2*^{c747} deletion allele from 1 bp to 204 bp, indicating the resulting in-frame deletion of exon 2 (c.41-169del; p.Gly14_Met56del). Gray shading on the wild-type sequence indicates exon 1 & 3. Yellow and pink shading on the c747 mutant sequence corresponds to the regions shown in the trace file from sanger sequencing of *dgat2*^{c747} cDNA to better highlight the deletion. Part of the putative neutral lipid binding domain (FLXLXXXN; (151,152)(FLVLGVAC in mouse DGAT2 (53), FLTMGIAC in zebrafish Dgat2) is eliminated in the *c747* allele. (D) Example genomic DNA genotyping gel for the *c765* mutation. A 455 bp amplicon is generated for the wild-type allele using primers SF-MW-165F and SF-MW-166R, whereas the *c765* allele is 365 bp due to an in-frame deletion of 90 bp in exon 5, including the HPHG enzymatic motif. (E) cDNA sequence and translation of exon 5 from wild-type *dgat2* and the *dgat2*^{c765} deletion allele (349 bp to 553 bp), indicating the resulting in-frame deletion of 90 bp in the mutant allele (c.385_474del; p.Tyr134_Phe163del). Yellow and pink shading on the *c747* mutant sequence corresponds to the regions shown in the trace file from sanger sequencing of *dgat2*^{c765} cDNA to better highlight the deletion. (F) Representative images of wild-type, *dgat2*^{c747} homozygous mutant and *dgat2*$^{c747/sa13945}$ trans-heterozygous mutant embryos at 3 dpf; Scale = 500 µm. (G) Representative images of wild-type, *dgat2*^{c765} homozygous mutant and *dgat2*$^{c765/sa13945}$ trans-heterozygous mutant embryos at 3 dpf; Scale = 500 µm.

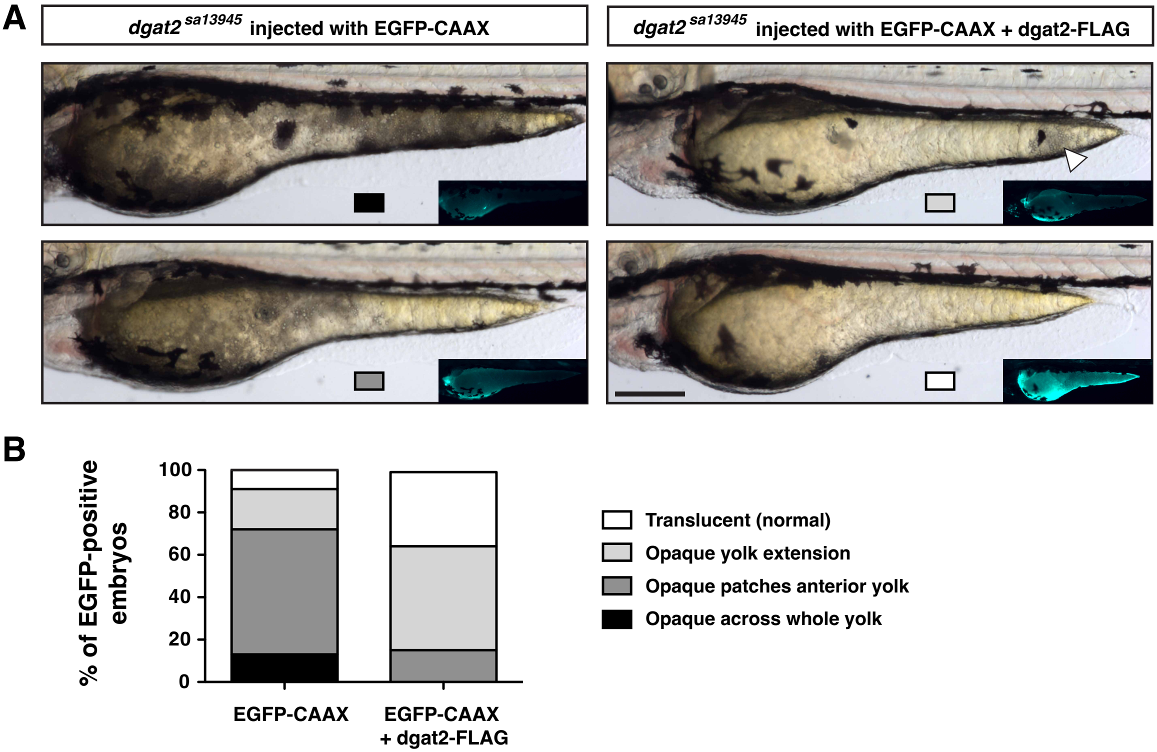
A
dgat2 sa13945 injected with EGFP-CAAX
dgat2 sa13945 injected with EGFP-CAAX + dgat2-FLAG
B
% of EGFP-positive embryos
100
80
60
40
20
0
EGFP-CAAX
EGFP-CAAX + dgat2-FLAG
Translucent (normal)
Opaque yolk extension
Opaque patches anterior yolk
Opaque across whole yolk

Figure S4: Expression of wild-type *dgat2*-FLAG partially rescues the yolk opacity in *dgat2*^{sa13945} embryos. *Dgat2*^{sa13945} embryos were co-injected at the 1-cell stage with CMV: *dgat2-FLAG* and the CMV: *EGFP-CAAX* plasmid, or CMV: *EGFP-CAAX* alone as a control. Bright-field images were obtained of all the embryos that expressed EGFP-CAAX in the YSL at 3 dpf. (A) Representative images of embryos from the two treatment groups depicting different degrees of yolk opacity. Insets highlight corresponding YSL EGFP expression in the embryos. White arrowhead indicates opacity in the yolk extension. Scale = 200 µm. (B) Images were assessed for the degree of yolk opacity into the four denoted bins by a trained lab member who was blinded to the treatment group, and graphed as a percent of total EGFP-positive embryos/treatment group (n = 69 EGFP-CAAX and n = 71 dgat2-FLAG embryos pooled from 3 independent experiments).

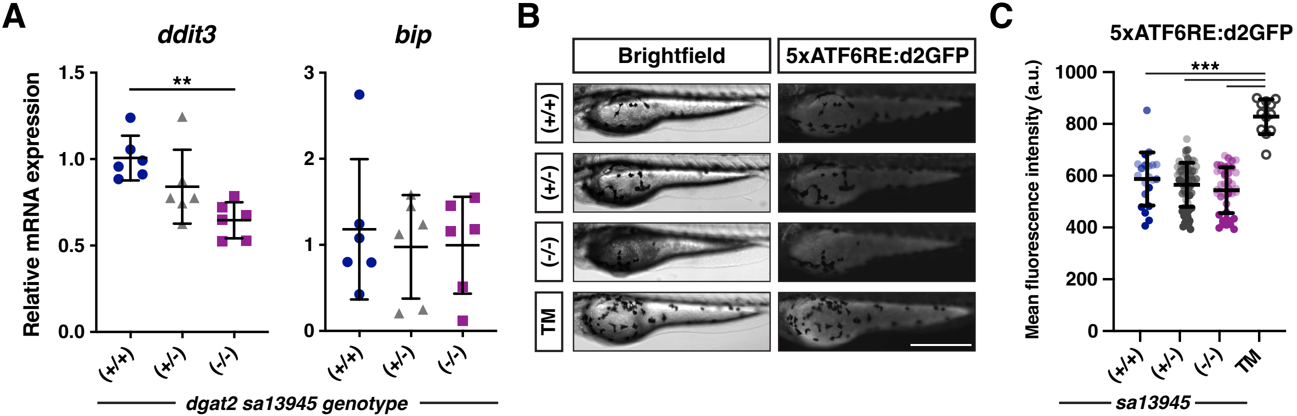

A
ddit3
bip
Relative mRNA expression
1.5
1.0
0.5
0.0
3
2
1
0
**
(+/+)
(+/-)
(-/-)
dgat2 sa13945 genotype
B
Brightfield
5xATF6RE:d2GFP
(+/+)
(+/-)
(-/-)
TM
C
5xATF6RE:d2GFP
Mean fluorescence intensity (a.u.)
1000
800
600
400
200
0
***
(+/+)
(+/-)
(-/-)
TM
sa13945

Figure S5: *Dgat2^{sa13945}* mutants do not show signs of ER stress in the YSL. (A) Quantitative RT-PCR for expression of ER stress-responsive genes *ddit3* and *bip* in wild-type, *dgat2$^{sa13945/+}$*, and *dgat2$^{sa13945/sa13945}$* embryos at 3 dpf (n = 6; 10 pooled fish per sample/genotype, mean ± SD, one-way ANOVA + Tukey's multiple comparison, *ddit3* p = 0.0044, (+/+) vs. (-/-) p = 0.0032, *bip* p = 0.8773, n.s.). (B,C) *Dgat2^{sa13945}* mutants were crossed into the *Tg(5xATF6RE:d2GFP)* ER stress reporter line, which drives expression of a destabilized GFP under the control of ATF6 response elements (71). ATF6 is a transcription factor that resides in the ER and in response to ER stress, it is transported to the Golgi apparatus where it is cleaved, resulting in a 50 kDa cleavage product (ATF6f). The ATF6f fragment translocates to the nucleus and activates transcriptional targets (153). Representative images of d2GFP in the yolk sac of untreated wild-type, *dgat2$^{sa13945/+}$*, and *dgat2^{sa13945}* embryos at 3 dpf and an embryo treated with 2 ug/ml tunicamycin (TM) for 24 h (B). Scale = 500 µm. (C) Mean fluorescence intensity in the yolk sac; results represent data from N = 3 independent experiments (n = 21 (WT), n = 64 (+/-), n = 40 (-/-), n = 12 (TM) fish), mean ± SD. Significance was determined with a non-parametric Kruskall-Wallis ANOVA with multiple comparisons, p <0.0001; TM vs. (+/+) p <0.0004, TM vs. (+/-) and (-/-) p <0.0001).

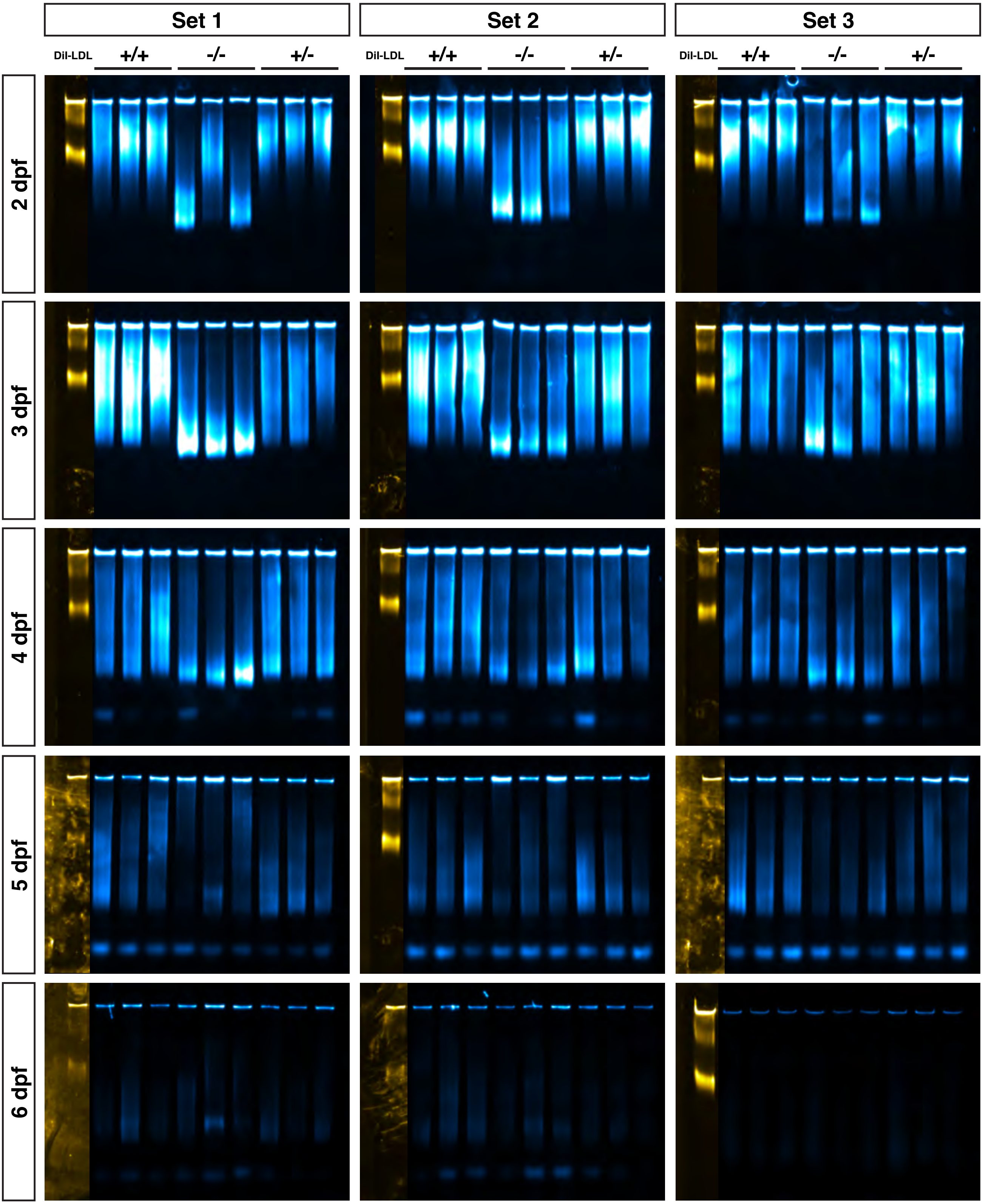
Set 1
Set 2
Set 3
DiI-LDL
+/+
-/-
+/-
2 dpf
3 dpf
4 dpf
5 dpf
6 dpf

Figure S6: LipoGlo PAGE gel primary data. Original gels corresponding to the data in Figure 4D. Each gel shows a composite of the fluorescent DiI-LDL migration standard (yellow) and LipoGlo chemiluminescent emission (blue) from WT (+/+), *dgat2*^{sa13945} (-/-) and *dgat2*$^{sa13945/+}$ (+/-) fish. Note, the gels are overlaid with a piece of thin plastic film prior to imaging, which, if not cleaned properly, can introduce a source of constant background in the 600 nm channel (yellow)(5 dpf set 1 and 3, are especially visible). Gels were analyzed as detailed in (11).

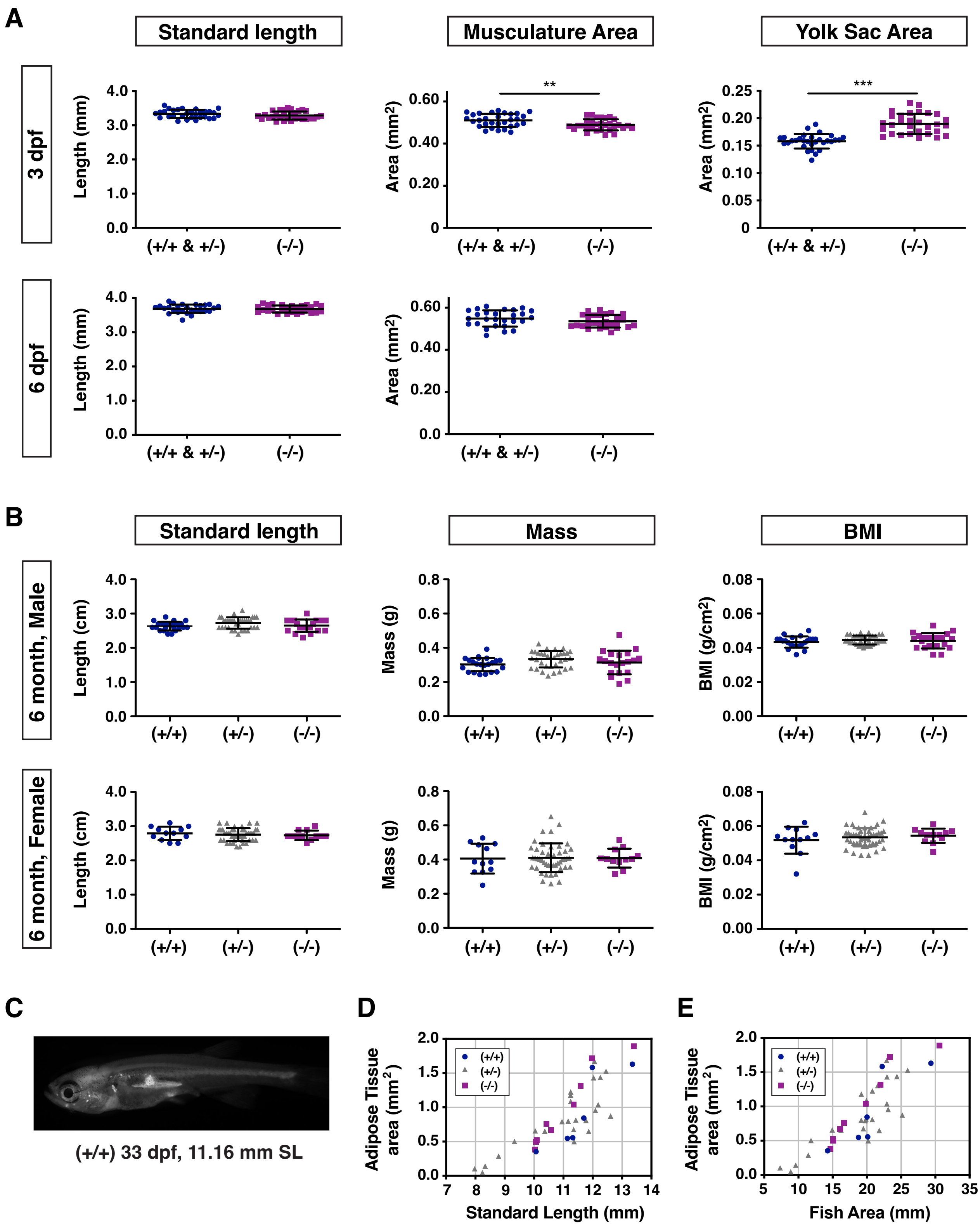

A
Standard length
Musculature Area
Yolk Sac Area
3 dpf
6 dpf
Length (mm)
Area (mm2)
**
***
(+/+ & +/-)
(-/-)
B
Standard length
Mass
BMI
6 month, Male
6 month, Female
Length (cm)
Mass (g)
BMI (g/cm2)
(+/+)
(+/-)
(-/-)
C
(+/+) 33 dpf, 11.16 mm SL
D
E
Adipose Tissue area (mm2)
Standard Length (mm)
Fish Area (mm)

Figure S7: Dgat2 mutant embryos are slightly smaller than siblings but are not different as larvae and adults and does not affect adipose tissue development. (A) Standard length, musculature area and yolk sac area in dgat2^{sa13945} mutants and siblings (WT & *dgat2*$^{sa13945/+}$) were measured as depicted at 3 dpf and 6 dpf (yolk is depleted by 6 dpf); mean ± SD, y axes are shifted from zero for better comparison, n = 27-30 embryos pooled from three independent clutches; two-tailed unpaired t test, * $p < 0.05$, ** $p < 0.01$, and *** $p < 0.001$. (B) Standard length, mass and body mass index (BMI) for males (n = 22 +/+, 34 +/-, 20 -/-) and females (n = 12 +/+, 46 +/-, 12 -/-) from two independent clutches were measured at 6 months of age; mean ± SD, One-way ANOVA, $p > 0.05$ for all metrics. (C-E) Zebrafish siblings that were wild-type (+/+, n = 6), heterozygous (+/-, n = 25) or homozygous (-/-, n = 9) for the *dgat2*^{sa13945} mutation were reared to 33 dpf, stained with Nile Red to permit visualization of adipose tissue, and measured for standard length (SL), total body area, and total adipose tissue area. (C) Representative image of a wild-type *dgat2*^{sa13945} (+/+) zebrafish larvae showing the average standard length for the whole group. (D) Total adipose tissue area plotted against standard length. (E) Total adipose tissue area plotted against total body area.

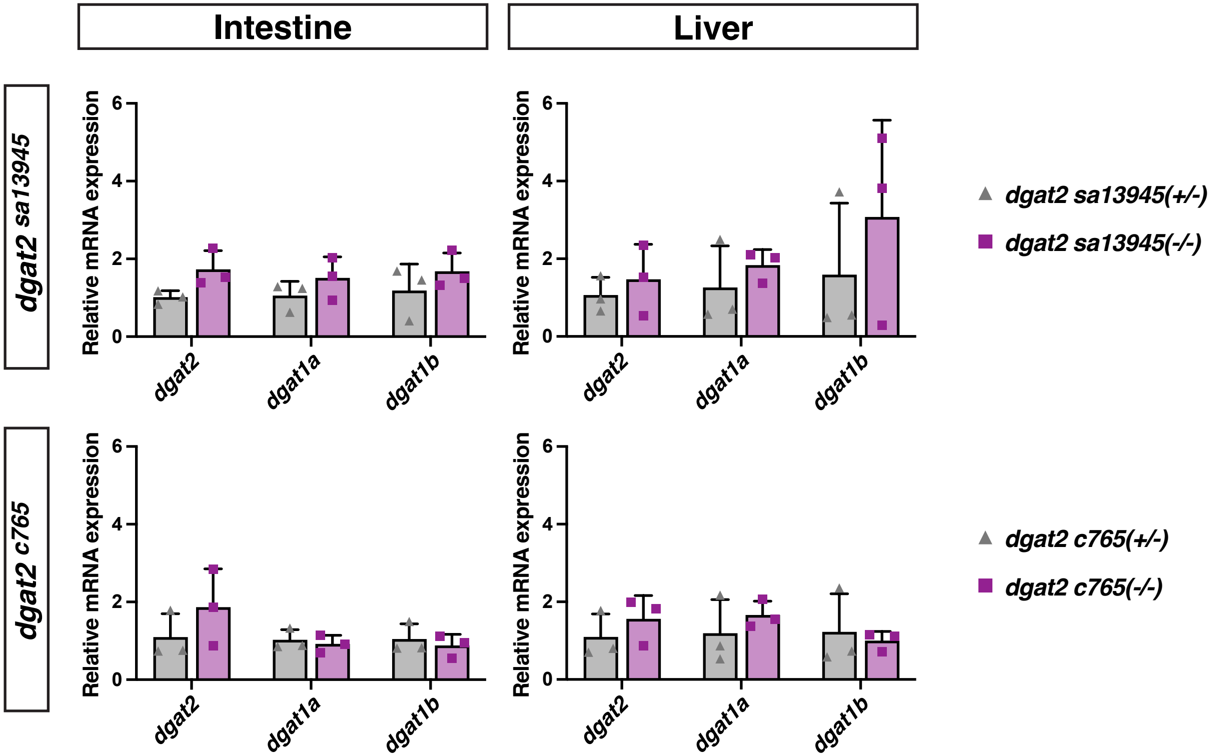

Intestine
Liver
dgat2 sa13945
dgat2 c765
Relative mRNA expression
0
2
4
6
dgat2
dgat1a
dgat1b
dgat2 sa13945(+/-)
dgat2 sa13945(-/-)
dgat2 c765(+/-)
dgat2 c765(-/-)

Figure S8: Expression of *dgat1a* and *dgat1b* are not upregulated in the intestine or liver of adult *dgat2* mutant fish. RNA was isolated from the anterior intestine or liver of 2-year old adult male $dgat2^{sa13945/+}$, $dgat2^{sa13945}$, $dgat2^{c765/+}$, and $dgat2^{c765}$ zebrafish after a 24 hour fast (3 fish per genotype). Equal quantities of RNA per sample was used to synthesize cDNA and quantitative RT-PCR for *dgat2*, *dgat1a* and *dgat1b* was performed in triplicate for each sample along with zebrafish 18S (*rps18*) as a reference gene. Relative gene expression was calculated using the ΔΔCT method (144) between heterozygotes and mutants for each allele; mean ± SD. No significant differences in gene expression were noted (unpaired t-tests with false discovery rate 1.0%, Benjamini, Krieger, and Yekutieli method); See Supporting Information File 4 for p values.

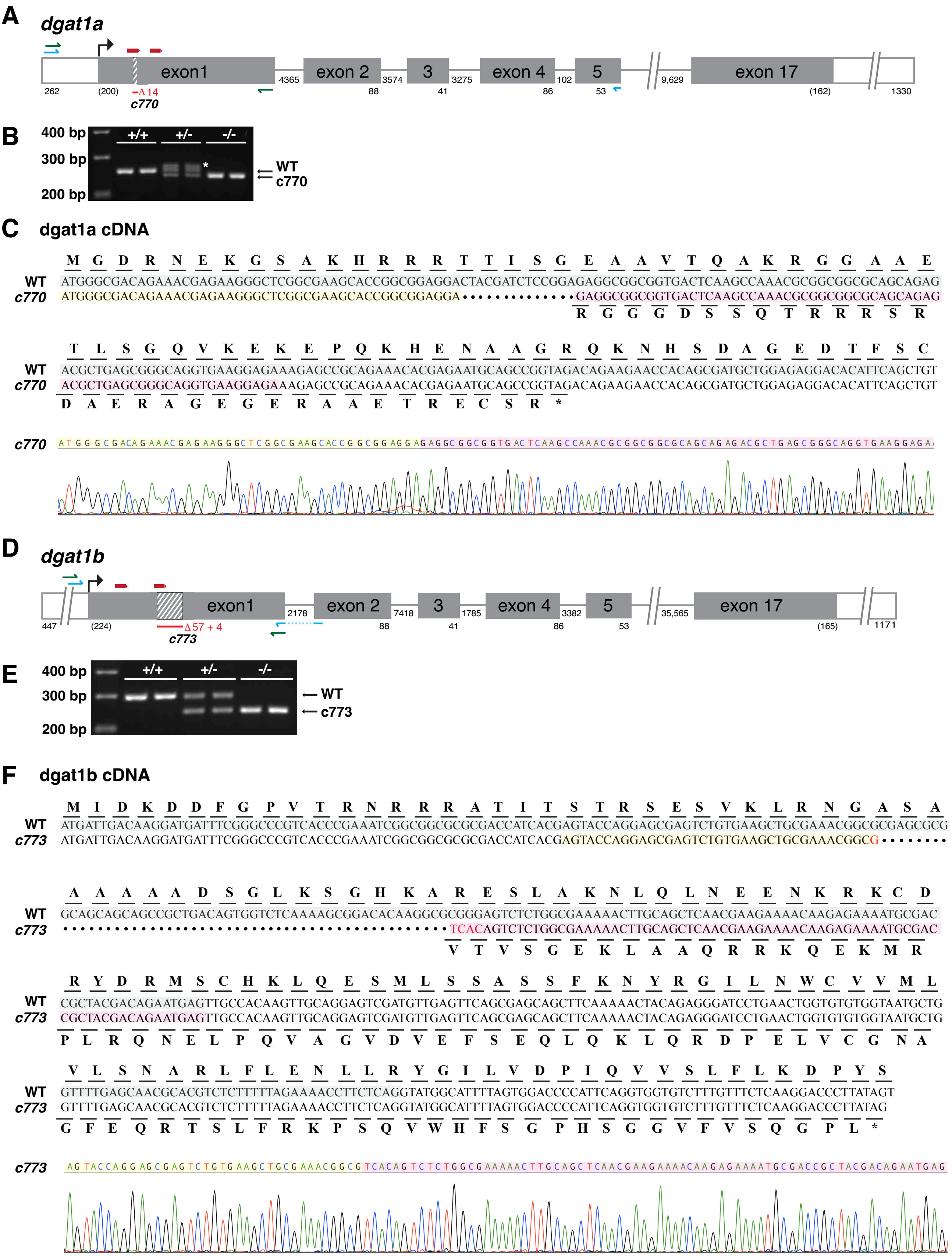

A
dgat1a
exon1
exon 2
3
exon 4
5
exon 17
262
(200)
4365
88
3574
41
3275
86
102
53
9,629
(162)
1330
Δ14
c770
B
400 bp
300 bp
200 bp
+/+
+/-
-/-
*
WT
c770
C
dgat1a cDNA
M G D R N E K G S A K H R R R T T I S G E A A V T Q A K R G G A A E
WT ATGGGCGACAGAAACGAGAAGGGCTCGGCGAAGCACCGGCGGAGGACTACGATCTCCGGAGAGGCGGCGGTGACTCAAGCCAAACGCGGCGGCGCAGCAGAG
c770 ATGGGCGACAGAAACGAGAAGGGCTCGGCGAAGCACCGGCGGAGGA•••••••••••••GAGGCGGCGGTGACTCAAGCCAAACGCGGCGGCGCAGCAGAG
R G G G D S S Q T R R R S R
T L S G Q V K E K E P Q K H E N A A G R Q K N H S D A G E D T F S C
WT ACGCTGAGCGGGCAGGTGAAGGAGAAAGAGCCGCAGAAACACGAGAATGCAGCCGGTAGACAGAAGAACCACAGCGATGCTGGAGAGGACACATTCAGCTGT
c770 ACGCTGAGCGGGCAGGTGAAGGAGAAAGAGCCGCAGAAACACGAGAATGCAGCCGGTAGACAGAAGAACCACAGCGATGCTGGAGAGGACACATTCAGCTGT
D A E R A G E G E R A A E T R E C S R *
c770
D
dgat1b
exon1
exon 2
3
exon 4
5
exon 17
447
(224)
2178
88
7418
41
1785
86
3382
53
35,565
(165)
1171
Δ57 + 4
c773
E
400 bp
300 bp
200 bp
+/+
+/-
-/-
WT
c773
F
dgat1b cDNA
M I D K D D F G P V T R N R R R A T I T S T R S E S V K L R N G A S A
WT ATGATTGACAAGGATGATTTCGGGCCCGTCACCCGAAATCGGCGGCGCGCGACCATCACGAGTACCAGGAGCGAGTCTGTGAAGCTGCGAAACGGCGCGAGCGCG
c773 ATGATTGACAAGGATGATTTCGGGCCCGTCACCCGAAATCGGCGGCGCGCGACCATCACGAGTACCAGGAGCGAGTCTGTGAAGCTGCGAAACGGCG••••••••
A A A A A D S G L K S G H K A R E S L A K N L Q L N E E N K R K C D
WT GCAGCAGCAGCCGCTGACAGTGGTCTCAAAAGCGGACACAAGGCGCGGGAGTCTCTGGCGAAAAACTTGCAGCTCAACGAAGAAAACAAGAGAAAATGCGAC
c773 ••TCACAGTCTCTGGCGAAAAACTTGCAGCTCAACGAAGAAAACAAGAGAAAATGCGAC
V T V S G E K L A A Q R R K Q E K M R
R Y D R M S C H K L Q E S M L S S A S S F K N Y R G I L N W C V V M L
WT CGCTACGACAGAATGAGTTGCCACAAGTTGCAGGAGTCGATGTTGAGTTCAGCGAGCAGCTTCAAAAACTACAGAGGGATCCTGAACTGGTGTGTGGTAATGCTG
c773 CGCTACGACAGAATGAGTTGCCACAAGTTGCAGGAGTCGATGTTGAGTTCAGCGAGCAGCTTCAAAAACTACAGAGGGATCCTGAACTGGTGTGTGGTAATGCTG
P L R Q N E L P Q V A G V D V E F S E Q L Q K L Q R D P E L V C G N A
V L S N A R L F L E N L L R Y G I L V D P I Q V V S L F L K D P Y S
WT GTTTTGAGCAACGCACGTCTCTTTTTAGAAAACCTTCTCAGGTATGGCATTTTAGTGGACCCCATTCAGGTGGTGTCTTTGTTTCTCAAGGACCCTTATAGT
c773 GTTTTGAGCAACGCACGTCTCTTTTTAGAAAACCTTCTCAGGTATGGCATTTTAGTGGACCCCATTCAGGTGGTGTCTTTGTTTCTCAAGGACCCTTATAG
G F E Q R T S L F R K P S Q V W H F S G P H S G G V F V S Q G P L *
c773

Figure S9: *dgat1* CRISPR/Cas9 mutant details. (A) Depiction of the *dgat1a* gene structure (GRCz11; ENSDARG00000103503, transcript 201 (ENSDART00000158946.2)) highlighting the location of the two CRISPR target sequences in exon 1 (red arrows) and the resulting 14 bp deletion in exon 1 (red line below exon and hashing). Green arrows indicate gDNA genotyping primer binding, blue arrows indicate cDNA primer binding. (B) Example genomic DNA genotyping gel for the *dgat1a*^{c770} mutation. A 240 bp amplicon is generated for the wild-type allele using primers SF-MRH-169F and SF-MRH-170R, whereas the *c770* allele is 226 bp due to a deletion of 14 bp in exon 1. The asterisk indicates an extra band in the heterozygotes that is likely due to heteroduplex formation. (C) cDNA sequence and translation of wild-type *dgat1a* and the *dgat1a*^{c770} deletion allele from 1 bp to 204 bp, indicating the 14 bp deletion leading to a premature termination codon in exon 1 (c.47_60del; p.Thr15ArgfsTer34). Gray shading on the wild-type sequence indicates exon 1. Yellow and pink shading on the c770 mutant sequence corresponds to the regions shown in the trace file from sanger sequencing of *dgat1a*^{c770} cDNA to better highlight the deletion. (D) Depiction of the *dgat1b* gene structure (GRCz11; ENSDARG00000054914, transcript 201 (ENSDART00000077185.5)) highlighting the location of the two CRISPR target sequences in exon 1 (red arrows) and the location of the resulting 57 bp deletion + 4 bp insertion in exon 1 (red line below exon and hashing). Green arrows indicate gDNA genotyping primer binding, blue arrows indicate cDNA primer binding. (E) Example genomic DNA genotyping gel for the *dgat1b*^{c773} mutation. A 297 bp amplicon is generated for the wild-type allele using primers SF-MRH-177F and SF-MRH-178R, whereas the *c773* allele is 244 bp due to a deletion of 57 and a 4 bp insertion in exon 1. (F) cDNA sequence and translation of wild-type *dgat1b* and the *dgat1b*^{c773} deletion allele from 1 bp to 414 bp, indicating the 57 bp deletion and 4 bp insertion (red text) (c.98_154delinsTCAC; p.Ala33ValfsTer88). Gray shading on the wild-type sequence indicates exon 1 & 3. Yellow and pink shading on the c773 mutant sequence corresponds to the regions shown in the trace file from sanger sequencing of *dgat1b*^{c773} cDNA to better highlight the deletion.

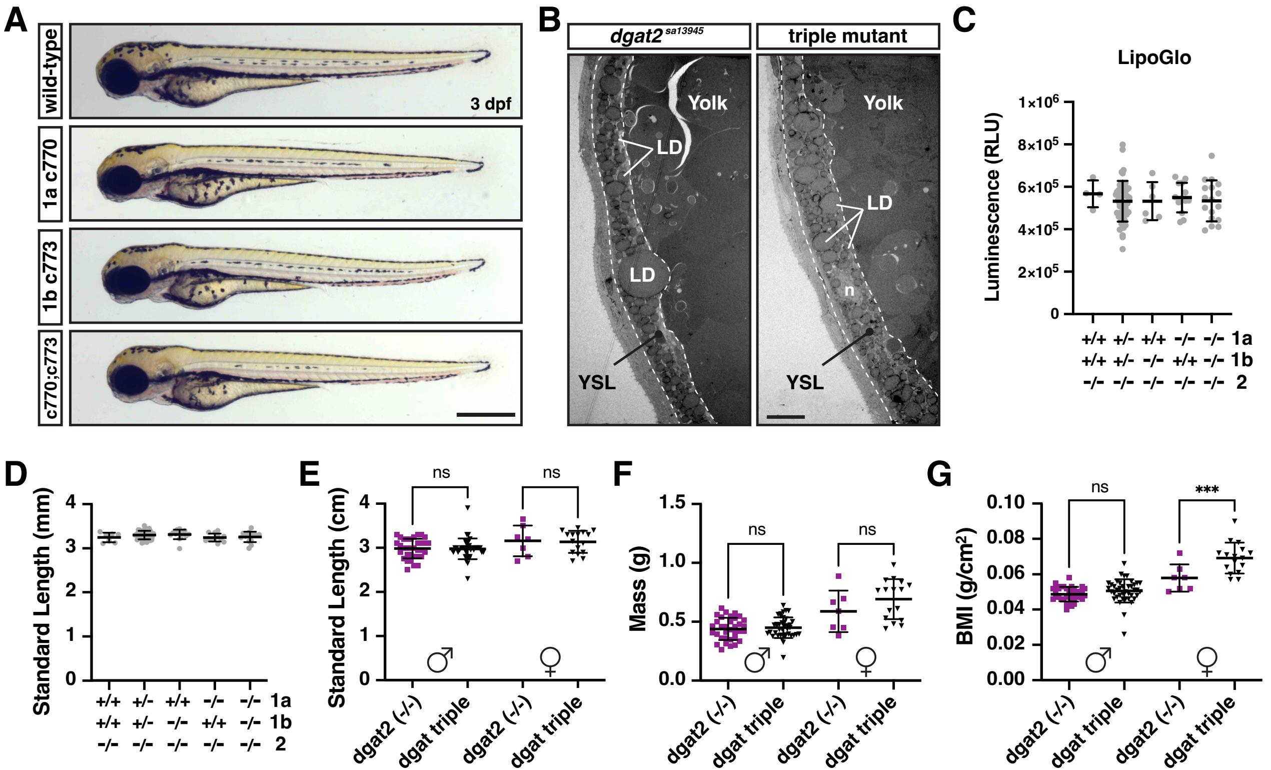
A
wild-type
3 dpf
1a c770
1b c773
c770;c773
B
dgat2 sa13945
triple mutant
Yolk
LD
LD
YSL
Yolk
LD
n
YSL
C
LipoGlo
Luminescence (RLU)
1×10^6
8×10^5
6×10^5
4×10^5
2×10^5
0
+/+ +/- +/+ -/- -/- 1a
+/+ +/- -/- +/+ -/- 1b
-/- -/- -/- -/- -/- 2
D
Standard Length (mm)
4
3
2
1
0
+/+ +/- +/+ -/- -/- 1a
+/+ +/- -/- +/+ -/- 1b
-/- -/- -/- -/- -/- 2
E
Standard Length (cm)
ns
ns
dgat2 (-/-)
dgat triple
dgat2 (-/-)
dgat triple
F
Mass (g)
1.5
1.0
0.5
0.0
ns
ns
dgat2 (-/-)
dgat triple
dgat2 (-/-)
dgat triple
G
BMI (g/cm2)
0.10
0.08
0.06
0.04
0.02
0.00
ns
***
dgat2 (-/-)
dgat triple
dgat2 (-/-)
dgat triple

Figure S10: dgat triple mutant additional data. (A) Representative images of wild-type, *dgat1a*^{c770}, *dgat1b*^{c773} and *dgat1a*^{c770};*dgat1b*^{c773} homozygous mutant embryos from an in-cross of *dgat1a*$^{c770/+}$;*dgat1b*$^{c773/+}$;*dgat2*$^{sa13945/+}$ parents at 3 dpf; Scale = 500 µm. (B) Representative transmission electron micrographs of the yolk and YSL from *dgat2*^{sa13945} and *dgat1a*^{c770};*dgat1b*^{c773};*dgat2*^{sa13945} triple mutants; dashed lines delineate the YSL region, n = nucleus, LD = lipid droplet, scale = 10 µm. (C) LipoGlo luminescence (RLU = relative luminescence units) in noted *dgat* genotypes at 3 dpf. Results represent pooled data from 6 clutches, n = 4 – 51 embryos/genotype; mean ± SD; One-way ANOVA, p = 0.9289. Note: the luminescence values in these assays are much lower than those in Figure 4B because this set of assays was performed in black 96-well plates, as opposed to the initial assays which were performed in white 96-well plates, which amplify the luminescence values. (D) Standard length was measured siblings from a cross of *dgat1a*$^{c770/+}$;*dgat1b*$^{c773/+}$;*dgat2*^{sa13945} parents at 3 dpf; mean ± SD, n = 7– 41 embryos per genotype pooled from two independent clutches; One-way ANOVA, p = 0.2051. (E-G) Standard length, mass and body mass index (BMI) data for *dgat2*^{sa13945} and *dgat1a*^{c770};*dgat1b*^{c773};*dgat2*^{sa13945} triple mutants at 6 months of age that were raised in separate, but adjacent tanks. Data is separated by sex: males (n = 34 *dgat2*^{sa13945}, 40 *dgat* triple mutants) and females (n = 7 *dgat2*^{sa13945}, 16 *dgat* triple mutants); mean ± SD, One-way ANOVA, p = 0.0548 for (E), p <0.0001 for (F)(statistical differences were due to sex and not genotype), p <0.0001 for (G), *** denotes p = 0.001.

Gene_ID	chr	log2FC	padj	Gene_Name
ENSDARG00000070885	22	8.13	9.61E-07	NA (anti-sense)
ENSDARG00000071012	17	4.36	1.41E-07	ifit14
ENSDARG00000075996	9	4.33	1.93E-02	znfl1b
ENSDARG00000007086	16	3.83	7.46E-15	aqp10a
ENSDARG00000008948	10	3.57	6.92E-03	pla2g3
ENSDARG00000095820	22	3.47	1.89E-02	NA (lincRNA)
ENSDARG00000079227	12	3.36	3.99E-17	plekhs1.1
ENSDARG00000074210	11	3.35	1.04E-03	NA, possibly ADP-ribosylation factor 4-like
ENSDARG00000104919	14	3.12	2.26E-10	NA, possibly related to YrdC domain-containing protein
ENSDARG00000096110	25	3.04	2.26E-10	zgc:123278
ENSDARG00000094929	16	2.97	6.20E-03	apoa4b.3
ENSDARG00000035852	7	2.83	1.65E-05	cart3
ENSDARG00000104007	10	2.79	2.54E-05	pcdh1g1
ENSDARG00000087311	15	2.65	2.77E-07	lgals9l5
ENSDARG00000094741	13	2.52	2.84E-04	HTRA2
ENSDARG00000095409	12	2.47	1.70E-03	NA, possibly GP2-like
ENSDARG00000062788	14	2.47	8.67E-07	irg1l
ENSDARG00000113315	12	2.26	2.94E-04	NA, possibly apha-tectorin-like protein
ENSDARG00000069139	10	2.23	1.96E-12	GRIK1
ENSDARG00000039352	13	2.11	2.26E-10	pald1b
ENSDARG00000092719	12	2.05	4.05E-06	NA (pseudogene)
ENSDARG00000117266	12	2.03	1.58E-03	NA (lincRNA)
ENSDARG00000069630	18	2.02	1.55E-05	tat
ENSDARG00000053480	7	2.02	2.39E-07	aqp9b
ENSDARG00000038296	3	1.98	4.21E-22	tmem86b
ENSDARG00000096739	12	1.95	3.95E-03	NA, possibly E3 ubiquitin ligase TRIM39-like isoform X1
ENSDARG00000076196	12	1.93	4.29E-02	NA, possibly GP2-like
ENSDARG00000101040	2	1.92	2.74E-02	ccl20a.3
ENSDARG00000096933	16	1.86	1.12E-02	si:ch211-135n15.2
ENSDARG00000101665	10	1.74	1.45E-07	pcdh1g11
ENSDARG00000055365	20	1.73	8.35E-03	si:dkey-25e12.3, possibly adenosine 5'monophosphoramidase HINT3
ENSDARG00000074150	12	1.51	2.60E-02	NA, possibly GP2-like
ENSDARG00000022730	20	1.50	3.61E-03	AASDH
ENSDARG00000100538	24	-9.06	2.89E-09	NA, possibly a transposon related protein
ENSDARG00000089794	24	-8.43	1.80E-07	NA, possibly sterile apha motif domain-containing protein 3 (SAMD3)
ENSDARG00000101486	SC	-7.77	1.55E-05	zgc: 77614, clone-based ensembl gene (SC: scaffold)
ENSDARG00000093389	5	-6.51	9.75E-03	NA, possibly coiled-coil alpha helical rod protein 1 isoform X2
ENSDARG00000096299	19	-6.13	4.77E-02	NA, possibly NKD inhibitor of WNT signaling pathway 3
ENSDARG00000097985	17	-4.27	1.39E-03	lincRNA
ENSDARG00000116065	13	-3.15	3.61E-03	NA, possibly RNA-directed DNA polymerase from mobile element jockey
ENSDARG00000038378	15	-2.30	1.55E-02	sagb
ENSDARG00000103347	23	-2.19	2.27E-08	cyp2aa3
ENSDARG00000033610	10	-2.05	3.90E-02	morn5
ENSDARG00000001437	23	-2.02	5.41E-03	slc2a1a
ENSDARG00000104057	10	-1.96	2.59E-03	pcdh1g14
ENSDARG00000104387	10	-1.94	9.61E-07	SLC4A5
ENSDARG00000079946	16	-1.89	2.11E-04	sqlea
ENSDARG00000062632	25	-1.79	4.98E-02	NA, possibly dual oxidase
ENSDARG00000117636	1	-1.76	1.29E-02	lincRNA
ENSDARG00000075600	10	-1.72	2.74E-03	NA, possibly CSPG4-like isoform X2
ENSDARG00000045089	13	-1.68	5.05E-07	apcs
ENSDARG00000100292	10	-1.66	8.94E-03	fam166b
ENSDARG00000099447	10	-1.63	5.51E-04	pcdh1g18
ENSDARG00000089066	7	-1.52	8.67E-07	NHSL2

Figure S11: Differentially expressed genes in *dgat2*^{sa13945} mutants.
RNA was isolated from pools of 6 *dgat2*^{sa13945} mutant or 6 wild-type sibling mid-bodies from each of three separate clutches (resulting in 3 samples per genotype). RNA sequencing identified a total of 102 upregulated and 65 downregulated differentially expressed genes in *dgat2*^{sa13945} mutants meeting an adjusted p value (padj) <0.05 (See Supporting Information File 2 for full list). Twenty-four of these genes were located on chromosome 10, many of which are within 12 Mb of the *dgat2* gene locus. Shown here are the differentially expressed genes meeting a log2fold change (log2FC) cutoff of ≥1.5 or ≤-1.5. Green shading indicates upregulated genes, red shading denotes downregulated genes, blue shading highlights genes on chromosome 10 (chr). Many of the genes are not annotated in Ensembl (NA), but BLASTP searches sometimes provided possible gene information.

Additional discussion of the RNA sequencing analysis related to Figure S11:

While the RNA sequencing analysis comparing transcripts in wild-type vs. *dgat2*^{sa13945} mutants did not reveal any candidates for the residual triacylglycerol synthesis activity, there was differential expression of some interesting genes. One gene of interest upregulated in *dgat2* mutants was *tmem86b*, a lysoplasmalogenase found in liver and intestinal microsomes that has been suggested to regulate the levels of plasmalogens in the cell (154,155). Plasmalogens are glycerolipids that contain an ether bond at the sn-1 position, typically these are found as ether phospholipids, but “neutral plasmalogens” or ether analogues of triacylglycerols (monoalkyl-diacylgycerols, MADAG) also exist (156). These neutral ether lipids can be synthesized by DGAT1, DGAT2, MGAT2 and MGAT3 (157). Previous lipidomics analyses of the zebrafish yolk indicate the presence of low concentrations of plasmalogens, although these were primarily ether phospholipids (1). It would be curious to perform similar lipidomics analyses on the *dgat2* mutant yolk and isolated lipid droplets, as the increased *tmem86b* expression suggests possible changes in ether lipid levels, which could affect membrane dynamics and in turn influence phenotypic changes we see in the mutants. *dgat2* mutants also had upregulation of aquaporin 10a (*aqp10a*). This gene encodes an aquaglyceroporin, which transports both water and glycerol across the plasma membrane of adipocytes in humans (158,159) and is also found in the capillary endothelium and gastroenteropancreatic cells of the intestinal duodenum and jejunum (160). Though *in situ* hybridization data suggests it is not expressed in the YSL of wild-type zebrafish embryos (161), it is expressed in the intestine, liver and kidney of adults and has been demonstrated to transport water, glycerol and urea (162). In adipocytes, it is pH-sensitive and aids in the release of glycerol into the circulation following lipolysis (158). Perhaps it is upregulated in the YSL of *dgat2* mutants as an alternate mechanism to secrete yolk platelet and/or lipid-droplet-derived glycerol out of the yolk sac, thus helping to alleviate the back-up in B-lp-based lipid secretion. Alternatively, its upregulation might help to explain the severe edema often noted in quadruple *mogat3b;dgat1a;dgat1b;dgat2* mutants.

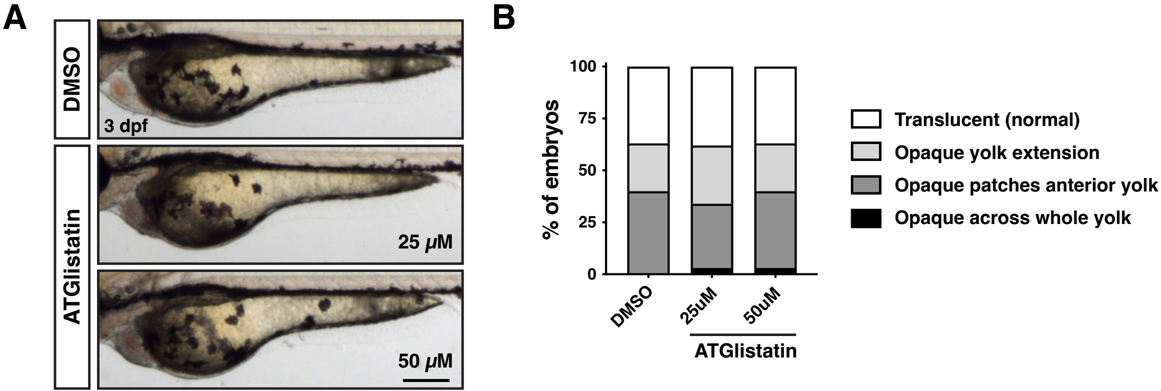
A
DMSO
3 dpf
ATGlistatin
25 μM
50 μM
B
% of embryos
100
75
50
25
0
DMSO
25uM
50uM
ATGlistatin
Translucent (normal)
Opaque yolk extension
Opaque patches anterior yolk
Opaque across whole yolk

Figure S12: Inhibition of ATGL does not rescue yolk opacity in *dgat2*^{sa13945} mutants. *dgat2*^{sa139345} homozygous mutants were treated with vehicle (DMSO) or ATGlistatin (25 μM or 50 μM) for 48 hours from 1 to 3 dpf. Bright-field images were obtained following treatment at 3 dpf. (A) Representative images of embryos from the three treatment groups; scale = 200 μm. (B) Images of 10 embryos from each of 3 clutches of *dgat2*^{sa13945} mutants were scored for the degree of yolk opacity, binned into the four noted categories and expressed as a percent of total embryos per treatment.

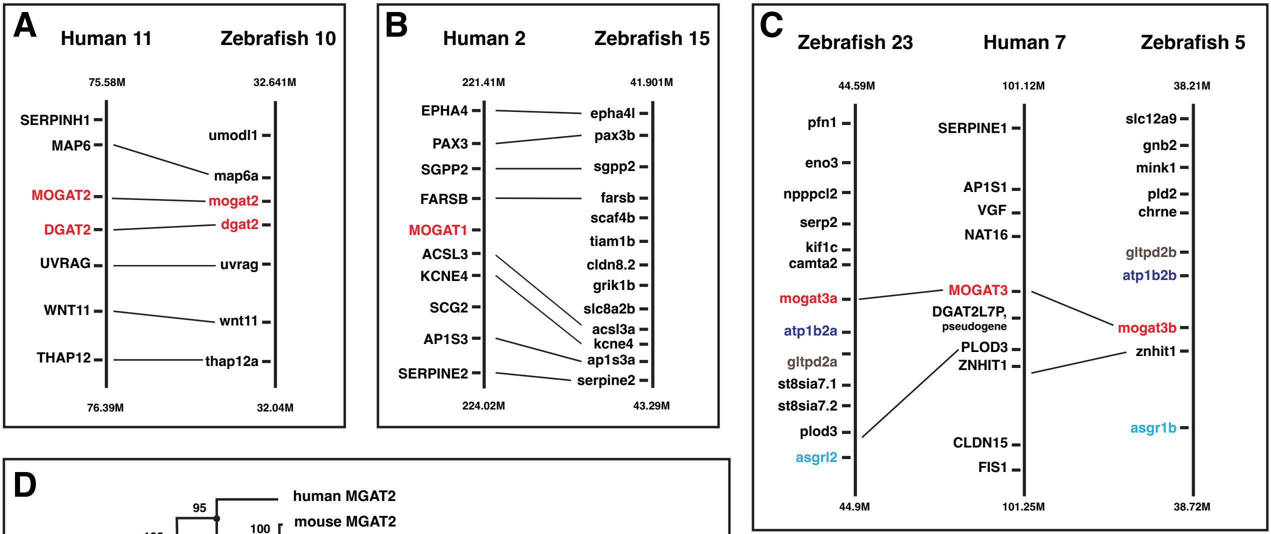

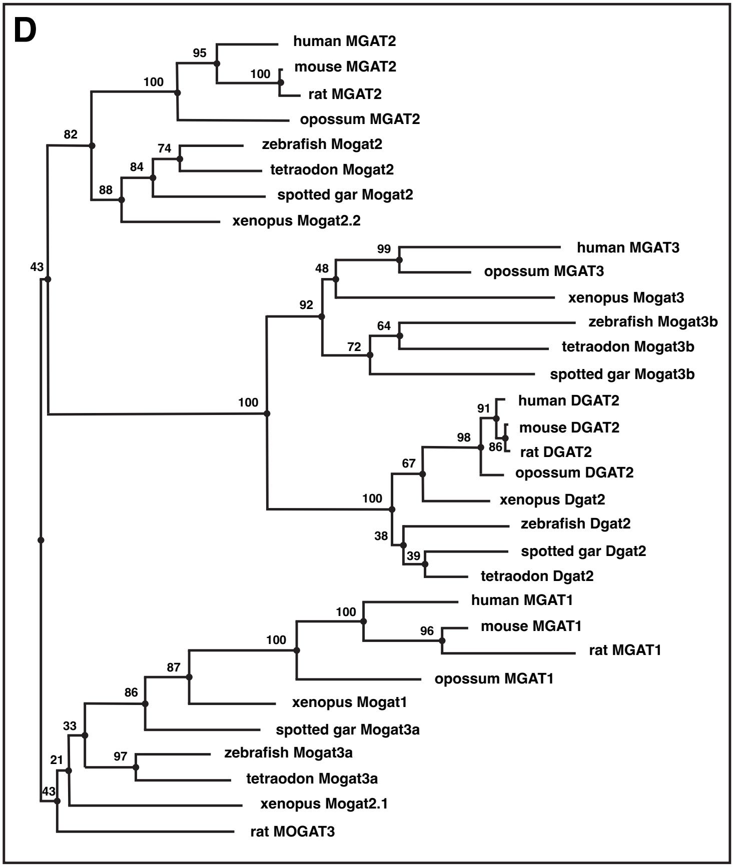

E

Human

	DGAT2	MGAT1	MGAT2	MGAT3
DGAT2	100.0	42.5	39.4	43.3
MGAT1	58.5	100.0	53.1	42.7
MGAT2	56.7	71.3	100.0	45.9
MGAT3	61.6	62.3	64.3	100.0

F

Zebrafish

	Dgat2	Mogat2	Mogat3a	Mogat3b
Dgat2	100.0	45.7	42.1	48.8
Mogat2	60.7	100.0	61.5	44.9
Mogat3a	59.6	78.8	100.0	43.4
Mogat3b	66.5	60.7	59.8	100.0

Identity score (%)

Similarity score (%)

Figure S13: **Zebrafish Mogat3b is more related to Dgat2 than Mogat2 or Mogat3a.** Syntenic analysis indicates that zebrafish *mogat2* and *dgat2* are orthologs of human *MGAT2* and *DGAT2* (A), zebrafish do not have an ortholog of human MGAT1 (B) and zebrafish *mogat3a* and *mogat3b* both share synteny with human *MOGAT3* (C). (D) A phylogenetic tree was generated using DGAT2/dgat2 and MGAT/Mogat amino acid sequences from human (*Homo sapien*), mouse (*Mus musculus*), rat (*Rattus norvegicus*), opossum (*Mondelphis domestica*), tropical clawed frog (*Xenopus tropicalis*), spotted gar (*Lepisosteus oculatus*), tetraodon (*Tetraodon nigroviridis*) and zebrafish (*Danio rerio*). Bootstrap support for each clade is reported (>70 interpreted as significant). Zebrafish *mogat3a* and *mogat3b* did not result from the teleost-specific whole genome duplication event, but rather an earlier duplication event as evidenced by the presence of *mogat3a* and *mogat3b* in another ray-finned fish, spotted gar (a member of the holostei infraclass). Zebrafish Mogat3b groups more closely with Dgat2 than to the other mogat genes. (E) Identity and similarity scores between the human DGAT2 and MGAT protein sequences. (F) Identity and similarity scores between zebrafish Dgat2 and Mogat protein sequences.

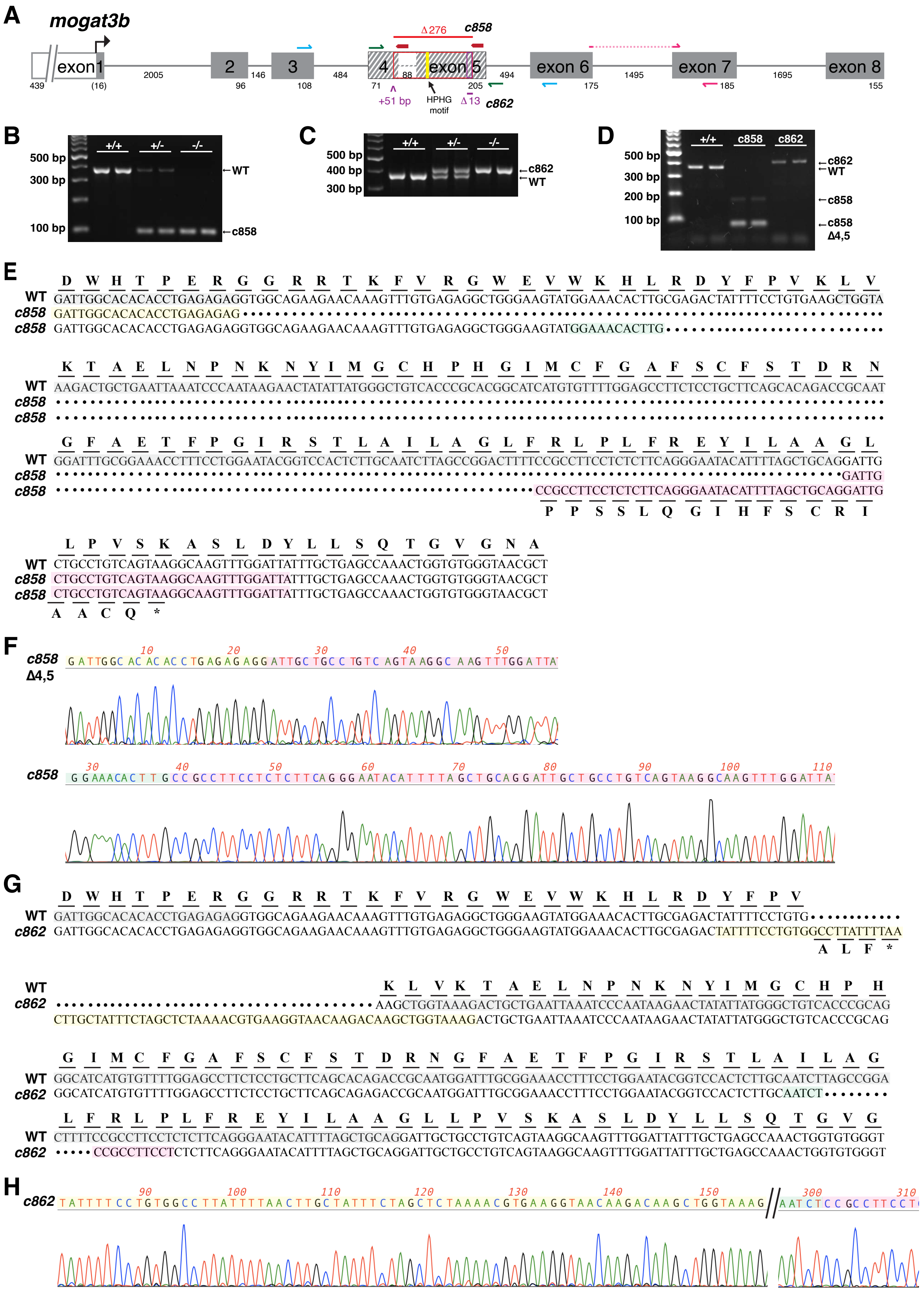
A
mogat3b
exon1
2
3
4
exon 5
exon 6
exon 7
exon 8
Δ276
c858
439
(16)
2005
96
146
108
484
71
88
205
494
175
1495
185
1695
155
+51 bp
HPHG motif
Δ13
c862
B
+/+
+/-
-/-
500 bp
300 bp
100 bp
←WT
←c858
C
+/+
+/-
-/-
500 bp
400 bp
300 bp
←c862
←WT
D
+/+
c858
c862
500 bp
300 bp
200 bp
100 bp
←c862
←WT
←c858
←c858 Δ4,5
E
WT GATTGGCACACACCTGAGAGAGGTGGCAGAAGAACAAAGTTTGTGAGAGGCTGGGAAGTATGGAAACACTTGCGAGACTATTTTCCTGTGAAGCTGGTA
c858 GATTGGCACACACCTGAGAGAG
c858 GATTGGCACACACCTGAGAGAGGTGGCAGAAGAACAAAGTTTGTGAGAGGCTGGGAAGTATGGAAACACTTG
WT AAGACTGCTGAATTAAATCCCAATAAGAACTATATTATGGGCTGTCACCCGCACGGCATCATGTGTTTTGGAGCCTTCTCCTGCTTCAGCACAGACCGCAAT
WT GGATTTGCGGAAACCTTTCCTGGAATACGGTCCACTCTTGCAATCTTAGCCGGACTTTTCCGCCTTCCTCTCTTCAGGGAATACATTTTAGCTGCAGGATTG
c858 GATTG
c858 CCGCCTTCCTCTCTTCAGGGAATACATTTTAGCTGCAGGATTG
WT CTGCCTGTCAGTAAGGCAAGTTTGGATTATTTGCTGAGCCAAACTGGTGTGGGTAACGCT
c858 CTGCCTGTCAGTAAGGCAAGTTTGGATTATTTGCTGAGCCAAACTGGTGTGGGTAACGCT
c858 CTGCCTGTCAGTAAGGCAAGTTTGGATTATTTGCTGAGCCAAACTGGTGTGGGTAACGCT
F
c858 Δ4,5
c858
G
WT GATTGGCACACACCTGAGAGAGGTGGCAGAAGAACAAAGTTTGTGAGAGGCTGGGAAGTATGGAAACACTTGCGAGACTATTTTCCTGTG
c862 GATTGGCACACACCTGAGAGAGGTGGCAGAAGAACAAAGTTTGTGAGAGGCTGGGAAGTATGGAAACACTTGCGAGACTATTTTCCTGTGGCCTTATTTTAA
WT AAGCTGGTAAAGACTGCTGAATTAAATCCCAATAAGAACTATATTATGGGCTGTCACCCGCAG
c862 CTTGCTATTTCTAGCTCTAAAACGTGAAGGTAACAAGACAAGCTGGTAAAGACTGCTGAATTAAATCCCAATAAGAACTATATTATGGGCTGTCACCCGCAG
WT GGCATCATGTGTTTTGGAGCCTTCTCCTGCTTCAGCACAGACCGCAATGGATTTGCGGAAACCTTTCCTGGAATACGGTCCACTCTTGCAATCTTAGCCGGA
c862 GGCATCATGTGTTTTGGAGCCTTCTCCTGCTTCAGCAGAGACCGCAATGGATTTGCGGAAACCTTTCCTGGAATACGGTCCACTCTTGCAATCT
WT CTTTTCCGCCTTCCTCTCTTCAGGGAATACATTTTAGCTGCAGGATTGCTGCCTGTCAGTAAGGCAAGTTTGGATTATTTGCTGAGCCAAACTGGTGTGGGT
c862 CCGCCTTCCTCTCTTCAGGGAATACATTTTAGCTGCAGGATTGCTGCCTGTCAGTAAGGCAAGTTTGGATTATTTGCTGAGCCAAACTGGTGTGGGT
H
c862

Figure S14: mogat3b CRISPR/Cas9 mutant details
(A) Depiction of the *mogat3b* gene structure (GRCz11; ENSDARG00000003635, transcript 201 (ENSDART00000015136.10)) highlighting the location of the two CRISPR target sequences in exon 4 & 5 (red arrows) and the enzymatic HPHG motif (yellow); not to scale, numbers indicate the length of introns and exons. Red line above the exons indicates the 276 bp deleted genomic region in the *c858* allele; the hashed region indicates the resulting deletions in the cDNA (red outline: deleted region that corresponds to the genomic deletion; black outline: all of exon 4 and 5 are spliced out). Purple line, text and caret below the exons indicate the locations of the 51 bp insertion and 13 bp deletion in the *c862* allele; the hashed region outlined in purple indicates the resulting deletion in the cDNA. Green arrows indicate gDNA genotyping primer binding for both alleles, blue arrows indicate cDNA primer binding for both alleles. Location of qPCR primers used in Figure 6D are shown in pink. (B) Example genomic DNA genotyping gel for the *mogat3b*^{c858} mutation. A 374 bp amplicon is generated for the wild-type allele using primers SF-MW-198F and SF-MW-199R, whereas the *c858* allele is 98 bp due to a deletion of 276 bp across exons 4-5. (C) Example genomic DNA genotyping gel for the *mogat3b*^{c862} mutation. A 374 bp amplicon is generated for the wild-type allele using primers SF-MW-198F and SF-MW-199R, whereas the *c862* allele is 412 bp due to an insertion of 51 bp in exons 4 and a 13 bp deletion in exon 5. The insertion consists of sequence from the guide RNA scaffold. (D) Example gel showing amplification of the cDNA sequence around the *c858* mutation results in two major cDNA products, at 175 bp and 87 bp. The c862 mutation results in one major cDNA product at 401 bp. (E & F) cDNA sequences and translations of wild-type *mogat3b* and *mogat3b*^{c858} deletion alleles from 199 – 561 bp (E), indicating both the in-frame deletion of exons 4 & 5 (middle, c.221_496del, p.Gly74_Ala165del) and the 188 bp deletion across exons 4 – 5 that results in a frame-shift and premature termination codon (bottom, c.271_458del, p.Arg91ProfsTer19). In both transcripts, the coding region for the HPHG motif in exon 5 is eliminated. Gray shading on the wild-type sequence indicates exons 3 & 5. Yellow, green and pink shading on the *c858* sequences corresponds to the regions shown in the trace files (F) from sanger sequencing of the cDNA to better highlight the deletions. (G & H) cDNA sequences and translation of wild-type *mogat3b* and the *mogat3b*^{c862} deletion allele from wild-type 199 – 555 bp (G), indicating the 51 bp insertion in exon 4 and 13 bp deletion in exon 5. The insertion causes a frame shift and premature termination codon (c.288_289insGCCTTATTTTAACTTGCTATTTCTAGCTCTAAAACGTGAAGGTAACAAGAC446_458del, p.Lys97AlafsTer4). Gray shading on the wild-type sequence indicates exons 3 & 5. Yellow, green and pink shading on the *c862* sequence corresponds to the region shown in the trace file (H) from sanger sequencing of the cDNA to better highlight the insertion and deletion.

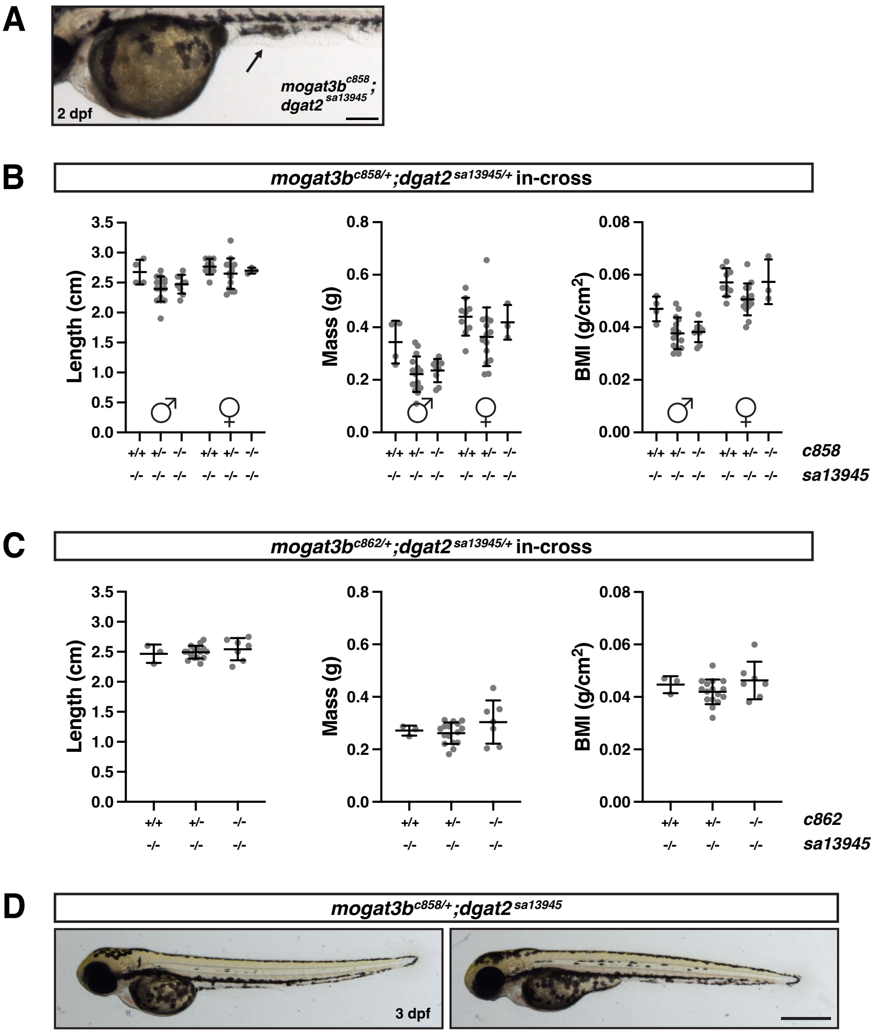

A
mogat3b c858 ; dgat2 sa13945
2 dpf
B
mogat3b c858/+ ;dgat2 sa13945/+ in-cross
Length (cm)
Mass (g)
BMI (g/cm2)
+/+ +/- -/- +/+ +/- -/-
-/- -/- -/- -/- -/- -/-
c858
sa13945
C
mogat3b c862/+ ;dgat2 sa13945/+ in-cross
Length (cm)
Mass (g)
BMI (g/cm2)
+/+ +/- -/-
-/- -/- -/-
c862
sa13945
D
mogat3b c858/+ ;dgat2 sa13945
3 dpf

Figure S15: mogat3b;dgat2 double mutants are similar in size to dgat2 mutants as adults. (A) Representative image of a *mogat3b*^{c858}*;dgat2*^{sa13945} double mutant with no yolk extension at 2 dpf. Arrows indicate where the YE should be located; tissue remnants of the YE are visible; Scale = 200 µm. (B,C) Standard length, mass and body mass index (BMI) data at 6 months of age for progeny of 1 in-cross of either *mogat3b*$^{c858/+}$*;dgat2*$^{sa13945/+}$ (B) or *mogat3b*$^{c862/+}$*;dgat2*$^{sa13945/+}$ (C) parents. Embryos exhibiting yolk opacity were selected prior to raising on the nursery and the *sa13945* genotype was confirmed with genotyping at time of measurement. For B, n = 54 total fish, split by sex and genotype 3 – 15 fish per group; mean ± SD, One-way ANOVA with Tukey's multiple comparisons test, $p = 0.0005$ (length), $p < 0.0001$ (mass), $p < 0.0001$ (BMI); no significant pairwise differences were found between genotypes, significant differences were only noted between sex and/or sex/genotype (not shown). For C, n = 26 total fish, sexes were combined because nearly all fish were male (22), 3 – 16 fish per genotype; mean ± SD, One-way ANOVA, $p = 0.6485$ (length), $p = 0.2367$ (mass), $p = 0.2070$ (BMI). (D) Representative images of *mogat3b*$^{c858/+}$*;dgat2*^{sa13945} mutants at 3 dpf either lacking the yolk extension (left) or with a short yolk extension (right); Scale = 500 µm.

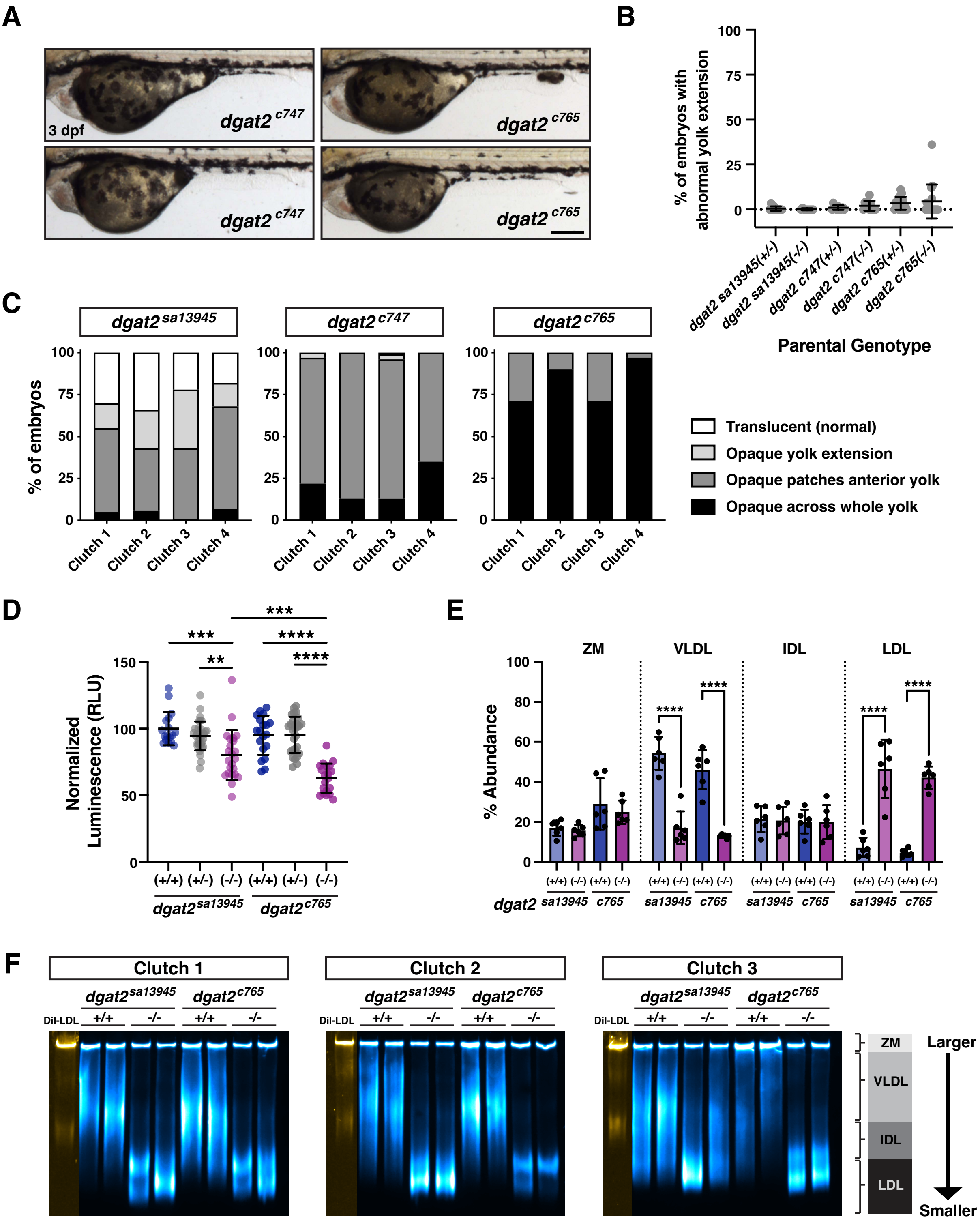

A
3 dpf
dgat2 c747
dgat2 c765
dgat2 c747
dgat2 c765
B
% of embryos with abnormal yolk extension
100
75
50
25
0
dgat2 sa13945(+/-)
dgat2 sa13945(-/-)
dgat2 c747(+/-)
dgat2 c747(-/-)
dgat2 c765(+/-)
dgat2 c765(-/-)
Parental Genotype
C
dgat2 sa13945
dgat2 c747
dgat2 c765
% of embryos
Clutch 1
Clutch 2
Clutch 3
Clutch 4
Translucent (normal)
Opaque yolk extension
Opaque patches anterior yolk
Opaque across whole yolk
D
Normalized Luminescence (RLU)
150
100
50
0
***
**
***
****
****
(+/+) (+/-) (-/-) (+/+) (+/-) (-/-)
dgat2 sa13945
dgat2 c765
E
ZM
VLDL
IDL
LDL
% Abundance
100
80
60
40
20
0
****
(+/+) (-/-) (+/+) (-/-)
dgat2 sa13945 c765
F
Clutch 1
Clutch 2
Clutch 3
dgat2 sa13945
dgat2 c765
DiI-LDL
+/+
-/-
ZM
VLDL
IDL
LDL
Larger
Smaller

Figure S16: *dgat2* CRISPR alleles can also present with abnormal yolk extension morphology
(A) Examples of *dgat2*^{c747} and *dgat2*^{c765} CRISPR/Cas9 mutant embryos with short or absent yolk extensions. Scale = 200 μm. (B) The percent of embryos per clutch exhibiting abnormal yolk extension morphology (lack of YE, short YE, broken YE) on 3 dpf from in-crosses of the noted parental genotypes (N = 8 – 18 clutches per genotype (clutch size ranged from 10 – 198 embryos). (C) Embryos from 4 in-crosses of *dgat2*$^{sa13945/+}$*, dgat2*$^{c747/+}$*, or dgat2*$^{c765/+}$ parents were imaged and scored at 3 dpf for the degree of yolk opacity, binned into the four noted categories and expressed as a percent of total embryos per genotype. (D) Normalized LipoGlo luminescence (RLU = relative luminescence units) in *dgat2*^{sa13945} and *dgat2*^{c765} mutants and siblings at 3 dpf. Results represent pooled data from 3 independent clutches per *dgat2* allele, n = 17 – 33 embryos per genotype; mean ± SD. To allow for a more accurate comparison between *dgat2* alleles, embryos from both alleles were assayed at the same time; for example, the embryos from *dgat2*^{sa13945} clutch 1 and *dgat2*^{c765} clutch 1 were assayed in the same 96-well plate. Significance was determined with a One-way ANOVA, Tukey's multiple comparisons tests were performed to compare genotypes at each day of development, and p values were adjusted to control for multiple comparisons, for clarity, only selected significance tests are shown (** $p<0.01$, *** $p<0.001$, ****$p<0.0001$). (E & F) Quantitation of B-lp size distribution from whole embryo lysate and associated LipoGlo PAGE gels from *dgat2*^{sa13945} and *dgat2*^{c765} mutants and WT siblings at 3 dpf. As in Figure 4 and Supplemental Figure 6, B-lps are divided into four classes based on mobility, including zero mobility (ZM), very low density lipoproteins (VLDL), intermediate density lipoproteins (IDL) and low density lipoprotein (LDL). Graph show B-lp subclass % abundance, analyzed as described in (11), note the diI-LDL used here for gel calibration was from a different product lot # as in Supplemental Figure 6, thus the bin cut-off values for each subclass reflect this differential calibration. Results represent pooled data from 3 independent clutches per *dgat2* allele, n = 6 embryos/genotype; mean ± SD. Significance was determined with a One-way ANOVA, Šídák's multiple comparisons tests were performed to compare genotypes, and p values were adjusted to control for multiple comparisons, for clarity, only selected significance tests are shown (****$p<0.0001$).

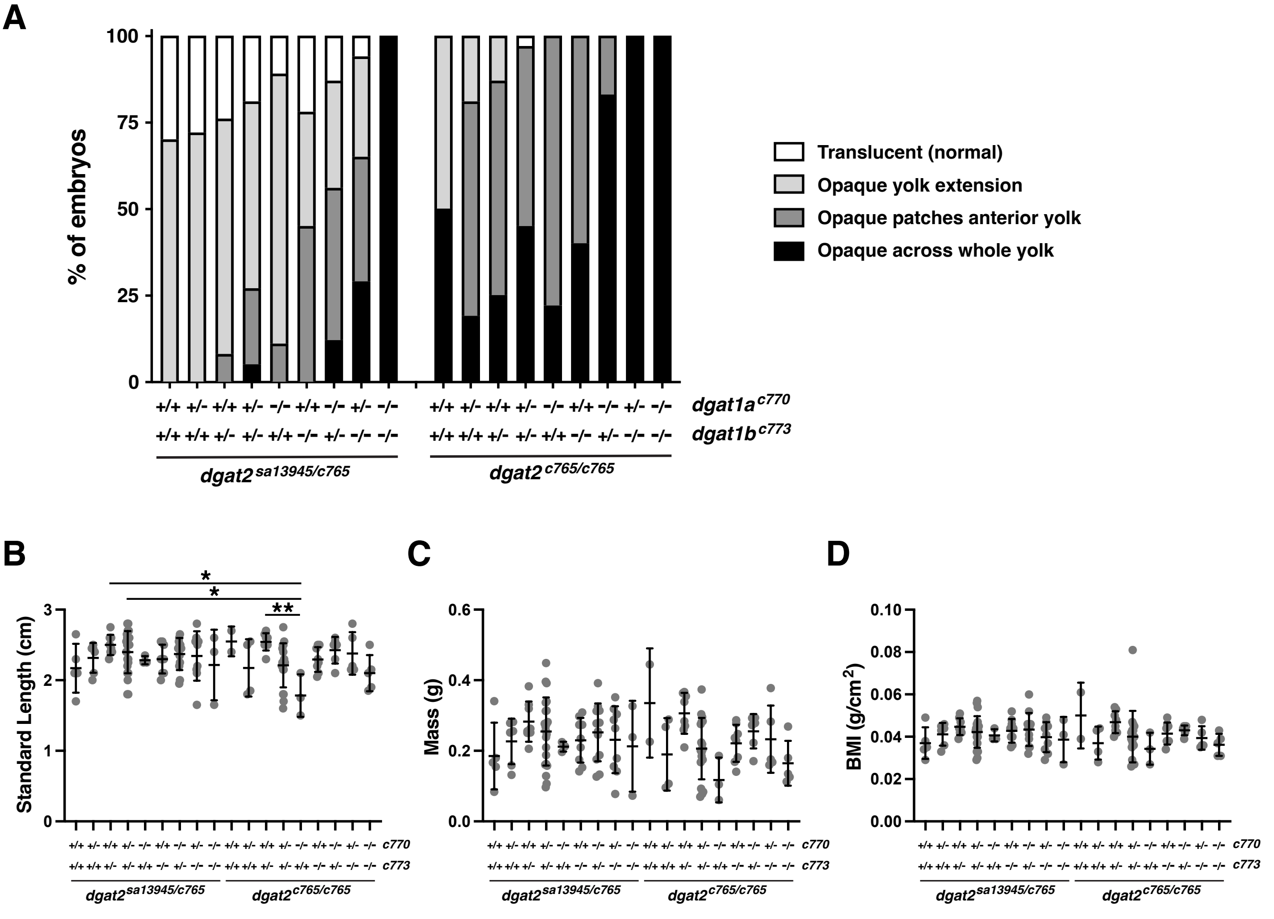
A
% of embryos
100
75
50
25
0
Translucent (normal)
Opaque yolk extension
Opaque patches anterior yolk
Opaque across whole yolk
+/+ +/- +/+ +/- -/- +/+ -/- +/- -/- +/+ +/- +/+ +/- -/- +/+ -/- +/- -/-
dgat1a c770
+/+ +/+ +/- +/- +/+ -/- +/- -/- -/- +/+ +/+ +/- +/- +/+ -/- +/- -/- -/-
dgat1b c773
dgat2 sa13945/c765
dgat2 c765/c765
B
Standard Length (cm)
*
*
**
C
Mass (g)
D
BMI (g/cm2)
c770
c773

Figure S17: The *dgat2*^{c765} allele influences yolk opacity in embryos but does not affect adult size.

(A) Embryos from 3 crosses of *dgat1a*$^{c770/+}$*;dgat1b*^{c773}*;dgat2*$^{sa13945/c765}$ x *dgat1a*^{c770}*;dgat1b*^{c773}*;dgat2*^{c765} parents were imaged and scored at 3 dpf for the degree of yolk opacity, binned into the four noted categories and expressed as a percent of total embryos per genotype (n = 291 fish total, 6 – 37 fish per genotype). (B – D) Standard length, mass and body mass index (BMI) data for progeny of 2 crosses of *dgat1a*$^{c770/+}$*;dgat1b*^{c773}*;dgat2*$^{sa13945/c765}$ x *dgat1a*^{c770}*;dgat1b*^{c773}*;dgat2*^{c765} parents at 3 months of age (n = 136 total fish, 2 – 22 fish per genotype); mean ± SD, One-way ANOVA, p = 0.0121 for (B), p = 0.0397 for (C) and p = 0.329 for (D). Tukey's multiple comparisons tests were performed to compare genotypes, * p <0.05, ** p <0.01.

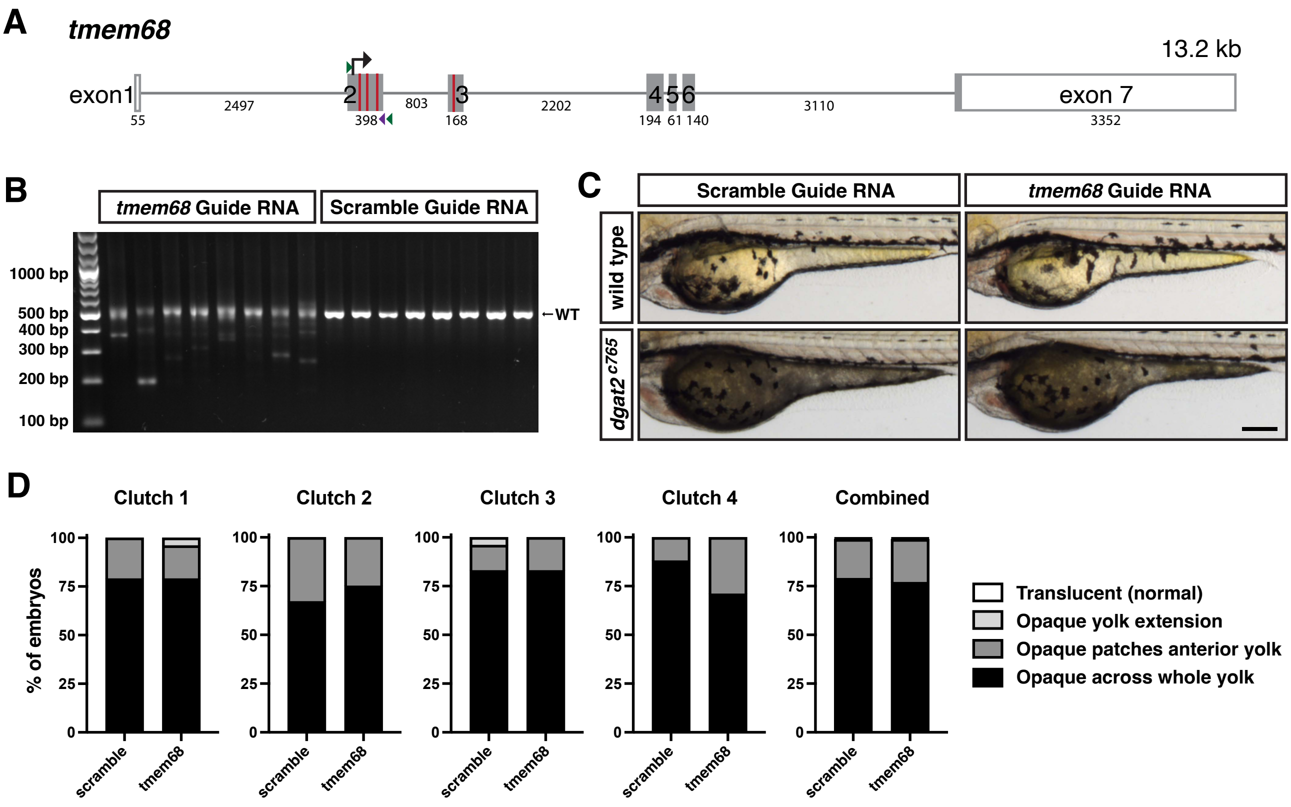

A
tmem68
13.2 kb
exon1
2
3
456
exon 7
55
2497
398
803
168
2202
194 61 140
3110
3352
B
tmem68 Guide RNA
Scramble Guide RNA
1000 bp
500 bp
400 bp
300 bp
200 bp
100 bp
←WT
C
Scramble Guide RNA
tmem68 Guide RNA
wild type
dgat2 c765
D
Clutch 1
Clutch 2
Clutch 3
Clutch 4
Combined
% of embryos
100
75
50
25
0
scramble
tmem68
Translucent (normal)
Opaque yolk extension
Opaque patches anterior yolk
Opaque across whole yolk

Figure S18: Mutations in *tmem68* do not rescue yolk opacity in *dgat2* mutants
(A) Depiction of the *tmem68* gene structure (GRCz11; ENSDARG00000086737, transcript 202 (ENSDART000000188261.1)) highlighting the location of the four CRISPR target sequences in exon 2 & 3 (red lines). Green arrowheads indicate gDNA genotyping primer binding used for the gel, purple arrowhead indicates the gDNA reverse primer location used for Amplicon sequencing, numbers represent bp lengths of exons and introns. (B) Fish were injected at the 1-cell stage with Cas9 protein and either a mixture of four CRISPR guide RNAs targeting *tmem68* or a mixture of four non-targeting scramble guides. At 3 dpf, fish were sacrificed and genomic DNA was extracted, a region around exon 2 was amplified using primers SF-MW-286F and SF-MW-287R. The expected WT amplicon length is 507 bp, however, in the fish injected with guide RNA against *tmem68*, amplicon sizes of variable length are detected, indicating successful Cas9 nuclease activity. Lanes represent PCR amplicons from individual injected fish. To characterize some of the indels present in the injected fish, the samples shown on the gel were also used for Amplicon sequencing. A smaller region around exon 2 was amplified using primers SF-MW-286F and SF-MW-319R (expected WT amplicon length is 400 bp), samples were pooled and submitted to Genewiz for Amplicon-EZ next-generation sequencing and analysis. While 96.6% of reads from the *tmem68* guide RNA injected fish exhibited indels (65.6% of which resulted in a frame shift), only 1.43% of reads in the Scramble guide RNA sample had indels with 1.39% of these resulting in a shift in the reading frame. We hypothesize that the base changes noted in the Scramble sequencing data are either natural polymorphisms (C>T at position 36 and G>A at position 243) or occurred during PCR amplification as we did not use a proofreading polymerase. For further details, refer to Supporting Information File 3. (C) Wild-type and *dgat2*^{c765} embryos were injected with the *tmem68* and scramble guide RNAs and imaged at 3 dpf. The *tmem68* guide RNAs did not alter yolk translucence or morphology of wild-type fish, and did not rescue the yolk opacity in the *dgat2*^{c765} mutants. Scale = 200 µm. (D) Images were assessed for the degree of yolk opacity into the four denoted bins and graphed as a percent of total embryos/treatment group (n = 24 fish per treatment group per clutch, N = 4 clutches; Combined data from all four clutches is also presented).

References

1. Fraher, D., Sanigorski, A., Mellett, N. A., Meikle, P. J., Sinclair, A. J., and Gibert, Y. (2016) Zebrafish Embryonic Lipidomic Analysis Reveals that the Yolk Cell Is Metabolically Active in Processing Lipid. *Cell Rep* **14**, 1317-1329
11. Thierer, J. H., Ekker, S. C., and Farber, S. A. (2019) The LipoGlo reporter system for sensitive and specific monitoring of atherogenic lipoproteins. *Nat Commun* **10**, 3426
70. Clark, E. M., Nonarath, H. J. T., Bostrom, J. R., and Link, B. A. (2020) Establishment and validation of an endoplasmic reticulum stress reporter to monitor zebrafish ATF6 activity in development and disease. *Dis Model Mech* **13**
144. Schmittgen, T. D., and Livak, K. J. (2008) Analyzing real-time PCR data by the comparative C(T) method. *Nat Protoc* **3**, 1101-1108
151. Alam, M., Gilham, D., Vance, D. E., and Lehner, R. (2006) Mutation of F417 but not of L418 or L420 in the lipid binding domain decreases the activity of triacylglycerol hydrolase. *J Lipid Res* **47**, 375-383
152. Au-Young, J., and Fielding, C. J. (1992) Synthesis and secretion of wild-type and mutant human plasma cholesteryl ester transfer protein in baculovirus-transfected insect cells: the carboxyl-terminal region is required for both lipoprotein binding and catalysis of transfer. *Proc Natl Acad Sci U S A* **89**, 4094-4098
153. Hillary, R. F., and FitzGerald, U. (2018) A lifetime of stress: ATF6 in development and homeostasis. *J Biomed Sci* **25**, 48
154. Jurkowitz, M. S., Horrocks, L. A., and Litsky, M. L. (1999) Identification and characterization of alkenyl hydrolase (lysoplasmalogenase) in microsomes and identification of a plasmalogen-active phospholipase A2 in cytosol of small intestinal epithelium. *Biochim Biophys Acta* **1437**, 142-156
155. Wu, L. C., Pfeiffer, D. R., Calhoon, E. A., Madiai, F., Marcucci, G., Liu, S., and Jurkowitz, M. S. (2011) Purification, identification, and cloning of lysoplasmalogenase, the enzyme that catalyzes hydrolysis of the vinyl ether bond of lysoplasmalogen. *J Biol Chem* **286**, 24916-24930
156. Magnusson, C. D., and Haraldsson, G. G. (2011) Ether lipids. *Chem Phys Lipids* **164**, 315-340
157. Ma, Z., Onorato, J. M., Chen, L., Nelson, D. W., Yen, C. E., and Cheng, D. (2017) Synthesis of neutral ether lipid monoalkyl-diacylglycerol by lipid acyltransferases. *J Lipid Res* **58**, 1091-1099
158. Gotfryd, K., Mosca, A. F., Missel, J. W., Truelsen, S. F., Wang, K., Spulber, M., Krabbe, S., Helix-Nielsen, C., Laforenza, U., Soveral, G., Pedersen, P. A., and Gourdon, P. (2018) Human adipose glycerol flux is regulated by a pH gate in AQP10. *Nat Commun* **9**, 4749
159. Laforenza, U., Scaffino, M. F., and Gastaldi, G. (2013) Aquaporin-10 represents an alternative pathway for glycerol efflux from human adipocytes. *PLoS One* **8**, e54474
160. Li, H., Kamiie, J., Morishita, Y., Yoshida, Y., Yaoita, E., Ishibashi, K., and Yamamoto, T. (2005) Expression and localization of two isoforms of AQP10 in human small intestine. *Biol Cell* **97**, 823-829
161. Qin, Y., Wang, S., Duan, X., and Liu, D. (2019) Expression analysis of the aquaporins during zebrafish embryonic development. *Gene Expr Patterns* **32**, 38-43
162. Tingaud-Sequeira, A., Calusinska, M., Finn, R. N., Chauvigne, F., Lozano, J., and Cerda, J. (2010) The zebrafish genome encodes the largest vertebrate repertoire of functional aquaporins with dual paralogy and substrate specificities similar to mammals. *BMC Evol Biol* **10**, 38
